# Feasibility of controlled nitric oxide generation *via* ascorbate induced chemical reduction of nitrite ions†

**DOI:** 10.1039/d4dt01980f

**Published:** 2024-11-15

**Authors:** Hannah J. Naldrett, Csilla Fekete, Robert H. Bartlett, Zoltán Benkő, Steven P. Schwendeman, Gergely Lautner

**Affiliations:** a Department of Pharmaceutical Sciences, University of Michigan Ann Arbor MI 48109 USA schwende@umich.edu glautner@umich.edu; b Department of Inorganic and Analytical Chemistry, Budapest University of Technology and Economics Budapest Hungary; c Department of Surgery, ECLS Laboratory, University of Michigan Medical School Ann Arbor MI 48109 USA; d HUN-REN-BME Computation Driven Chemistry Research Group Budapest Hungary; e Biointerfaces Institute, University of Michigan Ann Arbor MI 48109 USA; f Department of Biomedical Engineering, University of Michigan Ann Arbor MI 48109 USA

## Abstract

Inhalable nitric oxide (iNO) is a lifesaving, FDA-approved drug to improve oxygenation in persistent pulmonary hypertension of the newborn. iNO also has many other applications in lung diseases owing to its vasodilatory and antimicrobial effects. However, its wider therapeutic application is often prohibited by the high cost and logistical barriers of traditional NO/N_2_ gas tanks. Development of low-cost, portable and tankless nitric oxide (NO) generators is a critical need to advance iNO therapy. Here, we describe the feasibility of NO generation by the controlled reduction of nitrite (NO_2_^−^) ions. This was accomplished by using ascorbate to reduce NO_2_^−^ ions mediated by a copper(I/II) redox pair complexed by an azo-crown ether ligand ([Cu(ii)L]^2+^/[Cu(i)L]^+^) in the solution phase. We found that oxalate, a decomposition product of ascorbate, interferes with the NO generation from the copper-ligand complex. This interference was mitigated, and the reaction was further optimized. NO generation through this method was found to be highly controllable *via* its proportionality to the flow rate of NO_2_^−^ injected into a reaction chamber containing the reducing components. Hence, this simple approach adds to the current collection of innovative methods under development to obviate the use of NO tanks for iNO delivery.

## Abstract 2

Controlled generation of nitric oxide (NO) by continuous infusion of nitrite ions into an ascorbate-reduced copper catalyst mediator.

## Graphical abstract

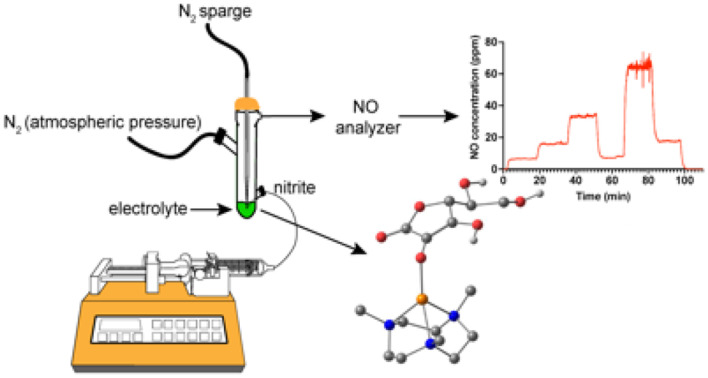

## Introduction

Endogenous NO is essential to the maintenance of cardiovascular homeostasis. Its vasodilatory, anti-platelet and antimicrobial effects make NO a potent therapeutic agent.^1^ INO is a lifesaving and FDA-approved treatment to improve oxygenation in persistent pulmonary hypertension of the newborn (PPHN),^2^ and is also used off-label in the therapy of cystic fibrosis, acute bronchiolitis, and as a rescue therapy in patients with COVID-19 and acute respiratory distress syndrome (ARDS).^1,3–6^ However, the high costs and the logistics of traditional medical NO/N_2_ gas tanks pose a substantial barrier to expanding iNO therapies for these newer applications. Therefore, development of low-cost and more portable tankless NO generators has become increasingly important to increase the availability of iNO for more patients.^7–11^

Currently, medical grade NO gas for inhalation therapy is traditionally administered from pressurized gas cylinders containing low concentrations (400 ppm, 800 ppm or 4880 ppm) of NO balanced with N_2_. The dilution inhibits disproportionation of NO to form nitrous oxide (N_2_O) and nitrogen dioxide radical (NO_2_) during storage.^12,13^ In addition to the gas cylinder itself, cylinder-based systems require the use of a mass flow controller-based gas delivery system to ensure the delivery of the desired NO concentration and flow rate. A gas monitor is required for measuring the delivered NO levels and for monitoring the formation of unwanted NO_2_, a contaminant that is mainly formed from the blending of the NO gas with the O_2_^−^ containing ventilation gas.^11^

Several approaches to create portable and tankless iNO delivery include chemical reduction of NO_2_ gas, corona discharge, copper catalyzed electrochemical reduction of nitrite, and photolytic reduction of *S*-nitrosothiols.^11,14,15^ Vero's FDA-approved chemical NO generation method works by generating gaseous NO_2_ from liquid N_2_O_4_, then reducing it to NO using an ascorbic acid impregnated silica gel catalytic converter.^11^ This innovative approach obviates the need for bulky gas cylinders owing to the molecular density of N_2_O_4_. However, implementation of this approach carries the drawback of requiring an extensive production and supply chain to produce the necessary liquid N_2_O_4_.^11^ Third Pole's NO generation method uses a pulsed corona discharge to generate NO directly from air. This method also generates significant NO_2_ (2% of the NO generated), so a major consideration in device development is controlling the formation of NO_2_.^8^ A major drawback of this technique is that NO production is limited by the interdependence of airflow and NO production. That is, as airflow increases, NO production decreases, so that the amount of NO produced may not be sufficient for certain applications that require a high concentration of NO at high flows. The NO device developed by Third Pole using this method is in the process of obtaining FDA approval.^11^

One alternative tankless method is the photolytic generation of NO from light sensitive *S*-nitrosothiols (RSNO), such as *S*-nitroso-*N*-acetyl penicillamine (SNAP) or *S*-nitrosoglutathione (GSNO).^15–17^ Feedback controlled LED lights can release precisely controlled levels of NO from the silicone rubber embedded RSNO into the N_2_ recipient gas to be blended with the ventilation gas. Although this method is very simple and controllable, it is currently better suited for low-level NO approaches.

Another alternative to these new methods of NO production is the copper-ligand mediated reduction of dissolved nitrite ions to NO. This reduction is a nature-inspired process where nitrite reductase enzymes reduce nitrite to NO; one class of these enzymes have copper-containing active sites.^18^ The ascorbate-mediated reduction of nitrite to NO by biomimetic catalysts based on the active site of these enzymes has been studied in methanol.^19,20^ The Meyerhoff group used a copper ion containing catalyst to produce NO from the nitrite present in blood to create an anticoagulant effect at blood-contacting polymer surfaces. Ascorbate, also present in the blood, was used as a reducing agent to reduce Cu(ii) to the active Cu(i).^21^ The copper-ligand complex was embedded into a polymer, while the nitrite and ascorbate were in the electrolyte contacting the polymer surface. The amount of NO generated by that copper-doped polymer was limited by mass transport and practically diminished in the presence of oxygen.

Later, the same group used copper ions chelated to 1,4,7-trimethyl-1,4,7-triazacyclononane (Me_3_TACN = L) as the electron transfer complex in the solution phase, where Cu(ii) is reduced to Cu(i) on the surface of an inert working electrode, which can then reduce nitrite to NO, leaving behind [Cu(ii)L]^2+^ to be converted back to the active [Cu(i)L]^+^ on the electrode. The amount of NO generated is controlled by varying the amount of current and thus the amount of [Cu(i)L]^+^ available to react with the large pool of available nitrite in the electrolyte.^14^ This electromediated system was tested in NO-releasing antimicrobial catheters,^22^ and also for generating NO in extracorporeal applications,^14,23–26^ after removing the NO gas from the electrolyte using a liquid–gas contactor into a nitrogen stream. This method has the advantage of producing very high levels of NO from small volumes of solutions containing nitrite salts.

Here we explored whether instead of the electrochemical reduction of [Cu(ii)L]^2+^, we could use ascorbate to reduce the [Cu(ii)L]^2+^ in the solution phase for generating NO. By controlling the availability of a limiting reactant with the remainder of the electrolyte components, NO should be produced in a controlled fashion by the simple mixing of two reactive solutions together. A series of experiments were conducted to develop this approach and to verify its functionality, as described below.

## Experimental

### Materials

Calcium l-ascorbate dihydrate (99.5%, Spectrum Chemical MFG Corp), sodium l-ascorbate (>98.0%, TCI), l-ascorbic acid (99%, Sigma Aldrich), copper(ii) sulfate (98%, Sigma Aldrich), sodium nitrite (≥99.9%, Sigma Aldrich), 1,4,7-trimethyl-1,4,7-triazacyclononane (98%, Matrix Scientific), MES (≥99%, Sigma Aldrich), HEPES (≥99.5%, Sigma Aldrich), PBS tablets (P4417, Sigma Aldrich), nitrogen gas (Ultra High Purity, Metro Welding Supply Corp), calcium chloride dihydrate (≥99%, Sigma Aldrich), l-cysteine hydrochloride monohydrate (>98%, Sigma Aldrich), l-glutathione reduced (98%, Sigma), sodium oxalate (≥99%, Sigma Aldrich), dehydroascorbic acid (261556, Sigma Aldrich). All other common reagents, *e.g.*, for adjusting pH, were reagent grade from Sigma Aldrich. Sodium threonate was prepared using a process described earlier from calcium l-threonate (>98%, TCI America).^27^

### Computational methods

The calculations were performed using the Gaussian 16 program package.^28^ The geometry optimizations were carried out using the ωB97X-D range-separated functional employing the Polarizable Continuum Model (PCM) with water as a solvent to simulate the solvent effects. The local minima on the potential energy surface were verified using harmonic vibration analysis (zero negative force constants). To simulate the UV-Vis spectra of selected species, time-dependent density functional (TD-DFT) calculations involving the first 30 excitations were applied using the B3LYP method, in combination with PCM simulation of solvent effects. To test the reliability of this method, we used the M06 functional for selected complexes. Note that these methods were shown to successfully describe the UV-Vis spectra of Cu(ii) complexes before.^29,30^ In general, both functionals deliver rather similar results; the results obtained at the TD-B3LYP/def2-SVP//ωB97X-D/def2-SVP level of theory will be discussed.

### Electrolyte composition

For experiments where ascorbate was injected the composition was used as previously described for electrocatalytic NO generation: 7 mM Me_3_TACN, 7 mM copper sulfate (CuSO_4_) and 1 M sodium nitrite (NaNO_2_) buffered in pH 7.3 0.5 M HEPES solution.^14^ The electrolyte was made by mixing the components at room temperature until dissolved.^14^ The pH was adjusted with sodium hydroxide pellets.

For experiments where nitrite was injected or infused, the electrolyte was prepared by mixing 7 mM Me_3_TACN, 7 mM copper sulfate (CuSO_4_), the reducing agent and any additional components specified until dissolved. The pH was adjusted with sodium hydroxide pellets.

### Measurement of generated NO

NO generation was measured using a Sievers 280i ozone chemiluminescent NO analyzer (Boulder, CO) connected to a glass reaction cell. The Sievers 280i instrument was used with a 200 mL min^−1^ flow restrictor. Measurements were performed using 5 mL of electrolyte solution, which was kept well stirred through vigorous N_2_ sparging (∼150 mL min^−1^) because, as with other similar copper-containing complexes, Cu(i)L is oxygen sensitive.

NO concentrations in the gas phase are reported as molar ratios in parts per million (ppm) or parts per billion (ppb). Conversion efficiencies are reported as the ratio of the amount of generated NO per amount of nitrite added.

### NO generation experiments

All experiments were performed at room temperature. The ascorbate salt or the NaNO_2_ was dissolved in the same buffer as the electrolyte and was injected with a Hamilton syringe or infused using a Cole Parmer 74 900 syringe pump with disposable 3 mL or 10 mL plastic syringe *via* a stainless-steel needle into the electrolyte through a septum.

### UV-vis experiments

2.25 mL of electrolyte solution of 1.11 mM [Cu(ii)L]^2+^ (with or without 1.11 mM NaNO_2_) was prepared, then the solution was deoxygenated by sparging with N_2_ gas in a septum sealed cuvette for approximately 10 minutes. A separate solution of 400 mM sodium ascorbate was similarly deoxygenated in a septum sealed vial. An initial UV-vis reading was taken of the electrolyte prior to the addition of ascorbate, then a gas-tight Hamilton syringe was used to transfer 0.25 mL of the ascorbate solution to the sealed cuvette with the electrolyte, for a final electrolyte composition of 1 mM [Cu(ii)L]^2+^ (with or without 1 mM NaNO_2_) and 40 mM sodium ascorbate. The contents of the cuvette were mixed briefly by inversion, then the spectra were taken at the time intervals specified in Fig. S2.† UV-vis spectra were recorded using Molecular Devices SpectraMax M2 or M3 and Brand semi-micro UV cuvette or Firefly quartz macro cuvette with septum cap.

### Electrolyte storage stability experiments

The electrolyte was stored in airtight amber glass vials to protect the ascorbate from decomposition by light and at ambient temperature. After each measurement of NO generation, the 5 mL of electrolyte used was returned to the amber vial, and all 15 mL of electrolyte was deoxygenated by purging with N_2_ for 15 min to replicate the oxygen-free environment of the NO generation reaction cell for storage.

### Measurement of N_2_O

N_2_O was measured with a Shimadzu IRTracer-100 FT-IR instrument equipped with a stainless-steel gas cell with 10 m pathlength and ZnSe windows accessory (PIKE Technologies). We injected 1 mL of 10 mM sodium nitrite into 25 mL of electrolyte containing 3.5 mM calcium ascorbate, 14 mM CaCl_2_ and 7 mM [Cu(ii)L]^2+^. The solution was sparged with N_2_ gas through a glass frit to remove the generated gases from the solution phase and to deliver them to the FT-IR gas cell.

## Results and discussion

### NO generation by [Cu(i)L]^+^ mediated chemical reduction

**Fig. 1:**
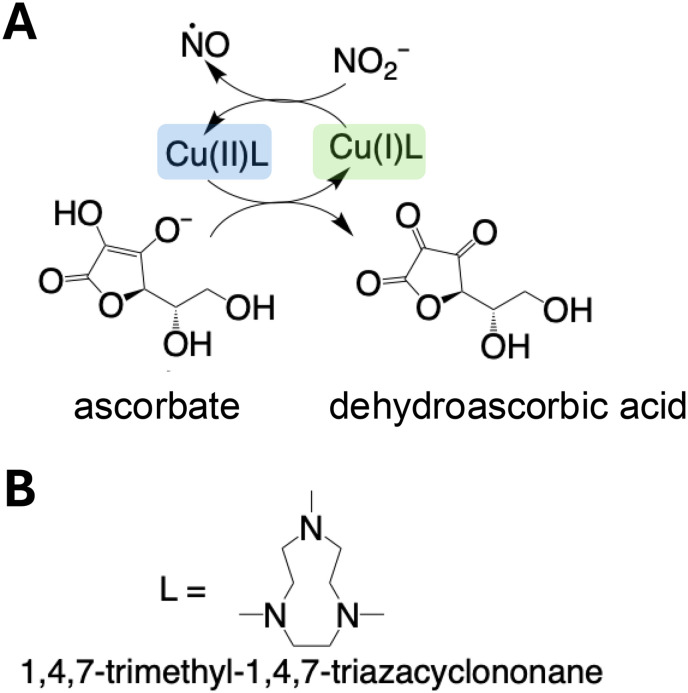
Schematic diagram illustrating (A) the proposed method of NO generation and (B) the structure of the ligand (L).

**Fig. 2:**
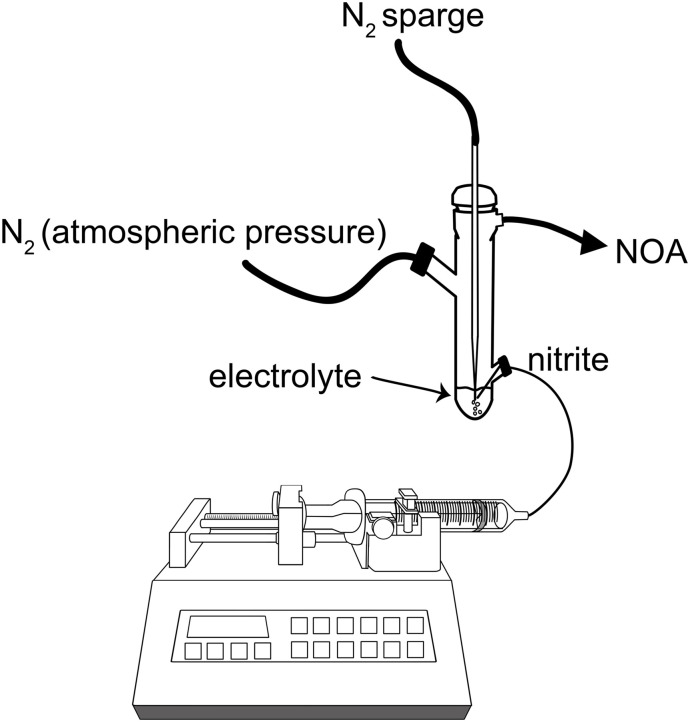
Schematic of the experimental setup for NO generation through nitrite infusion into electrolyte.

The first attempt to generate NO by [Cu(i)L]^+^-mediated chemical reduction (illustrated in Fig. 1A) was done by infusing 0.28 M ascorbic acid at 1 μL min^−1^ into a reaction chamber containing 5 mL of the ligand (L, 7 mM) (Fig. 1B) coordinated to Cu(ii) and the nitrite (1 M). Although we observed NO generation (>50 ppm), the NO release showed low responsiveness to ascorbic acid infusion, as it took hours for NO to return to baseline after the infusion was stopped (Fig. S1†). To compensate for this, instead of infusing ascorbic acid, NaNO_2_ was used as the limiting reactant and infused into the [Cu(ii)L]^2+^ and ascorbate containing electrolyte, using the experimental setup illustrated in Fig. 2. Of note, upon the addition of ascorbate to the electrolyte, there was a distinct color change from blue to green.

To understand the time dependence of the observed reactions better, we monitored the reactions between [Cu(ii)L]^2+^ and a large excess of sodium ascorbate in both the absence (Fig. S2A†) and the presence (Fig. S2B†) of nitrite ions. In both cases, there is an increase in absorbance at 400 nm (Fig. S2C†) and at 630 nm (Fig. S2D†) upon the addition of the ascorbate to the [Cu(ii)L]^2+^ complex, as well as a decrease at 660 nm (Fig. S2D†). For the nitrite-containing electrolyte, the signal at 400 nm increases at a slightly higher initial rate during the first 8 hours after the addition of the ascorbate, compared to the rate of increase observed in the absence of nitrite. Otherwise, the time dependency of the change in absorbance at the other wavelengths is similar in both cases. To shed more light on these changes, we performed TD-DFT (time dependent density functional theory, for details see the Experimental section) calculations to describe the UV-Vis spectra of species that can possibly be present in the solutions (Table S1†). Although such calculations do not allow for precise identification of species, our goal is to compare the experimental and computed spectra, which may help in understanding the changes in absorption properties and identifying possible charge transfer effects. As a starting point, the [Cu(ii)L]^2+^ cation (complexed by one to three water molecules) exhibits transitions in the 650 to 745 nm range with rather low oscillator strengths (below *f* = 0.001), in line with the experimentally observed very broad band with low intensity (Fig. S2†). Following the addition of the ascorbate to the solution of [Cu(ii)L]^2+^, we suggest that the increase observed at 660 nm is caused by the coordination of an ascorbate ion to the Cu(ii) center. According to previous computational studies,^31,32^ the most probable coordination modes of either neutral [Cu(ii)L(Asc)] or cationic [Cu(ii)L(HAsc)]^+^ complexes were computed. These complexes indeed show transitions between 600 and 745 nm with remarkable oscillator strengths (in the range *f* = 0.01 to 0.04), which are consistent with a pronounced charge transfer character, and is nicely observable on selected orbitals involved in these transitions (Fig. S3a†). The band at 660 nm shows a slow decrease in intensity over time in the absence of nitrite ions after adding ascorbate ions to the solution (Fig. S2D†), indicating the very slow disappearance of Cu(ii) species. According to the TD-DFT calculations, a complex of Cu(i) formed with one of the primary decomposition products of ascorbate, dehydroascorbic acid (DHA), [Cu(i)L(DHA)]^+^ exhibits intense transitions with marked charge transfer character unexpectedly around 567 to 687 nm (Table S1 and Fig. S3b†) offering a possible explanation for the absorption band around 630 nm (Fig. S2†), but based on the complexity of the reaction network, we cannot exclude other possibilities.

In contrast, the increasing absorptions at 400 nm (Fig. S2C†) may arise from the formation of Cu(i)-containing species that have absorption in these ranges. Therefore, we tested several Cu(i)-containing species by TD-DFT calculations, and most of them (*e.g.*, the [Cu(i)L(HAsc)] complex, or the [Cu(i)L(H_2_O)]^+^ ion) present low energy transitions in the range 300–345 nm of rather high intensity (*f* ∼0.01). Considering the computational error (commonly 30–50 nm), these transitions can explain the appearance of the broad band at 400 nm.

Based on the time dependencies of the intensity of the signals at 400 and 660 nm, we postulate that first the DHA complex of [Cu(i)L]^+^ is formed by the reduction of Cu(ii), which then undergoes a ligand exchange to ascorbate. This [Cu(i)L(HAsc)] complex forms as a secondary product (with zero initial rate) in a slow reaction following pseudo zeroth order kinetics (due to the large excess of ascorbate ions).

The situation is similar when nitrite ions are present from the beginning; however, we observed an important difference: the signal at 400 nm has a different time dependence, which we assign to the formation of the nitrite complex [Cu(i)L]NO_2_. This complex is expected to form from the DHA complex (absorbing at 660 nm) and has an estimated absorption wavelength at 349 nm (TD-DFT). According to the UV-vis monitoring, the formation of this complex is much faster than that of the ascorbate complex of Cu(i), and the first order kinetics are caused by the lower amount (equimolar to the initial Cu(ii)) compared to the large amount of ascorbate. After the nitrite ions are consumed, the time dependence of the intensities does not change compared to the case without nitrite, indicating that the rate determining step is the reduction of Cu(ii).

### Optimization of electrolyte composition

We found that electrolyte made with calcium ascorbate instead of sodium ascorbate increased the NO generation (Fig. S4†). We also observed that a decomposition product of ascorbate, oxalate,^33^ had an effect on NO generation. We added a concentration of oxalate equivalent to the ascorbate concentration to the electrolyte, and the NO formation was completely inhibited (Fig. S5†). Ascorbic acid retains a maximum stability below pH 6.0.^34^ Thus, decreasing the pH to 5.8 (0.5 M MES) was expected to be beneficial for stabilizing the ascorbate in the electrolyte and minimizing oxalate formation. As a result of experimentation with different buffer systems, we found that pH 5.8 increased the responsiveness (Fig. S6,† left) and conversion efficiency (Fig. S6,† right) of NO generation. It is known that during acid induced nitrite reduction, ascorbic acid can prevent N_2_O_3_ formation in anaerobic conditions and facilitate NO formation,^35^ however at pH 5.8, there was not any reasonable amount of NO generation detectable without the [Cu(ii)L]^2+^, indicating that any NO generation that occurs at pH 5.8 is indeed copper ion mediated (Fig. S6†).

Additionally, to ensure that complexation of the copper ion with the ligand was necessary to generate NO, a solution of CuSO_4_ and calcium ascorbate was made without ligand present. The uncoordinated Cu(ii) ions immediately reacted with the ascorbate and precipitated out of the solution as an insoluble yellow solid (for FT-IR spectrum see Fig. S7†). The yellow precipitate has distinctly lower nitrite-reducing activity to generate NO compared to the [Cu(ii)L]^2+^ (Fig. S6,† left). The ligand is therefore necessary to maintain the solubility and thus the rapid and responsive catalytic activity of the copper ions.

To further counteract the above-mentioned undesired effects of oxalate, the electrolyte was prepared with calcium ascorbate (7–140 mM) to remove any oxalate formed from the decomposition of ascorbate by precipitation as a practically insoluble calcium salt. The precipitate was identified as calcium oxalate based on the asymmetric stretching (*v*_a_) and symmetric stretching (*v*_s_) oxalate fundamental vibrations assigned to the bands at 1631 and 1321 cm^−1^,^36^ respectively, on the FT-IR ATR spectrum (Fig. S8†). Once precipitated out of solution, the solid calcium oxalate was no longer able to interfere with the NO generation. However, at higher calcium ion concentrations, the calcium ions appeared to outcompete the copper ions for binding with the ligand and a loss of NO generation was observed (Fig. S9†). This negative effective was not seen until the concentration of calcium ions was over 1 M (Fig. S9†).

A summary of the optimization process of the electrolyte composition is detailed in Table S2.† Within the investigated parameter space, the optimal formulation of the electrolyte for NO generation consisted of 7 mM [Cu(ii)L]^2+^, and 140 mM calcium ascorbate, buffered by 0.5 M MES at pH 5.8.

Of note, even with the optimized parameters, when the ascorbate was injected into the nitrite-containing electrolyte, similar to what we attempted in one of our earliest attempts (Fig. S1†), we still observed sluggish response (Fig. S10†). This system of NO generation relies on two reactions occurring in sequence: the reduction of [Cu(ii)L]^2+^ to [Cu(i)L]^+^ by the ascorbate and the reduction of nitrite to NO by the [Cu(i)L]^+^. When ascorbate is infused into the electrolyte that already contains the nitrite, the reduction of nitrite to NO could be rate-limited by either reduction step. By adding the ascorbate to the solution, the [Cu(i)L]^+^ complex could be formed in the electrolyte ahead of the controlled addition of nitrite and the nitrite reduction would be the rate limiting step, preventing the sluggish activity seen in Fig. S10.† Therefore, this poor response was likely due to the buildup of either ascorbate or [Cu(i)L]^+^ that resulted in NO generation long after the infusion was stopped.

### Controlled NO generation by continuous infusion of nitrite

**Fig. 3:**
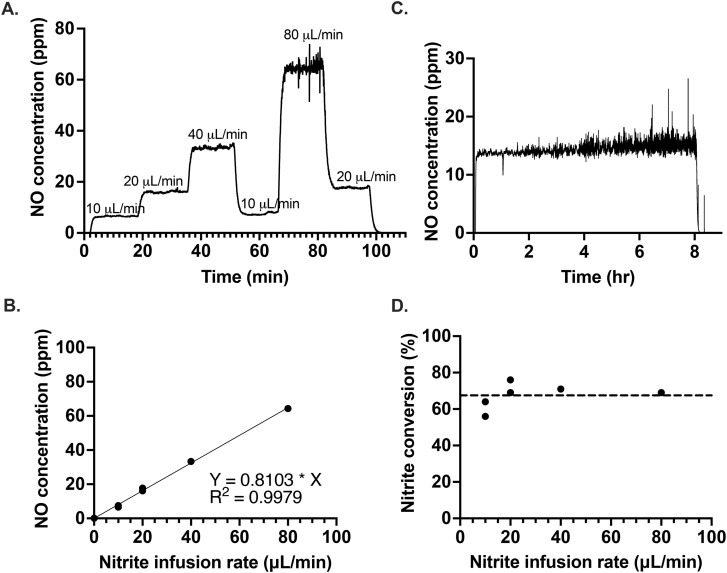
Control of NO generation by the variation of nitrite infusion rate into a solution of [Cu(ii)L]SO_4_ (7 mM) and calcium ascorbate (140 mM); (A) 10–80 μL min^−1^ NaNO_2_, (B) correlation between the nitrite infusion rate and the concentration of NO generated, (C) 7.2 mL of 10 mM NaNO_2_ at 15 μL min^−1^, (D) average (dashed line) conversion as a function of nitrite infusion rate.

The excessive dilution of the electrolyte caused by the continuous infusion of nitrite ions can be easily abated by infusing a high concentration of NaNO_2_, due to the high solubility of NaNO_2_. To demonstrate the responsiveness of the optimized system, we varied the rate of nitrite infusion to the electrolyte as shown in Fig. 3A. After a change in the infusion rate, the new NO concentration steady state was reached within a couple of minutes, regardless of whether the infusion rate was being increased or decreased. In the future, this rapid response rate would allow for precise feedback control in obtaining the desired NO concentration levels, even in situations where that required amount is variable. The amount of NO generated was proportional with the flow rate of nitrite infused (Fig. 3B). It was also possible to generate a controlled and stable amount of NO over several hours of NaNO_2_ infusion, as illustrated in Fig. 3C. The average conversion rate of nitrite to NO was 67.5(±6.8)% (Fig. 3D).

In Fig. 3C, there is an observable increase in the amount of noise of NO concentration with time. The source of the noise was identified as an artifact of the reaction cell geometry and correlates with the increasing liquid level over time, as demonstrated in Fig. S11.† In Fig. 3C, the slight upward slope observed may be a result of the electrolyte aging, the further effects of which are discussed below.

### Storage stability of the electrolyte

**Fig. 4:**
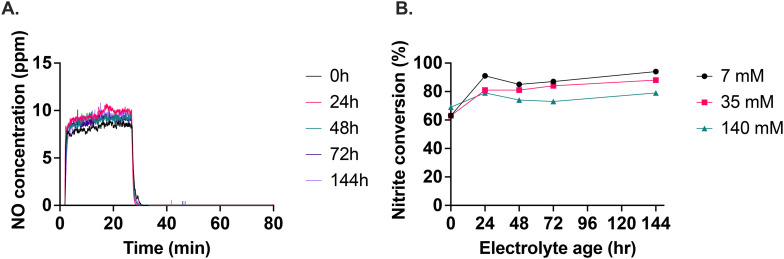
Effect of storage on the optimized electrolyte (A). A total of 250 μL of 10 mM NaNO_2_ was infused at 10 μL min^−1^ into electrolyte stored over time. (B) Nitrite conversion to NO as a function of calcium ascorbate concentration over six days.

We found that the electrolyte with 140 mM calcium ascorbate was the most consistent in terms of the level of NO generated over storage time (Fig. 4A). The 7 mM and 35 mM calcium ascorbate concentrations produced inconsistent levels of NO as storage time progressed (Fig. S12†). For the 140 mM calcium ascorbate, the plateau of NO generation was reached faster after starting the nitrite infusion and also returned to baseline faster after nitrite was stopped, demonstrating consistent responsiveness at this calcium ascorbate level. The conversion of nitrite to NO ranged between 60% to 90%, depending on the age of the electrolyte and the calcium ascorbate concentration (Fig. 4B). 140 mM calcium ascorbate concentration was the most stable of the concentrations tested.

### The role of oxalate as an ascorbate decomposition product on NO generation

Of note, all three calcium ascorbate concentrations shared the characteristic that the lowest nitrite to NO conversion was observed when the electrolyte was freshly made. This behavior seems contradictory to our earlier finding that oxalate interferes with the NO generation, and this warrants further investigation.

**Fig. 5:**
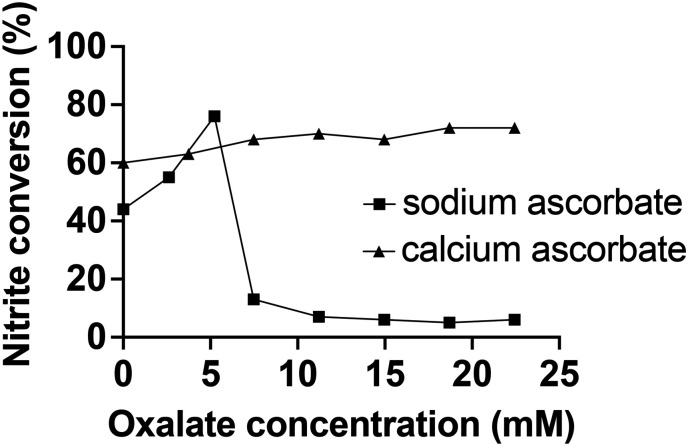
The effect of oxalate concentration on nitrite conversion to NO. 15 μL min^−1^ of nitrite was infused to 5 mL of electrolyte containing either 280 mM sodium ascorbate or 140 mM calcium ascorbate. The titration was performed by bolus injections of 100 μL of 187 mM sodium oxalate in MES pH 5.8 into the electrolyte.

We titrated the electrolyte system with oxalate in the presence and absence of Ca^2+^ (Fig. 5).

In accordance with our findings described earlier, we found that in the presence of Ca^2+^ the oxalate did not have a detrimental effect on the nitrite conversion (Fig. 5). Also, we found that the addition of Ca^2+^ ions was able to restore NO generation from extensively stored electrolyte (Fig. S13†). Interestingly, while it was previously observed that oxalate at higher concentrations ultimately ceases the NO generation (Fig. S5†), there was an unexpected initial increase in the conversion rate at low oxalate concentrations without calcium ions (Fig. 5). There is also an unaccounted mass balance deficit of nitrite. It was demonstrated that the NO generation is precisely controlled by the nitrite infusion (Fig. 3A) when there is an excess of reducing agent present, without the buildup of nitrite in the electrolyte and subsequent sluggishness. Therefore, the imperfect conversion ratio cannot be explained by an inadequate amount of [Cu(i)L]^+^ present.

One possible explanation for both the initial increase of NO generation in the presence of oxalate and the unaccounted mass balance deficit is that [Cu(i)L]^+^ may be capable of the further reduction of NO to N_2_O. It is known that nitrite reductase enzymes with copper active sites are capable of reducing nitrite a step further to N_2_O.^37^ The proposed mechanism for this reduction involves the formation of a [Cu(i)L]NO_2_ complex, followed by dehydration to form a [Cu(i)LNO]^2+^ complex that can then either produce NO, or react with an additional NO molecule or nitrite ion to form N_2_O. Importantly, oxalate can form complexes with copper.^33^ If oxalate has a higher binding affinity for the copper centers than the nitrosyl but a lower binding affinity than nitrite, it could also explain why there was an increase in nitrite conversion at small concentrations of oxalate (Fig. 5). We confirmed the presence of N_2_O in the generated gas using FTIR (Fig. S14†), that may account, at least in part, for the mass balance deficit of NO formation from nitrite reduction.

The above findings suggest that oxalate affects the amount of NO generated *via* [Cu(ii)L]^2+^ mediated chemistry, likely through a combination of multiple mechanisms: oxalate can stabilize the ascorbate, preventing it from reducing the [Cu(ii)L]^2+^; oxalate can form a complex with [Cu(ii)L]^2+^,^33^ potentially limiting the binding of nitrite to the copper center at high oxalate concentrations; and through an observed interaction of oxalate with NO that results in the interference of the detection of NO (Fig. S15†).

### Other reducing agents

**Fig. 6:**
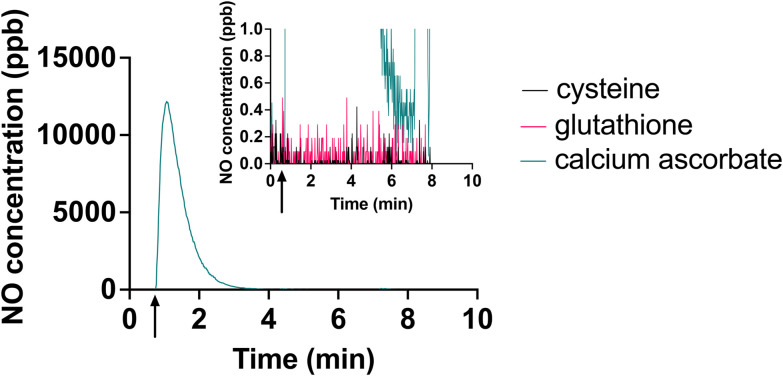
Comparison of NO generation using 140 mM of cysteine, glutathione and ascorbate as the reducing agent. 2 μL of NaNO_2_ was injected to 5 mL of each electrolyte solution. Arrow on *x*-axis shows the time of injection. Inset shows detail of lower NO concentrations.

Another attempt to circumvent the effects of the oxidation products of ascorbate would be to use a different reducing agent. Given the standard reduction potentials of 190 mV for ascorbate,^38^ −0.245 mV for cysteine,^39^ −0.262 mV for glutathione^39^ and 197 mV for [Cu(ii)L]^2+^,^14^ it is expected that these alternative reducing agents would be capable of reducing the [Cu(ii)L]^2+^ complex. However, in our initial efforts, electrolyte freshly prepared with glutathione or cysteine were not capable of efficient, responsive NO generation from this system (Fig. 6).

We speculate that this may be because cysteine and glutathione form copper-thiolate clusters, and these clusters stabilize copper in the Cu(i) state.^40^ These clusters may also result in steric hindrance, preventing nitrite from reacting with the Cu(i) ions.

## Conclusions

We demonstrated that NO gas can be successfully generated from nitrite *via* [Cu(ii)L]^2+^ mediated ascorbate induced reduction. Oxalate, a degradation product of ascorbate, was found to inhibit this chemistry at higher concentrations, while at low concentrations it may lessen the reduction of nitrite to N_2_O. These inhibitory effects of oxalate were decreased by the removal of oxalate from the solution phase by the formation of the practically insoluble calcium oxalate salt. We proposed possible mechanisms to explain these effects of oxalate. We found that glutathione and cysteine in freshly prepared electrolytes, as replacements for ascorbate as reducing agents, were not capable of inducing the [Cu(ii)L]^2+^ mediated reduction of nitrite to NO.

The NO generation through the optimized method was found to be highly controllable *via* its proportionality to the flow rate of nitrite injected into a reaction chamber containing the reducing components. Hence, this simple method adds to the current collection of innovative methods under development to obviate the high cost and complicated logistics of tank-based iNO delivery systems and could potentially be used for NO generation and delivery in situations where simple mixing of two solutions is ideal.

While much more work needs to be done to optimize the method (*e.g.*, use of higher flow settings that more closely approximate clinical applications and further analysis and optimization of the solution chemistry), the data thus far demonstrate significant promise in providing an approach for future cost-effective, portable and controlled NO generation.

## Author contributions

HN: conceptualization, methodology, validation, investigation, writing, visualization. CF: conceptualization, methodology, investigation, writing, visualization. RB: writing – review & editing, funding acquisition. ZB: conceptualization, validation, investigation, writing – review & editing, resources. SPS: conceptualization, methodology, validation, investigation, writing – review & editing, supervision, project administration, resources, funding acquisition. GL: conceptualization, methodology, validation, investigation, writing – review & editing, supervision, project administration, resources, funding acquisition.

## Data availability

The data supporting this article have been included as part of the ESI.†

## Conflicts of interest

There are no conflicts to declare.

## Supplementary Material

DT-054-D4DT01980F-s001
