# Supplementary material for: Feasibility of controlled nitric oxide generation via ascorbate induced chemical reduction of nitrite ions
Source: Dalton Trans. 2024 Nov 15;54(5):1797–805. doi: 10.1039/d4dt01980f (PMC11579995; doi:10.1039/d4dt01980f)
Supplement: DT-054-D4DT01980F-s001 [file DT-054-D4DT01980F-s001.pdf]

Supplementary Information (SI) for Dalton Transactions.
This journal is © The Royal Society of Chemistry 2024

Supplemental information for

Feasibility of controlled nitric oxide generation via ascorbate induced chemical reduction of nitrite ions

Hannah J. Naldrett,[a] Csilla Fekete,[b] Robert H. Bartlett,[c] Zoltán Benkő,[b,d] Steven P. Schwendeman*[a,e,f] and Gergely Lautner*[a]

[a] *Department of Pharmaceutical Sciences, University of Michigan, Ann Arbor, MI 48109, USA.*
[b] *Department of Inorganic and Analytical Chemistry, Budapest University of Technology and Economics, Budapest, Hungary*
[c] *Department of Surgery, ECLS Laboratory, University of Michigan Medical School, Ann Arbor, MI 48109, USA*
[d] *HUN-REN—BME Computation Driven Chemistry Research Group, Budapest, Hungary*
[e] *Biointerfaces Institute, University of Michigan, Ann Arbor, MI 48109, USA.*
[f] *Department of Biomedical Engineering, University of Michigan, Ann Arbor, MI 48109, USA.*
* Corresponding authors

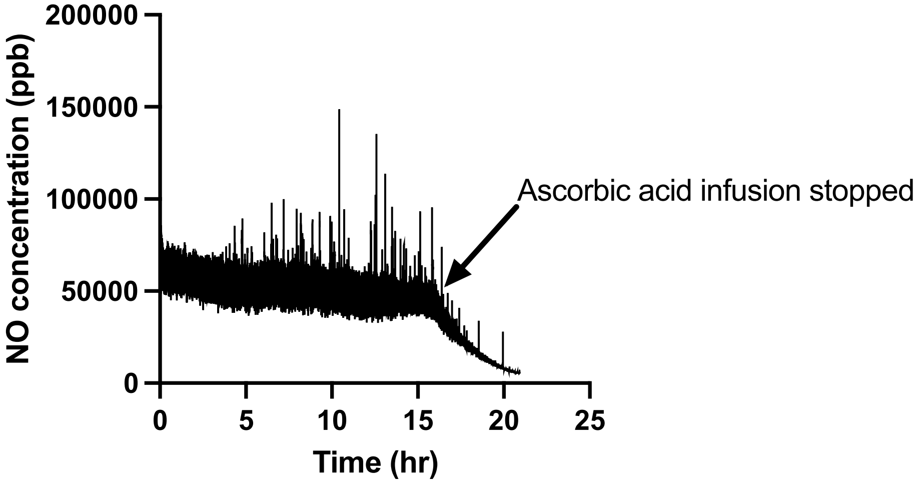

Figure S1. NO generation with ascorbic acid infused into electrolyte is sluggish. Infusion of 1 mL of 0.28 M ascorbic acid infused at 1 μL/min into 5 mL of electrolyte containing 1 M $NaNO_2$.

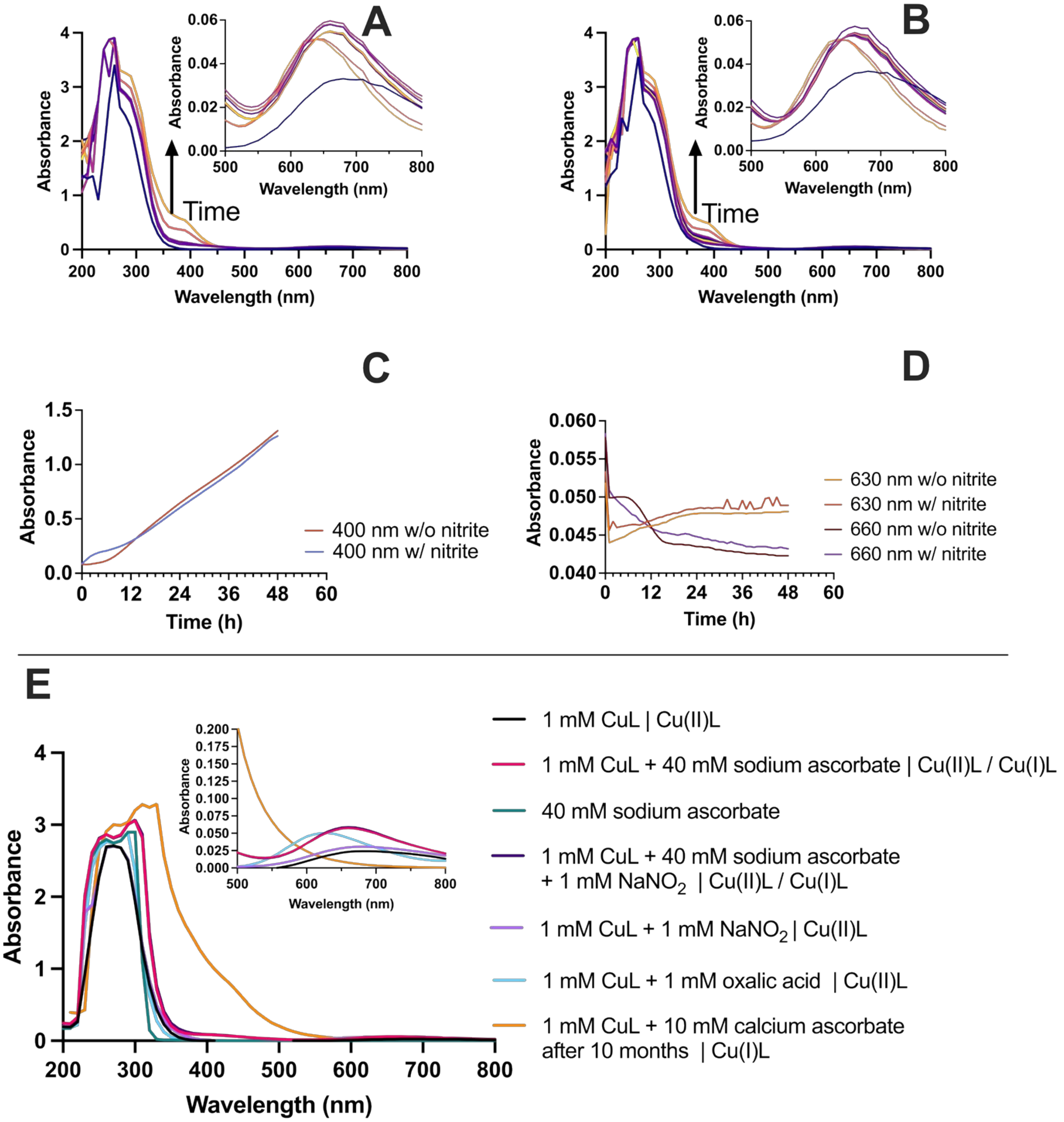

Figure S2. UV-vis spectra of deoxygenated electrolyte before and after addition of ascorbate over time (0 min, 10 min, 20 min, 30 min, 1 h, 2 h, 3 h, 4 h, 24 h, 48 h) (A) in the absence and (B) presence of nitrite ions. Insets show the visible (500-800 nm) range. Absorbance change of the electrolyte at (C) 400 nm and (D) 630 nm and 660 nm with and without nitrite. (E) UV-vis spectra of different electrolyte compositions in 0.5 M MES pH 5.8, exposed to air. Legend shows the individual components of electrolyte, where CuL represents Cu(II)L; the composition may change after mixing the components, as noted.

Table S1. The structures of the investigated complexes at ωB97X-D/def2SVP level.

Name	Structure
**$[Cu(I)L]^+$**<br>Selected wavelengths:<br>376.14 nm (f = 0.01)<br>228.44 nm (f = 0.01)<br>228.39 nm (f = 0.01)<br>216.66 nm (f = 0.04)	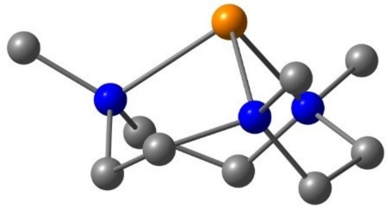
**$[Cu(I)L(H_2O)]^+$**<br>Selected wavelengths:<br>207.38 nm (f = 0.01)<br>202.54 nm (f = 0.01)<br>200.37 nm (f = 0.01)<br>199.78 nm (f = 0.01)<br>198.94 nm (f = 0.01)	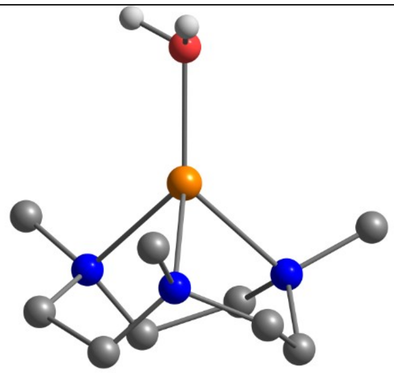
**$[Cu(I)LHAsc]^+$**<br>Selected wavelengths:<br>344.97 nm (f = 0.01)<br>303.59 nm (f = 0.05)<br>274.26 nm (f = 0.03)<br>258.64 nm (f = 0.01)<br>235.47 nm (f = 0.04)	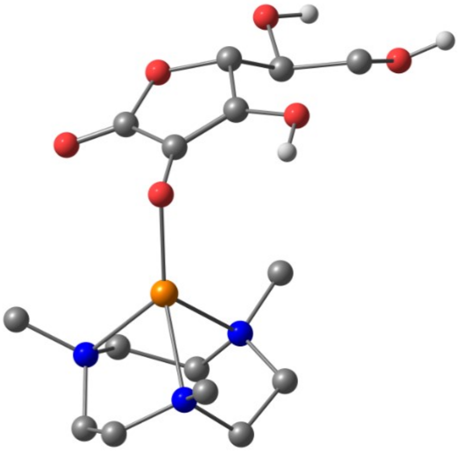

**[Cu(I)LDHA]⁺**<br>Selected wavelengths:<br>828.59 nm (f = 0.01)<br>686.54 nm (f = 0.15)<br>566.75 nm (f = 0.06)<br>402.51 nm (f = 0.01)<br>367.25 nm (f = 0.02)<br>318.00 nm (f = 0.01)<br>299.75 nm (f = 0.02)<br>279.60 nm (f = 0.01)<br>271.87 nm (f = 0.06)	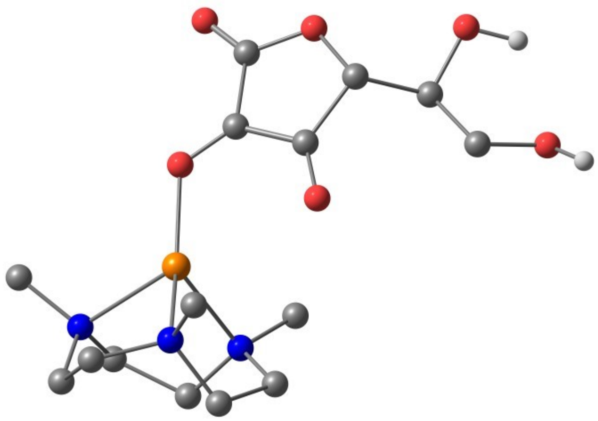
**[Cu(II)L]²⁺**<br>Selected wavelengths:<br>529.57 nm (f = 0.03)<br>425.46 nm (f = 0.06)<br>347.12 nm (f = 0.02)<br>304.90 nm (f = 0.01)<br>295.01 nm (f = 0.01)<br>282.31 nm (f = 0.01)<br>280.25 nm (f = 0.01)<br>229.70 nm (f = 0.01)<br>222.94 nm (f = 0.01)<br>229.70 nm (f = 0.01)<br>213.74 nm (f = 0.04)<br>212.48 nm (f = 0.01)<br>202.02 nm (f = 0.01)	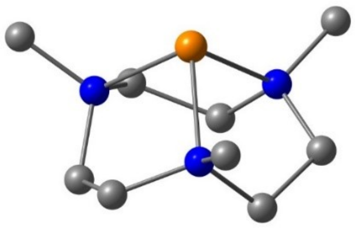

**$[Cu(II)L(H_2O)]^{2+}$**<br>Selected wavelengths:<br>439.70 nm (f = 0.06)<br>407.11 nm (f = 0.03)<br>335.46 nm (f = 0.04)<br>260.56 nm (f = 0.01)<br>258.23 nm (f = 0.01)<br>257.29 nm (f = 0.01)<br>211.92 nm (f = 0.01)<br>204.42 nm (f = 0.01)<br>176.54 nm (f = 0.02)	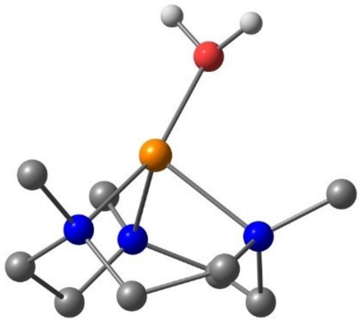
**$[Cu(II)L(H_2O)_2]^{2+}$**<br>Selected wavelengths:<br>432.28 nm (f = 0.01)<br>368.88 nm (f = 0.07)<br>319.14 nm (f = 0.07)<br>244.88 nm (f = 0.01)<br>240.91 nm (f = 0.01)<br>233.5 nm (f = 0.01)<br>193.64 nm (f = 0.01)<br>189.86 nm (f = 0.01)<br>176.29 nm (f = 0.01)	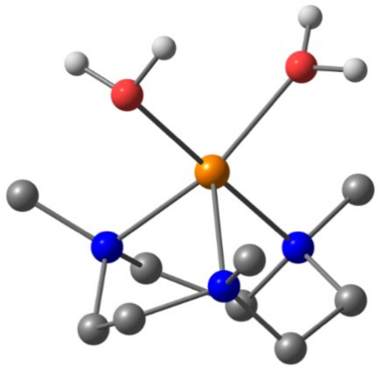
**$[Cu(II)L(H_2O)_3]^{2+}$**<br>Selected wavelengths:<br>348.80 nm (f = 0.09)<br>310.91 nm (f = 0.08)<br>221.37 nm (f = 0.01)<br>214.29 nm (f = 0.01)	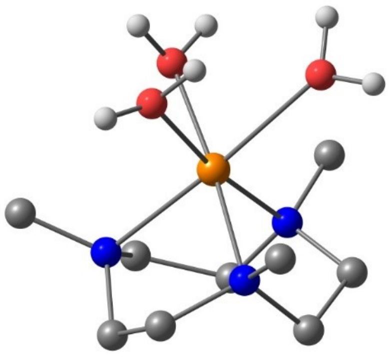

**[Cu(II)LAsc], conformer 1**<br>Selected wavelengths:<br>745.35 nm (f = 0.01)<br>656.06 nm (f = 0.05)<br>340.35 nm (f = 0.01)<br>317.40 nm (f = 0.03)<br>262.51 nm (f = 0.01)<br>260.31 nm (f = 0.01)	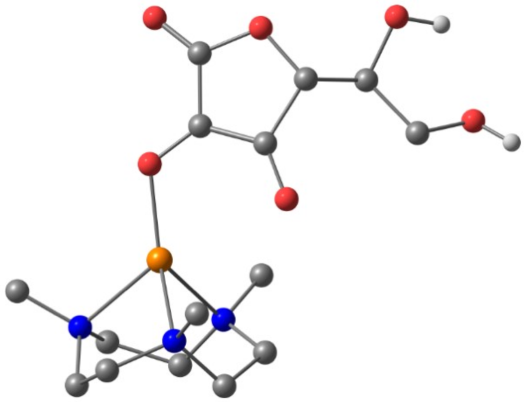
**[Cu(II)LAsc], conformer 2**<br>Selected wavelengths:<br>595.80 nm (f = 0.01)<br>365.97 nm (f = 0.07)<br>298.34 nm (f = 0.01)<br>285.00 nm (f = 0.01)<br>272.68 nm (f = 0.08)<br>268.98 nm (f = 0.04)<br>261.93 nm (f = 0.01)	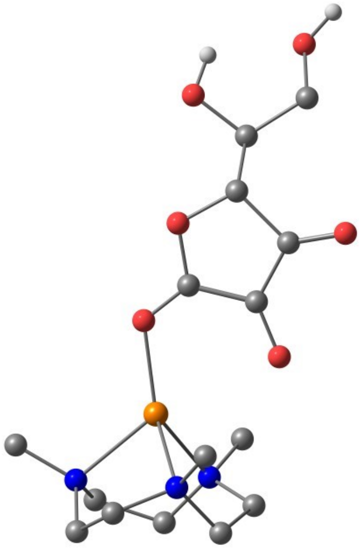
**[Cu(II)LAsc], conformer 3**<br>Selected wavelengths:<br>676.81 nm (f = 0.01)<br>468.92 nm (f = 0.07)<br>382.59 nm (f = 0.01)<br>378.17 nm (f = 0.07)<br>345.49 nm (f = 0.01)<br>294.52 nm (f = 0.01)<br>267.27 nm (f = 0.04)	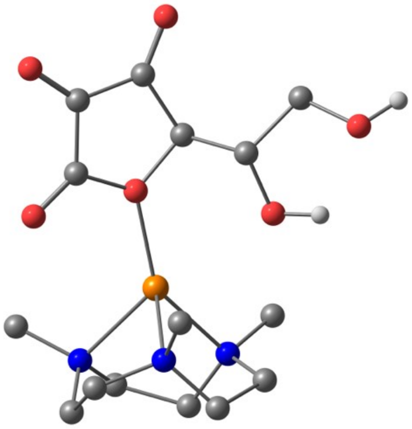

**$[Cu(II)LHAsc]^{+}$**<br>Selected wavelengths:<br>650.41 nm (f = 0.08)<br>617.40 nm (f = 0.10)<br>372.38 nm (f = 0.01)<br>365.17 nm (f = 0.02)<br>324.70 nm (f = 0.03)<br>309.37 nm (f = 0.03)<br>280.62 nm (f = 0.02)<br>235.81 nm (f = 0.19)<br>226.49 nm (f = 0.02)<br>215.51 nm (f = 0.01)	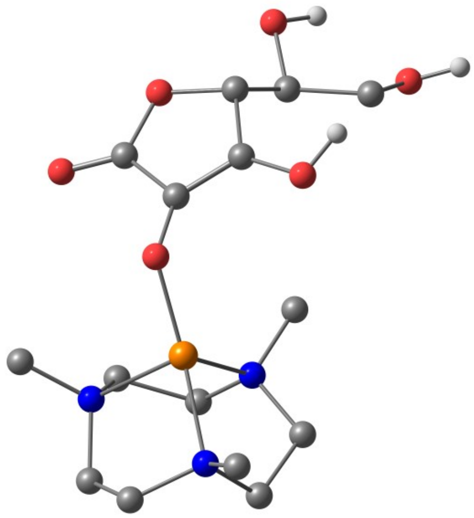
**$[Cu(II)LDHA]^{2+}$**<br>Selected wavelengths:<br>895.07 nm (f = 0.02)<br>441.38 nm (f = 0.03)<br>414.81 nm (f = 0.01)<br>381.71 nm (f = 0.01)<br>374.04 nm (f = 0.01)<br>366.14 nm (f = 0.04)<br>342.66 nm (f = 0.04)<br>335.37 nm (f = 0.08)	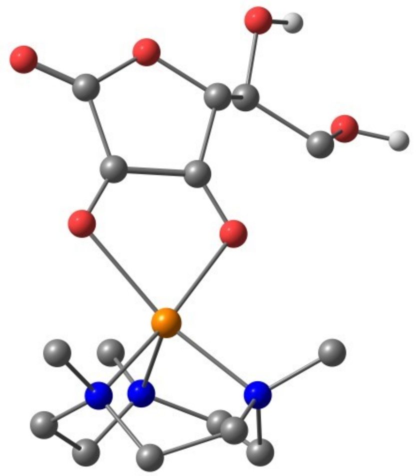

**[Cu(I)LNO$_2$], conformer 1**<br>Selected wavelengths:<br>349.13 nm (f = 0.05)<br>231.61 nm (f = 0.01)<br>230.53 nm (f = 0.01)<br>223.11 nm (f = 0.01)<br>222.95 nm (f = 0.01)<br>219.04 nm (f = 0.01)<br>204.38 nm (f = 0.01)<br>203.65 nm (f = 0.01)<br>199.87 nm (f = 0.02)	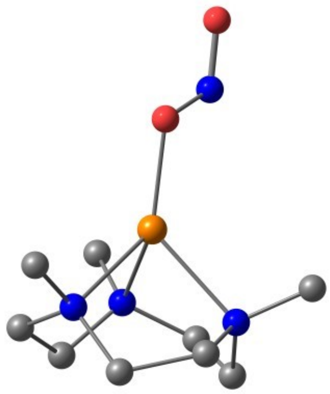
**[Cu(I)LNO$_2$], conformer 2**<br>Selected wavelengths:<br>391.78 nm (f = 0.01)<br>300.27 nm (f = 0.05)<br>286.20 nm (f = 0.02)<br>273.82 nm (f = 0.01)<br>268.92 nm (f = 0.01)<br>263.55 nm (f = 0.01)<br>227.21 nm (f = 0.01)<br>205.44 nm (f = 0.01)<br>197.05 nm (f = 0.01)<br>193.49 nm (f = 0.01)<br>188.52 nm (f = 0.01)<br>186.66 nm (f = 0.01)	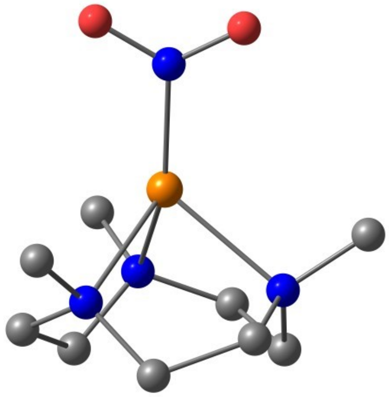

**$[Cu(II)LNO_2]^+$, conformer 1**<br>Selected wavelengths:<br>837.18 nm (f = 0.02)<br>387.70 nm (f = 0.11)<br>363.02 nm (f = 0.03)<br>361.24 nm (f = 0.02)<br>360.00 nm (f = 0.03)<br>333.54 nm (f = 0.01)<br>255.88 nm (f = 0.01)<br>222.77 nm (f = 0.01)<br>220.82 nm (f = 0.01)	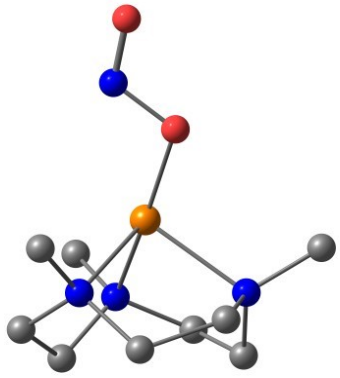
**$[Cu(II)LNO_2]^+$, conformer 2**<br>Selected wavelengths:<br>562.20 nm (f = 0.01)<br>542.95 nm (f = 0.01)<br>430.29 nm (f = 0.11)<br>356.42 nm (f = 0.01)<br>341.30 nm (f = 0.03)<br>330.81 nm (f = 0.01)<br>309.31 nm (f = 0.01)<br>305.02 nm (f = 0.02)<br>302.61 nm (f = 0.02)<br>259.66 nm (f = 0.01)<br>257.34 nm (f = 0.01)<br>208.38 nm (f = 0.01)	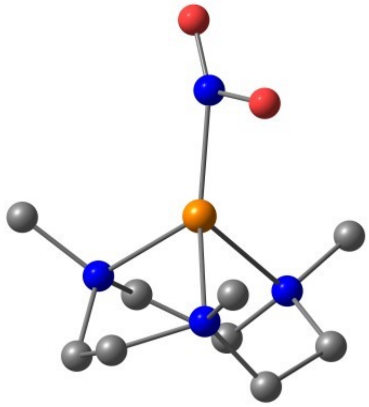

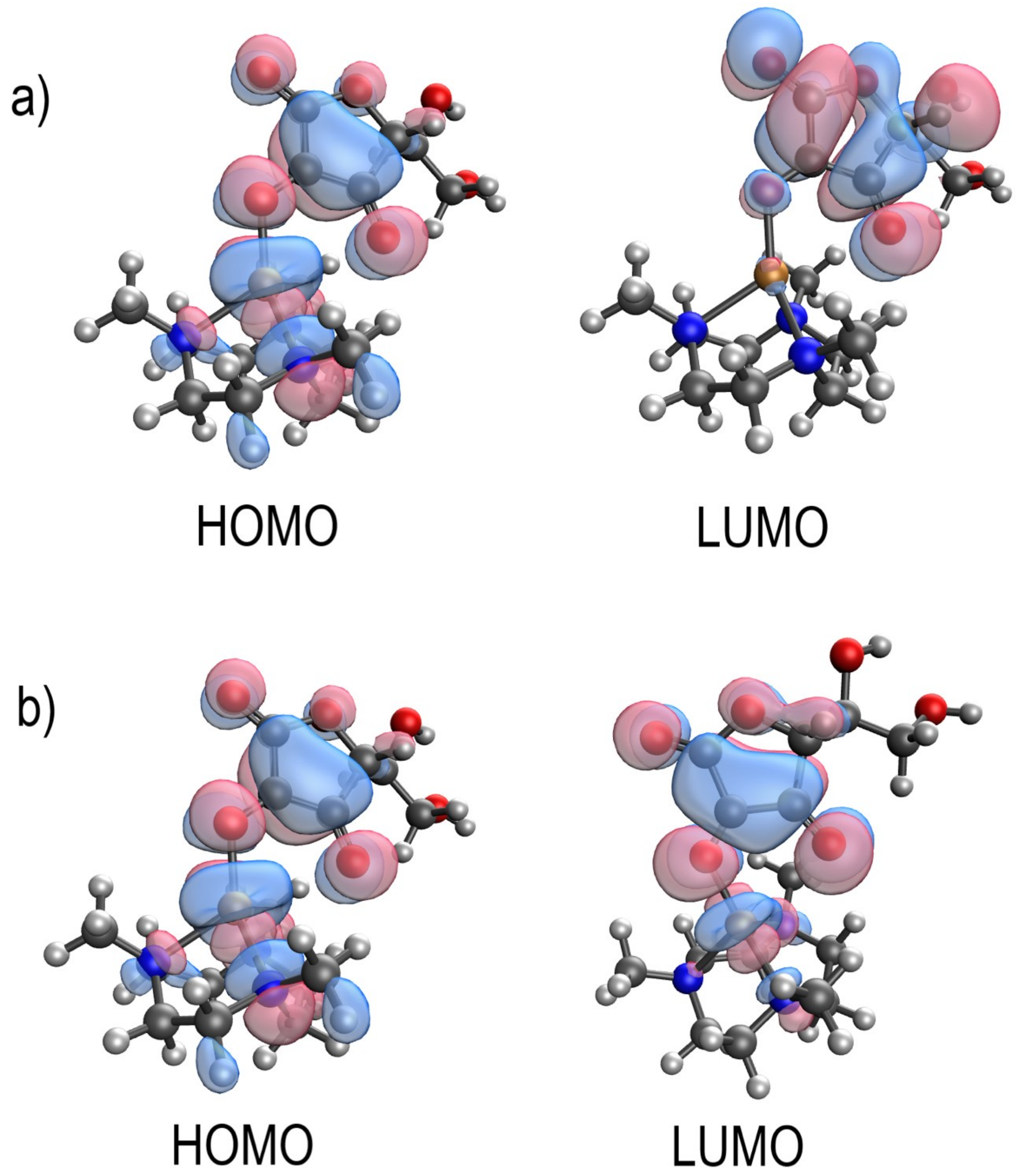

Figure S3. The HOMO and the LUMO of the a) [Cu(II)L(Asc)], conformer 1, b) $[Cu(I)L(DHA)]^{+}$ at ωB97X-D/def2SVP level, isovalue = 0.050.

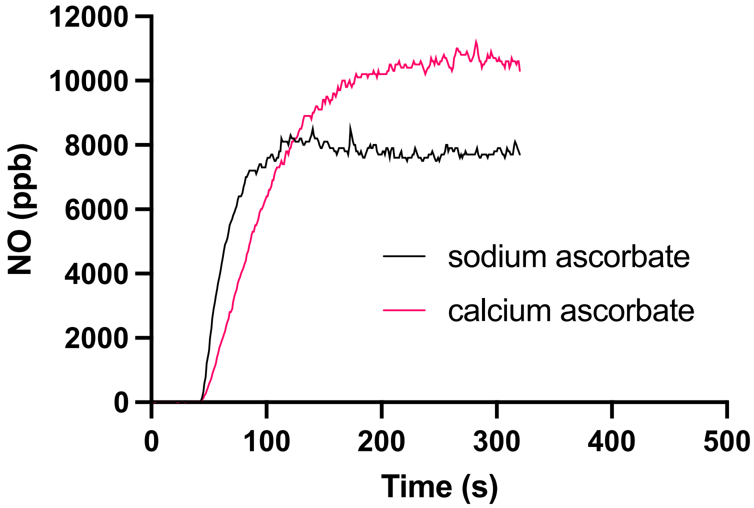

Figure S4: NO generation of electrolyte containing sodium ascorbate (280 mM) and calcium ascorbate (140 mM) infused with 15 μL/min 10 mM sodium nitrite).

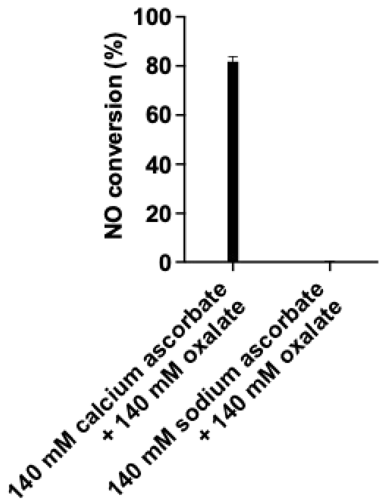

Figure S5. Oxalate present at a high initial concentration in the electrolyte stops nitrite conversion. The presence of 140 mM sodium oxalate dissolved into solution along with 140 mM sodium ascorbate prevents any nitrite conversion to NO from the bolus injection of 2 μL of 10 mM sodium nitrite, as compared to the control with no added sodium oxalate.

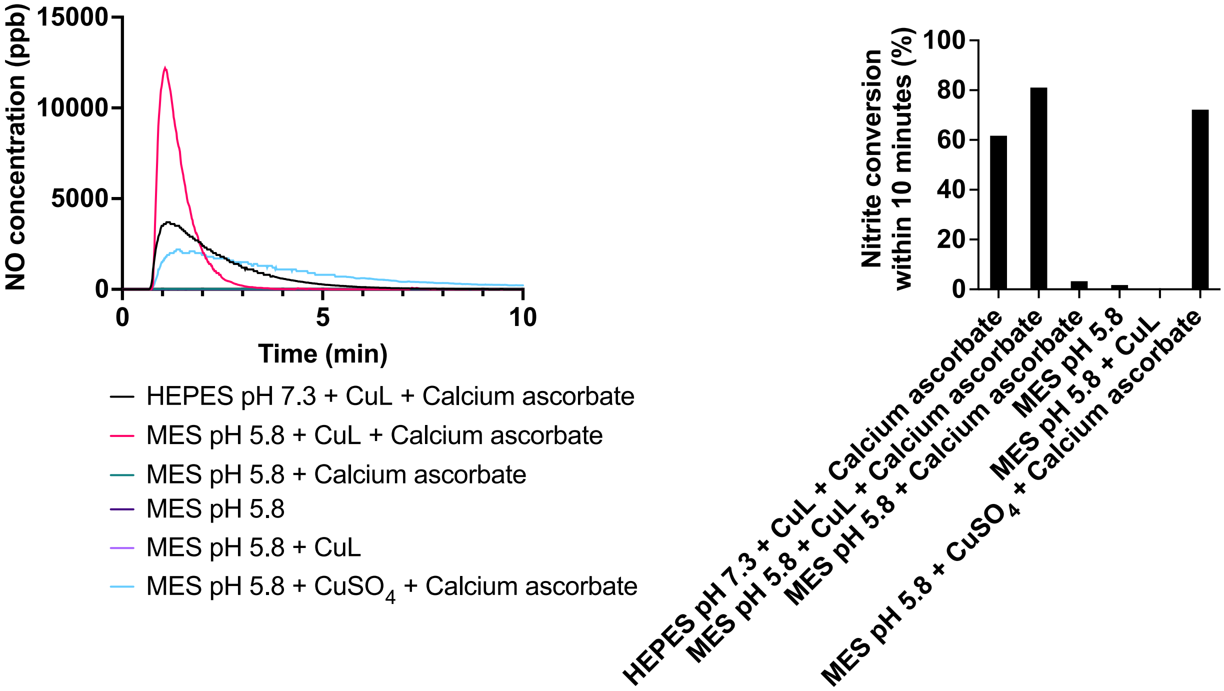

Figure S6. Generated NO concentrations (left) and nitrite conversion to NO (right) from different electrolyte compositions. 10 μL of 10 mM sodium nitrite was injected into 5 mL of the specified electrolyte composition (when present, concentrations of the components are 7 mM CuL, 140 mM calcium ascorbate, and 7 mM copper sulfate).

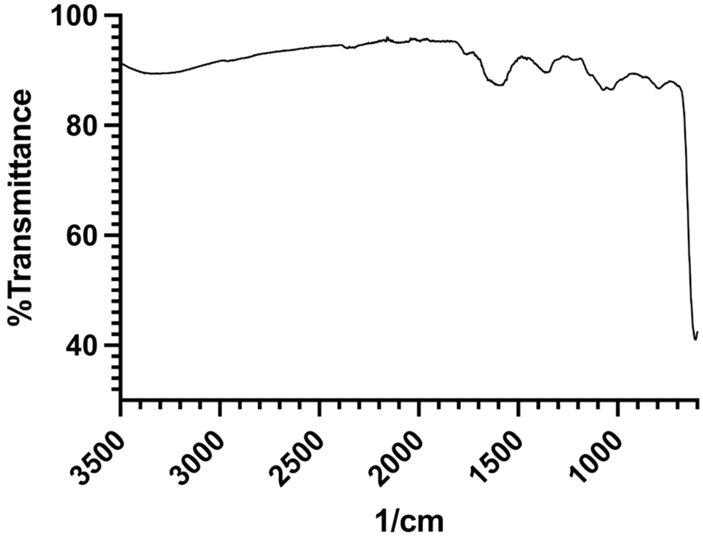

Figure S7. FT-IR ATR spectrum of the freeze dried yellow precipitate formed after mixing $CuSO_4$ and calcium ascorbate.

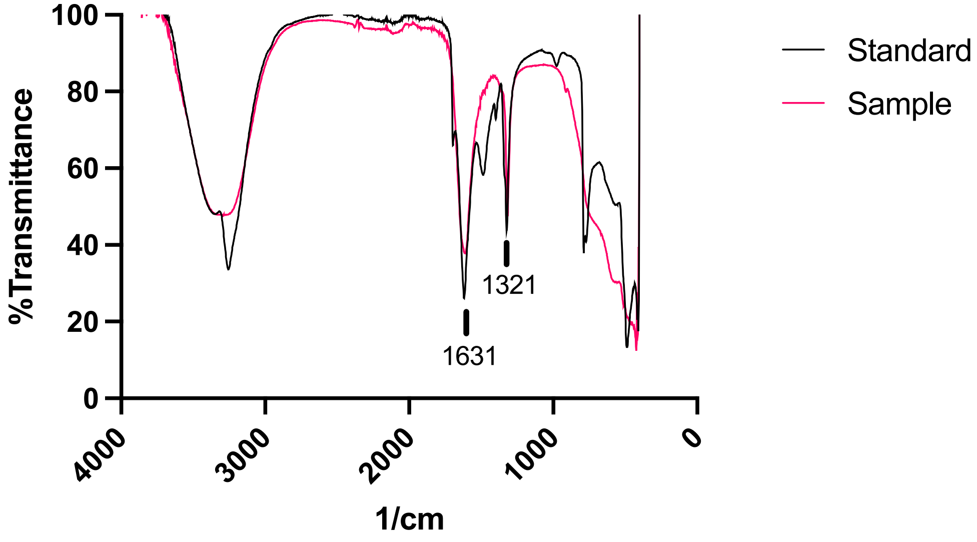

Figure S8. FT-IR ATR spectra of calcium oxalate standard (Standard) and the precipitate (Sample) formed with Ca^{2+} during the storage of the electrolyte. The asymmetric stretching (v_a), symmetric stretching (v_s) oxalate fundamental vibrations assigned to the bands at 1631 and 1321 cm^{-1}, respectively.

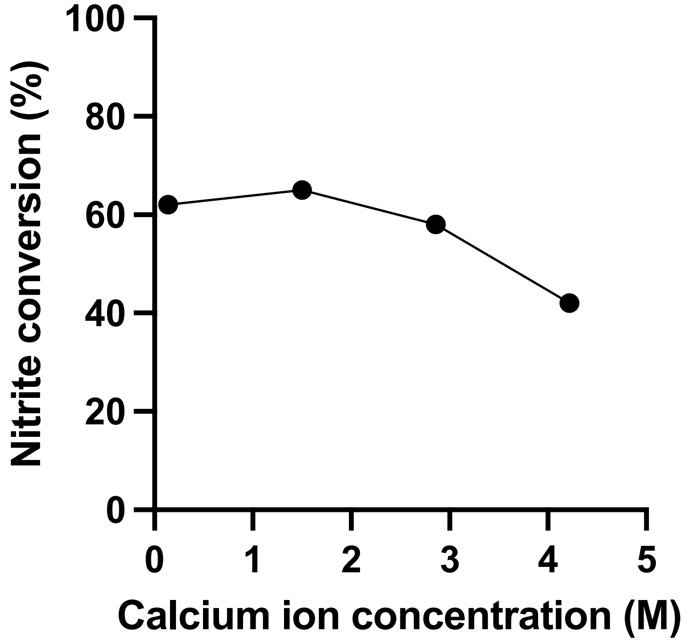

Figure S9. Very high concentrations of calcium ions will eventually deplete nitrite conversion to NO. 15 μL/min of nitrite was infused to 5 mL of electrolyte containing 140 mM calcium ascorbate. One gram of calcium chloride was added by removing one mL of electrolyte from the reaction cell and dissolving the calcium chloride in it, then adding it back to the reaction cell. This was done once a plateau from the previous addition was reached.

Table S2. Summary of electrolyte optimization process.

**Initial electrolyte components in reaction chamber**	**NO generating addition**	**Major issue(s)**	**Expected source of the issues**	**Improvement**
TACN, $CuSO_4$, nitrite, HEPES pH 7.4	Ascorbic acid	Slow return to baseline	Cu(I) accumulation	
TACN, $CuSO_4$, sodium ascorbate, HEPES pH 7.4	Nitrite	Short shelf life, slow and incomplete NO conversion	Oxalate accumulation due to high pH	Infusing Nitrite greatly improved return to baseline
TACN, $CuSO_4$, sodium ascorbate, MES pH 5.8	Nitrite	Short shelf life	Oxalate accumulation	Optimizing pH improved response time and conversion rate without proton mechanism
TACN, $CuSO_4$, calcium ascorbate, MES pH 5.8	Nitrite			Added calcium ions to remove oxalate from solution
TACN, $CuSO_4$, glutathione, MES pH 5.8	Nitrite	Slow and incomplete NO conversion	Thiolate cluster formation	None
TACN, $CuSO_4$, cysteine, MES pH 5.8	Nitrite	Slow and incomplete NO conversion	Thiolate cluster formation	None
$CuSO_4$, calcium ascorbate, MES pH 5.8	Nitrite	NO was not produced	Copper ions precipitate	None

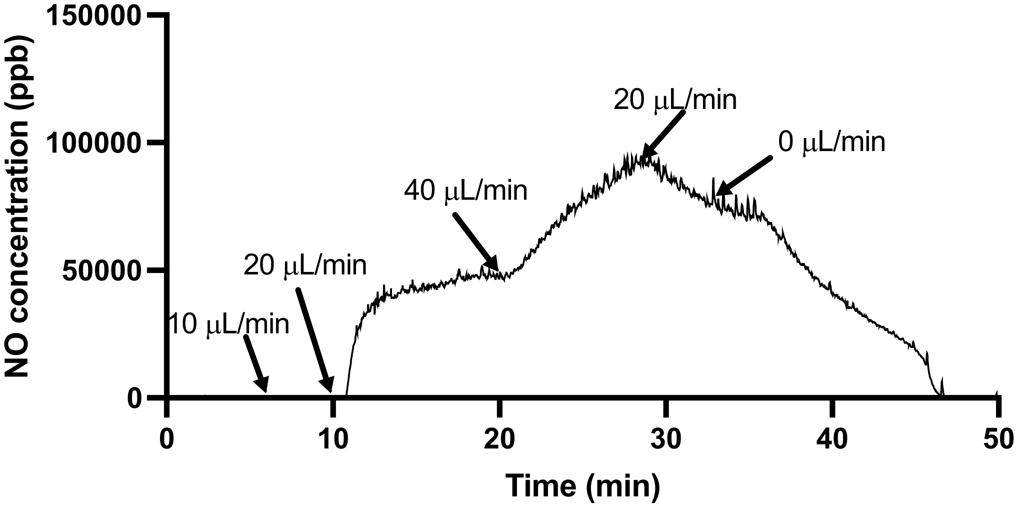

Figure S10. 10 mM calcium ascorbate infused into optimized electrolyte and 140 mM sodium nitrite results in sluggish NO generation and slow responsiveness.

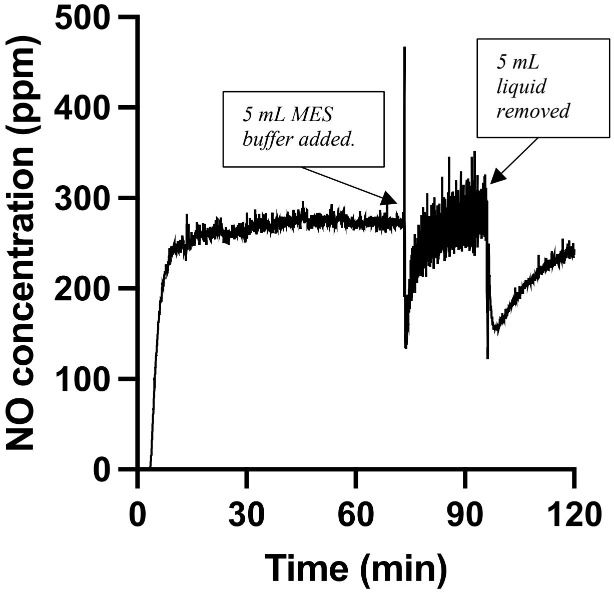

Figure S11. Noisier signal from increased water column. Nitrite (242 mM) was infused at 15 μL/min. There is a part of the reaction cell where the needle is inserted via a septum that, as the water level rises within the cell, can trap NO until sufficient pressure builds up to release the NO. This results in higher spikes and lower dips as more pressure is needed to release the bubble. Additionally, there is likely some variation in the size of the N_2 bubbles sparging the solution over time as a result of the increasing reaction chamber volume. This effect could have been responsible for the increase in NO signal noise observed with time.

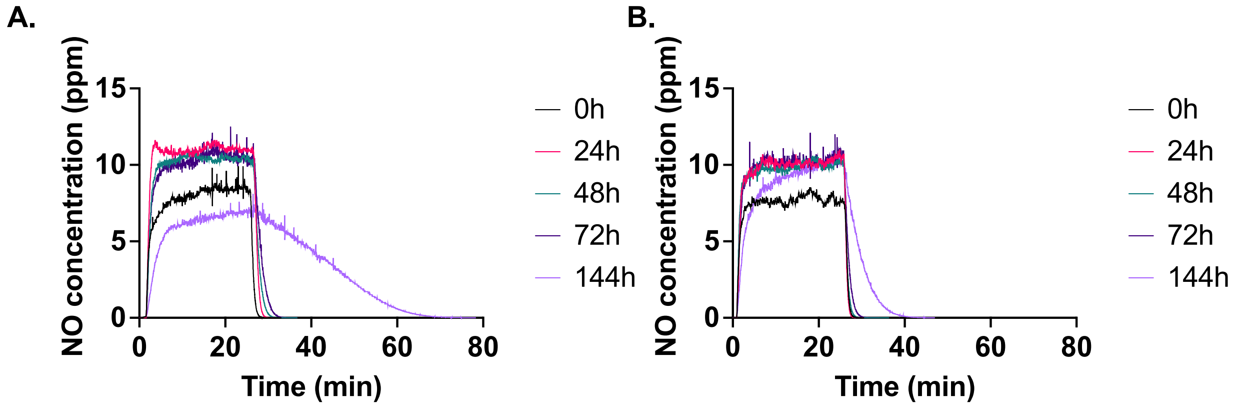

Figure S12: Storage stability of TACN-copper complex with calcium ascorbate concentration of 7 mM (A) and 35 mM (B) over six days. A total of 250 μL of 10 mM $NaNO_2$ was infused at 10 μL/min. The N_2 sparging rate was 0.15 L/min.

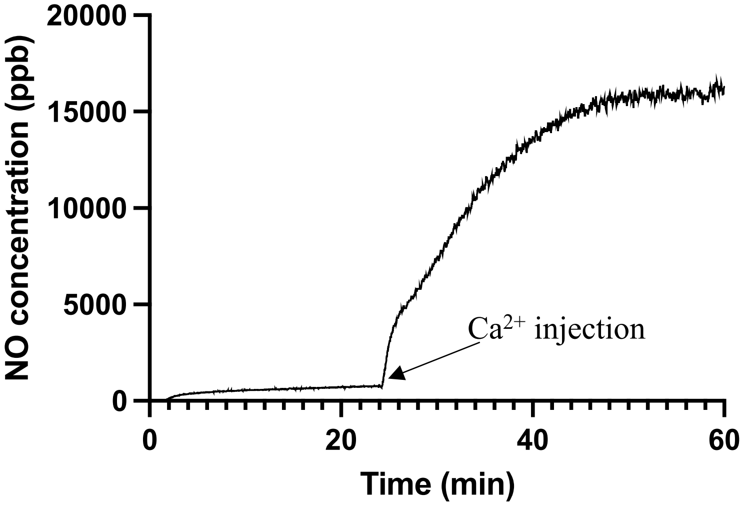

Figure S13: NO generation from continuous nitrite infusion (15 μL/min 10 mM) into electrolyte (1 mL, 1 M ascorbate, MES pH 5.8, 7 mM CuL) stored for 9 months. Electrolyte was stored at RT without light protection, without deoxygenation and without calcium ions. NO generation (flow rate: 200 mL/min) was restored with the addition of calcium ions (100 mL 192 mM $CaCl_2$).

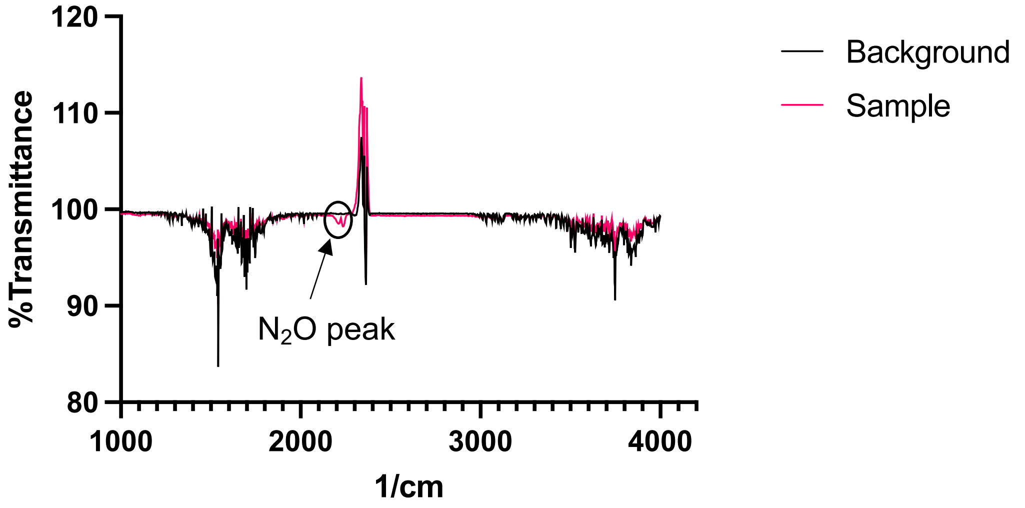

Figure S14. FT-IR spectra of the generated gas from the reaction cell containing 25 mL of 7 mM $[Cu(II)L]^{2+}$, 3.5 mM calcium ascorbate and 14 mM $CaCl_2$ before (black) and after (pink) the addition of 1 mL of 10 mM $NaNO_2$.

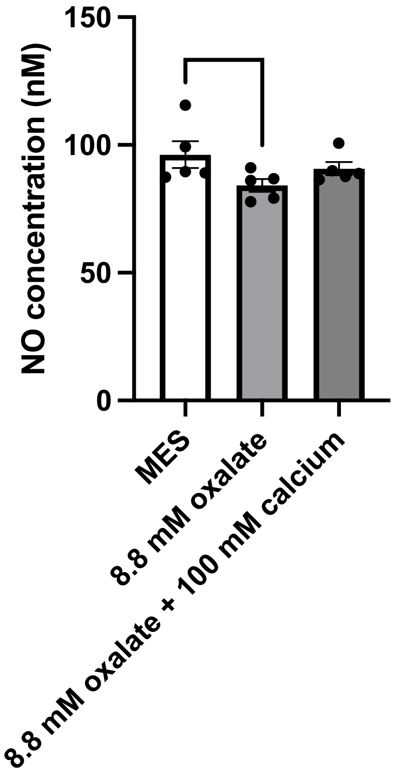

Figure S15. The presence of oxalate in a buffered solution scavenges injected NO gas. 10 mL of DI water was deoxygenated by vigorous sparging with N_2 gas, then 50 ppm of 50 mL/min NO(g) in N_2 was sparged through this deoxygenated water. 200 μL of the NO solution was injected through a Hamilton syringe into a glass reaction cell containing 5 mL of the specified buffered salts.

Time dependent DFT calculations (at B3LYP/def2SVP//ωB97X-D/def2SVP level)

[Cu(I)L]$^+$

```
Excitation energies and oscillator strengths:

Excited State   1:      Singlet-A      2.4449 eV  507.11 nm  f=0.0002  <S**2>=0.000
     62 -> 63         0.69792
This state for optimization and/or second-order correction.
Total Energy, E(TD-HF/TD-DFT) =  -2159.51927131
Copying the excited state density for this state as the 1-particle RhoCI density.

Excited State   2:      Singlet-A      2.4455 eV  506.99 nm  f=0.0002  <S**2>=0.000
     61 -> 63         0.69792

Excited State   3:      Singlet-A      3.1802 eV  389.86 nm  f=0.0018  <S**2>=0.000
     60 -> 63         0.70034

Excited State   4:      Singlet-A      3.1804 eV  389.84 nm  f=0.0018  <S**2>=0.000
     59 -> 63         0.70034

Excited State   5:      Singlet-A      3.2962 eV  376.14 nm  f=0.0062  <S**2>=0.000
     58 -> 63         0.70307

Excited State   6:      Singlet-A      5.4275 eV  228.44 nm  f=0.0110  <S**2>=0.000
     56 -> 63         0.68167

Excited State   7:      Singlet-A      5.4286 eV  228.39 nm  f=0.0110  <S**2>=0.000
     55 -> 63         0.68162

Excited State   8:      Singlet-A      5.7225 eV  216.66 nm  f=0.0426  <S**2>=0.000
     57 -> 63         0.69094

Excited State   9:      Singlet-A      5.7731 eV  214.76 nm  f=0.0009  <S**2>=0.000
     61 -> 65        -0.32167
     61 -> 66         0.36616
     62 -> 65         0.37680
     62 -> 66         0.32913

Excited State  10:      Singlet-A      5.7916 eV  214.08 nm  f=0.0017  <S**2>=0.000
     61 -> 65         0.28455
     62 -> 64         0.57040
     62 -> 66         0.27330
```

```
Excited State  11:      Singlet-A      5.7929 eV  214.03 nm  f=0.0017  <S**2>=0.000
     61 -> 64         0.56756
     61 -> 66        -0.28963
     62 -> 65         0.27343

Excited State  12:      Singlet-A      5.8163 eV  213.17 nm  f=0.0103  <S**2>=0.000
     61 -> 64        -0.39410
     61 -> 66        -0.40076
     62 -> 65         0.40606

Excited State  13:      Singlet-A      5.8170 eV  213.14 nm  f=0.0103  <S**2>=0.000
     61 -> 65         0.40405
     62 -> 64        -0.39015
     62 -> 66         0.40629

Excited State  14:      Singlet-A      5.8878 eV  210.58 nm  f=0.0025  <S**2>=0.000
     57 -> 63         0.12317
     61 -> 65         0.36700
     61 -> 66         0.32594
     62 -> 65         0.32314
     62 -> 66        -0.36445

Excited State  15:      Singlet-A      6.0909 eV  203.56 nm  f=0.0021  <S**2>=0.000
     62 -> 67         0.70071

Excited State  16:      Singlet-A      6.0915 eV  203.54 nm  f=0.0021  <S**2>=0.000
     61 -> 67         0.70072

Excited State  17:      Singlet-A      6.7342 eV  184.11 nm  f=0.0000  <S**2>=0.000
     60 -> 66         0.39403

Excited State  18:      Singlet-A      6.7715 eV  183.10 nm  f=0.0042  <S**2>=0.000
     59 -> 65        -0.29933
     59 -> 66         0.39518
     60 -> 65        -0.38846
     60 -> 66        -0.29065

Excited State  19:      Singlet-A      6.7795 eV  182.88 nm  f=0.0067  <S**2>=0.000
     59 -> 66         0.47627
     60 -> 65         0.48105

Excited State  20:      Singlet-A      6.7796 eV  182.88 nm  f=0.0068  <S**2>=0.000
     59 -> 65        -0.47805
     60 -> 66         0.48323

Excited State  21:      Singlet-A      6.8107 eV  182.04 nm  f=0.0003  <S**2>=0.000
```

58 -> 65 -0.12487
60 -> 64 0.67932

Excited State 22: Singlet-A 6.8110 eV 182.04 nm f=0.0004 <S**2>=0.000
58 -> 66 0.13262
59 -> 64 0.67535

Excited State 23: Singlet-A 6.8407 eV 181.25 nm f=0.0028 <S**2>=0.000
58 -> 65 0.51764
58 -> 66 -0.42618
59 -> 64 0.13575

Excited State 24: Singlet-A 6.8408 eV 181.24 nm f=0.0027 <S**2>=0.000
58 -> 65 0.42802
58 -> 66 0.51789
60 -> 64 0.13137

Excited State 25: Singlet-A 6.8901 eV 179.94 nm f=0.0002 <S**2>=0.000
58 -> 64 0.69698

Excited State 26: Singlet-A 6.8980 eV 179.74 nm f=0.0000 <S**2>=0.000
62 -> 68 0.69960

Excited State 27: Singlet-A 6.8986 eV 179.72 nm f=0.0000 <S**2>=0.000
61 -> 68 0.69963

Excited State 28: Singlet-A 7.0983 eV 174.67 nm f=0.0002 <S**2>=0.000
60 -> 67 0.69594

Excited State 29: Singlet-A 7.0984 eV 174.66 nm f=0.0002 <S**2>=0.000
59 -> 67 0.69596

Excited State 30: Singlet-A 7.1853 eV 172.55 nm f=0.0042 <S**2>=0.000
61 -> 69 -0.30214
61 -> 70 0.36875
62 -> 69 0.40703
62 -> 70 0.29885

[Cu(I)L(H2O)]$^{+}$

Excitation energies and oscillator strengths:

Excited State 1: Singlet-A 3.2049 eV 386.86 nm f=0.0002 <S**2>=0.000
67 -> 68 0.69927
This state for optimization and/or second-order correction.
Total Energy, E(TD-HF/TD-DFT) = -2235.89219535
Copying the excited state density for this state as the 1-particle RhoCI density.

```
Excited State   2:      Singlet-A      3.2666 eV  379.56 nm  f=0.0000  <S**2>=0.000
     66 -> 68         0.69984

Excited State   3:      Singlet-A      3.9432 eV  314.42 nm  f=0.0000  <S**2>=0.000
     64 -> 68         0.33270
     65 -> 68         0.61562

Excited State   4:      Singlet-A      3.9936 eV  310.46 nm  f=0.0001  <S**2>=0.000
     63 -> 68         0.15547
     64 -> 68         0.60471
     65 -> 68        -0.31655

Excited State   5:      Singlet-A      4.0079 eV  309.35 nm  f=0.0004  <S**2>=0.000
     63 -> 68         0.68075
     64 -> 68        -0.11507
     65 -> 68         0.11478

Excited State   6:      Singlet-A      5.3413 eV  232.12 nm  f=0.0015  <S**2>=0.000
     67 -> 69         0.69536

Excited State   7:      Singlet-A      5.4217 eV  228.68 nm  f=0.0009  <S**2>=0.000
     66 -> 69         0.68342

Excited State   8:      Singlet-A      5.5641 eV  222.83 nm  f=0.0000  <S**2>=0.000
     66 -> 69         0.11245
     66 -> 71         0.11067
     67 -> 70         0.48195
     67 -> 71        -0.46365

Excited State   9:      Singlet-A      5.6142 eV  220.84 nm  f=0.0017  <S**2>=0.000
     66 -> 70        -0.35440
     66 -> 71         0.57620

Excited State  10:      Singlet-A      5.6729 eV  218.56 nm  f=0.0044  <S**2>=0.000
     67 -> 70         0.49154
     67 -> 71         0.49519

Excited State  11:      Singlet-A      5.7204 eV  216.74 nm  f=0.0037  <S**2>=0.000
     66 -> 70         0.60130
     66 -> 71         0.35710

Excited State  12:      Singlet-A      5.8596 eV  211.59 nm  f=0.0012  <S**2>=0.000
     60 -> 68        -0.14550
     62 -> 68         0.33001
     67 -> 71         0.12228
```

```
      67 -> 72         0.50802
      67 -> 74        -0.25606

 Excited State  13:      Singlet-A      5.8974 eV  210.24 nm  f=0.0004  <S**2>=0.000
      60 -> 68         0.16246
      61 -> 68        -0.39306
      65 -> 69         0.21436
      66 -> 71         0.10787
      66 -> 72         0.40278
      66 -> 74        -0.26076

 Excited State  14:      Singlet-A      5.9127 eV  209.69 nm  f=0.0013  <S**2>=0.000
      60 -> 68        -0.18456
      61 -> 68        -0.15236
      62 -> 68         0.45814
      67 -> 72        -0.45307
      67 -> 74        -0.10061

 Excited State  15:      Singlet-A      5.9515 eV  208.32 nm  f=0.0006  <S**2>=0.000
      61 -> 68         0.27810
      65 -> 69        -0.34493
      66 -> 72         0.53172

 Excited State  16:      Singlet-A      5.9785 eV  207.38 nm  f=0.0109  <S**2>=0.000
      60 -> 68        -0.12515
      61 -> 68         0.35069
      65 -> 69         0.56243
      66 -> 72         0.15628

 Excited State  17:      Singlet-A      6.1213 eV  202.54 nm  f=0.0115  <S**2>=0.000
      60 -> 68         0.58125
      61 -> 68         0.17491
      62 -> 68         0.32579

 Excited State  18:      Singlet-A      6.1878 eV  200.37 nm  f=0.0091  <S**2>=0.000
      62 -> 68         0.13959
      63 -> 69        -0.16109
      64 -> 69        -0.13456
      65 -> 70        -0.34880
      65 -> 71         0.50585
      67 -> 74         0.19753

 Excited State  19:      Singlet-A      6.2062 eV  199.78 nm  f=0.0067  <S**2>=0.000
      64 -> 69         0.67170

 Excited State  20:      Singlet-A      6.2322 eV  198.94 nm  f=0.0080  <S**2>=0.000
```

```
      62 -> 68         0.14856
      63 -> 69         0.59100
      67 -> 74         0.30609

 Excited State  21:      Singlet-A      6.2731 eV  197.64 nm  f=0.0029  <S**2>=0.000
      60 -> 68        -0.16787
      63 -> 69        -0.32089
      65 -> 70         0.21819
      65 -> 71        -0.19742
      67 -> 72         0.11473
      67 -> 74         0.47900

 Excited State  22:      Singlet-A      6.3131 eV  196.39 nm  f=0.0040  <S**2>=0.000
      61 -> 68        -0.22930
      66 -> 72         0.15760
      66 -> 74         0.60883

 Excited State  23:      Singlet-A      6.3319 eV  195.81 nm  f=0.0011  <S**2>=0.000
      65 -> 70         0.55296
      65 -> 71         0.42135

 Excited State  24:      Singlet-A      6.4257 eV  192.95 nm  f=0.0046  <S**2>=0.000
      64 -> 70        -0.40417
      64 -> 71         0.53868
      64 -> 74         0.11276

 Excited State  25:      Singlet-A      6.4371 eV  192.61 nm  f=0.0026  <S**2>=0.000
      63 -> 70        -0.37960
      63 -> 71         0.53395
      63 -> 74         0.12150
      67 -> 73        -0.15212

 Excited State  26:      Singlet-A      6.4514 eV  192.18 nm  f=0.0021  <S**2>=0.000
      63 -> 71         0.11704
      67 -> 73         0.68264

 Excited State  27:      Singlet-A      6.5228 eV  190.08 nm  f=0.0017  <S**2>=0.000
      65 -> 72         0.58259
      65 -> 74        -0.15561
      66 -> 73        -0.33223

 Excited State  28:      Singlet-A      6.5386 eV  189.62 nm  f=0.0007  <S**2>=0.000
      65 -> 72         0.33051
      66 -> 73         0.61025

 Excited State  29:      Singlet-A      6.5911 eV  188.11 nm  f=0.0011  <S**2>=0.000
```

64 -> 70 0.54542
64 -> 71 0.42744
64 -> 72 0.11364

Excited State 30: Singlet-A 6.6195 eV 187.30 nm f=0.0000 <S**2>=0.000
63 -> 70 0.55521
63 -> 71 0.41605
63 -> 72 0.11998

$[Cu(I)L(C_6H_7O_6)]^+$

Excitation energies and oscillator strengths:

Excited State 1: Singlet-A 3.5941 eV 344.97 nm f=0.0097 <S**2>=0.000
107 ->109 0.26177
108 ->109 0.62471
This state for optimization and/or second-order correction.
Total Energy, E(TD-HF/TD-DFT) = -2843.36149466
Copying the excited state density for this state as the 1-particle RhoCI density.

Excited State 2: Singlet-A 3.6998 eV 335.11 nm f=0.0008 <S**2>=0.000
107 ->109 0.62167
108 ->109 -0.25788

Excited State 3: Singlet-A 4.0082 eV 309.33 nm f=0.0024 <S**2>=0.000
107 ->110 0.67937
108 ->110 0.11815

Excited State 4: Singlet-A 4.0436 eV 306.62 nm f=0.0020 <S**2>=0.000
106 ->109 0.64736
108 ->110 -0.16534

Excited State 5: Singlet-A 4.0839 eV 303.59 nm f=0.0520 <S**2>=0.000
106 ->109 0.12123
106 ->110 0.19987
107 ->110 -0.11587
108 ->109 -0.11226
108 ->110 0.62745

Excited State 6: Singlet-A 4.3205 eV 286.97 nm f=0.0016 <S**2>=0.000
106 ->111 0.10242
108 ->109 -0.10207
108 ->111 0.66973
108 ->112 -0.13798

Excited State 7: Singlet-A 4.3906 eV 282.38 nm f=0.0032 <S**2>=0.000
108 ->111 0.13755

```
   108 ->112        0.66537

Excited State   8:      Singlet-A      4.4394 eV  279.28 nm  f=0.0015  <S**2>=0.000
   107 ->109       -0.10229
   107 ->111        0.68660

Excited State   9:      Singlet-A      4.5058 eV  275.17 nm  f=0.0022  <S**2>=0.000
   104 ->109       -0.10861
   105 ->109        0.13773
   107 ->112        0.65790

Excited State  10:      Singlet-A      4.5208 eV  274.26 nm  f=0.0278  <S**2>=0.000
   104 ->109        0.10916
   105 ->109        0.11498
   106 ->110        0.63676
   108 ->110       -0.15170

Excited State  11:      Singlet-A      4.5378 eV  273.23 nm  f=0.0025  <S**2>=0.000
   103 ->109        0.11218
   104 ->109       -0.28043
   105 ->109        0.56396
   107 ->112       -0.18388

Excited State  12:      Singlet-A      4.6209 eV  268.31 nm  f=0.0018  <S**2>=0.000
   103 ->109        0.53468
   104 ->109        0.37734

Excited State  13:      Singlet-A      4.6648 eV  265.79 nm  f=0.0030  <S**2>=0.000
   103 ->109       -0.16028
   108 ->114        0.65100

Excited State  14:      Singlet-A      4.7937 eV  258.64 nm  f=0.0075  <S**2>=0.000
   103 ->109       -0.27279
   104 ->109        0.31824
   105 ->109        0.22594
   106 ->111        0.16089
   106 ->112       -0.27718
   108 ->113       -0.28768
   108 ->115        0.17105

Excited State  15:      Singlet-A      4.8110 eV  257.71 nm  f=0.0009  <S**2>=0.000
   106 ->111        0.47115
   107 ->114        0.35927
   108 ->113        0.31589

Excited State  16:      Singlet-A      4.8171 eV  257.38 nm  f=0.0016  <S**2>=0.000
```

```
    106 ->111       -0.38330
    107 ->114        0.54001

Excited State  17:      Singlet-A      4.8295 eV  256.72 nm  f=0.0023  <S**2>=0.000
    103 ->109       -0.11865
    104 ->109        0.13715
    106 ->111       -0.26033
    106 ->112       -0.15748
    108 ->113        0.53437
    108 ->114       -0.16062
    108 ->115        0.11626

Excited State  18:      Singlet-A      4.8881 eV  253.64 nm  f=0.0022  <S**2>=0.000
    102 ->110       -0.13781
    103 ->109       -0.11866
    104 ->109        0.13766
    104 ->110        0.18417
    105 ->109        0.16379
    105 ->110       -0.31947
    106 ->112        0.48423

Excited State  19:      Singlet-A      4.9129 eV  252.36 nm  f=0.0020  <S**2>=0.000
    102 ->110        0.18204
    104 ->109        0.13585
    104 ->110       -0.25868
    105 ->110        0.42906
    106 ->112        0.33190
    108 ->115        0.21941

Excited State  20:      Singlet-A      4.9614 eV  249.90 nm  f=0.0045  <S**2>=0.000
    103 ->109        0.10035
    104 ->109       -0.17235
    105 ->109       -0.12573
    105 ->110       -0.14568
    106 ->115        0.10135
    108 ->115        0.59798

Excited State  21:      Singlet-A      5.0582 eV  245.11 nm  f=0.0014  <S**2>=0.000
    103 ->110        0.40971
    104 ->110        0.37223
    105 ->110        0.23214
    107 ->115       -0.32671

Excited State  22:      Singlet-A      5.0736 eV  244.37 nm  f=0.0026  <S**2>=0.000
    103 ->110        0.25433
    104 ->110        0.21977
```

107 ->114 -0.14423
107 ->115 0.56034

Excited State 23: Singlet-A 5.1649 eV 240.05 nm f=0.0001 <S**2>=0.000
107 ->113 0.68643

Excited State 24: Singlet-A 5.1764 eV 239.52 nm f=0.0041 <S**2>=0.000
103 ->110 0.21197
104 ->110 -0.16147
106 ->114 0.56748
106 ->115 0.10326

Excited State 25: Singlet-A 5.2210 eV 237.47 nm f=0.0032 <S**2>=0.000
100 ->110 -0.10205
102 ->109 -0.19477
102 ->110 0.49233
103 ->110 -0.19244
104 ->110 0.30348
106 ->114 0.19541

Excited State 26: Singlet-A 5.2654 eV 235.47 nm f=0.0381 <S**2>=0.000
102 ->110 0.31138
103 ->110 0.39482
104 ->110 -0.21714
105 ->110 -0.30499
106 ->114 -0.17033
108 ->118 -0.10579

Excited State 27: Singlet-A 5.3508 eV 231.71 nm f=0.0005 <S**2>=0.000
104 ->111 -0.24353
105 ->109 -0.11246
105 ->111 0.62818

Excited State 28: Singlet-A 5.3770 eV 230.58 nm f=0.0047 <S**2>=0.000
104 ->111 -0.23112
105 ->112 0.22400
106 ->114 -0.19066
106 ->115 0.37895
108 ->118 0.38538

Excited State 29: Singlet-A 5.4155 eV 228.95 nm f=0.0010 <S**2>=0.000
103 ->111 0.36841
104 ->111 0.50908
105 ->111 0.17975
106 ->115 0.17259

Excited State 30:   Singlet-A   5.4212 eV 228.70 nm f=0.0032 <S**2>=0.000
104 ->112   -0.36107
105 ->112   0.51319
106 ->115   -0.23077

[Cu(I)LDHA]$^+$

Excitation energies and oscillator strengths:

Excited State   1:   Singlet-A   0.7816 eV 1586.26 nm f=0.0017 <S**2>=0.000
106 ->108   0.47647
107 ->108   0.51538
This state for optimization and/or second-order correction.
Total Energy, E(TD-HF/TD-DFT) = -2842.73322556
Copying the excited state density for this state as the 1-particle RhoCI density.

Excited State   2:   Singlet-A   1.4963 eV 828.59 nm f=0.0072 <S**2>=0.000
104 ->108   0.12193
105 ->108   0.66017
106 ->108   -0.13376
107 ->108   0.14141

Excited State   3:   Singlet-A   1.8059 eV 686.54 nm f=0.1494 <S**2>=0.000
101 ->108   -0.11422
102 ->108   0.14475
103 ->108   0.21182
105 ->108   0.18543
106 ->108   0.47488
107 ->108   -0.43450
106 <-108   -0.13374
107 <-108   0.13786

Excited State   4:   Singlet-A   2.1011 eV 590.09 nm f=0.0034 <S**2>=0.000
100 ->108   0.29916
101 ->108   0.37952
103 ->108   0.46886
104 ->108   -0.15865

Excited State   5:   Singlet-A   2.1876 eV 566.75 nm f=0.0617 <S**2>=0.000
100 ->108   0.29112
101 ->108   0.36776
102 ->108   -0.14983
103 ->108   -0.35448
104 ->108   0.22134
106 ->108   0.19261
107 ->108   -0.21018

```
Excited State   6:      Singlet-A      2.2147 eV  559.82 nm  f=0.0013  <S**2>=0.000
      99 ->108        -0.14126
     100 ->108         0.16563
     102 ->108         0.52354
     104 ->108         0.37492
     105 ->108        -0.13893

Excited State   7:      Singlet-A      2.8357 eV  437.22 nm  f=0.0003  <S**2>=0.000
     100 ->108        -0.12031
     102 ->108        -0.33911
     103 ->108         0.32284
     104 ->108         0.50933

Excited State   8:      Singlet-A      3.0803 eV  402.51 nm  f=0.0074  <S**2>=0.000
      98 ->108        -0.28772
      99 ->108         0.59264
     100 ->108        -0.13733
     102 ->108         0.17007

Excited State   9:      Singlet-A      3.1176 eV  397.69 nm  f=0.0021  <S**2>=0.000
      99 ->108         0.17296
     100 ->108         0.49842
     101 ->108        -0.43256
     102 ->108        -0.14558

Excited State  10:      Singlet-A      3.3760 eV  367.25 nm  f=0.0222  <S**2>=0.000
      98 ->108         0.63208
      99 ->108         0.28842

Excited State  11:      Singlet-A      3.6187 eV  342.62 nm  f=0.0010  <S**2>=0.000
      95 ->108         0.60633
      96 ->108        -0.29070
      97 ->108         0.13613

Excited State  12:      Singlet-A      3.8988 eV  318.00 nm  f=0.0054  <S**2>=0.000
      95 ->108         0.30997
      96 ->108         0.62378

Excited State  13:      Singlet-A      4.1363 eV  299.75 nm  f=0.0202  <S**2>=0.000
      96 ->108         0.12861
      97 ->108         0.66281

Excited State  14:      Singlet-A      4.4343 eV  279.60 nm  f=0.0087  <S**2>=0.000
     106 ->109         0.30160
     107 ->109         0.62688
```

Excited State 15: Singlet-A 4.5603 eV 271.87 nm f=0.0576 <S**2>=0.000
106 ->109 0.62288
107 ->109 -0.30226

Excited State 16: Singlet-A 4.7642 eV 260.24 nm f=0.0045 <S**2>=0.000
85 ->108 -0.23019
93 ->108 0.53265
94 ->108 0.36455

Excited State 17: Singlet-A 4.7810 eV 259.33 nm f=0.0002 <S**2>=0.000
93 ->108 -0.42377
94 ->108 0.55876

Excited State 18: Singlet-A 4.8164 eV 257.42 nm f=0.0040 <S**2>=0.000
92 ->108 0.69936

Excited State 19: Singlet-A 4.8584 eV 255.20 nm f=0.0026 <S**2>=0.000
85 ->108 0.51974
86 ->108 0.14524
89 ->108 -0.15673
91 ->108 0.12653
93 ->108 0.17540
94 ->108 0.21788
105 ->109 -0.19030

Excited State 20: Singlet-A 5.0881 eV 243.67 nm f=0.0011 <S**2>=0.000
90 ->108 -0.43466
91 ->108 0.19085
102 ->109 -0.11189
105 ->109 0.46805

Excited State 21: Singlet-A 5.1083 eV 242.71 nm f=0.0012 <S**2>=0.000
85 ->108 0.13834
90 ->108 0.54667
91 ->108 0.18041
105 ->109 0.34444

Excited State 22: Singlet-A 5.1588 eV 240.34 nm f=0.0010 <S**2>=0.000
85 ->108 -0.17587
91 ->108 0.63904
105 ->109 -0.19540

Excited State 23: Singlet-A 5.4023 eV 229.50 nm f=0.0019 <S**2>=0.000
86 ->108 0.17444
88 ->108 0.60669
89 ->108 0.29721

```
 Excited State  24:      Singlet-A      5.4127 eV  229.06 nm  f=0.0037  <S**2>=0.000
      89 ->108         0.11707
     106 ->110         0.15275
     107 ->110         0.66438

 Excited State  25:      Singlet-A      5.4331 eV  228.20 nm  f=0.0037  <S**2>=0.000
      85 ->108         0.18850
      88 ->108        -0.29638
      89 ->108         0.59348
     107 ->110        -0.10018

 Excited State  26:      Singlet-A      5.4511 eV  227.45 nm  f=0.0043  <S**2>=0.000
      86 ->108        -0.17721
      87 ->108         0.66732
      88 ->108         0.12062

 Excited State  27:      Singlet-A      5.5172 eV  224.72 nm  f=0.0011  <S**2>=0.000
     106 ->110         0.66845
     107 ->110        -0.16794

 Excited State  28:      Singlet-A      5.5375 eV  223.90 nm  f=0.0006  <S**2>=0.000
      85 ->108        -0.18011
      86 ->108         0.63090
      87 ->108         0.20784
      88 ->108        -0.15046

 Excited State  29:      Singlet-A      5.8102 eV  213.39 nm  f=0.0003  <S**2>=0.000
     100 ->109         0.14885
     102 ->109         0.30204
     103 ->109        -0.16060
     104 ->109         0.52665
     105 ->109         0.23452

 Excited State  30:      Singlet-A      5.9584 eV  208.08 nm  f=0.0027  <S**2>=0.000
     100 ->109        -0.10947
     102 ->109        -0.32993
     103 ->109         0.43766
     104 ->109         0.40855
```

M06 method:

```
 Excitation energies and oscillator strengths:

 Excited State   1:      Singlet-A      0.8774 eV 1413.15 nm  f=0.0018  <S**2>=0.000
      98 ->108         0.11947
     106 ->108         0.53180
```

```
    107 ->108         0.44791
This state for optimization and/or second-order correction.
Total Energy, E(TD-HF/TD-DFT) =  -2841.90336117
Copying the excited state density for this state as the 1-particle RhoCI density.

Excited State   2:      Singlet-A      1.5459 eV  802.04 nm  f=0.0114  <S**2>=0.000
    105 ->108         0.65800
    106 ->108        -0.12905
    107 ->108         0.18656

Excited State   3:      Singlet-A      1.8032 eV  687.59 nm  f=0.1491  <S**2>=0.000
    102 ->108        -0.15848
    103 ->108        -0.11922
    105 ->108        -0.21948
    106 ->108        -0.42925
    107 ->108         0.49647
    107 <-108        -0.13318

Excited State   4:      Singlet-A      2.1687 eV  571.70 nm  f=0.0010  <S**2>=0.000
    100 ->108        -0.17334
    101 ->108         0.49765
    102 ->108        -0.14056
    103 ->108         0.44026

Excited State   5:      Singlet-A      2.2483 eV  551.45 nm  f=0.0406  <S**2>=0.000
    101 ->108        -0.23296
    102 ->108         0.40445
    103 ->108         0.44038
    104 ->108        -0.17414
    106 ->108        -0.12617
    107 ->108         0.18006

Excited State   6:      Singlet-A      2.2921 eV  540.93 nm  f=0.0005  <S**2>=0.000
     99 ->108        -0.36818
    101 ->108         0.17743
    102 ->108         0.37444
    104 ->108         0.40256

Excited State   7:      Singlet-A      3.0918 eV  401.00 nm  f=0.0008  <S**2>=0.000
    101 ->108        -0.30684
    102 ->108        -0.25767
    103 ->108         0.26518
    104 ->108         0.50544

Excited State   8:      Singlet-A      3.1589 eV  392.49 nm  f=0.0063  <S**2>=0.000
     98 ->108         0.30239
```

```
     99 ->108         0.49016
    100 ->108        -0.21458
    102 ->108         0.24381
    103 ->108        -0.10325
    104 ->108         0.17348

 Excited State   9:      Singlet-A      3.3598 eV  369.02 nm  f=0.0059  <S**2>=0.000
     95 ->108        -0.10538
     98 ->108         0.24416
    100 ->108         0.60600
    101 ->108         0.21338

 Excited State  10:      Singlet-A      3.4159 eV  362.96 nm  f=0.0129  <S**2>=0.000
     98 ->108         0.56477
     99 ->108        -0.32708
    100 ->108        -0.16105
    102 ->108        -0.12879

 Excited State  11:      Singlet-A      3.7936 eV  326.82 nm  f=0.0009  <S**2>=0.000
     95 ->108         0.59227
     96 ->108        -0.24516
     97 ->108         0.14327
    100 ->108         0.11722

 Excited State  12:      Singlet-A      4.1061 eV  301.95 nm  f=0.0207  <S**2>=0.000
     95 ->108        -0.14656
     97 ->108         0.66815

 Excited State  13:      Singlet-A      4.1533 eV  298.52 nm  f=0.0068  <S**2>=0.000
     95 ->108         0.24793
     96 ->108         0.65140

 Excited State  14:      Singlet-A      4.6655 eV  265.75 nm  f=0.0299  <S**2>=0.000
     85 ->108         0.10228
    107 ->109         0.68018

 Excited State  15:      Singlet-A      4.7468 eV  261.19 nm  f=0.0286  <S**2>=0.000
     85 ->108         0.11844
    106 ->109         0.66199
    107 ->109        -0.12004

 Excited State  16:      Singlet-A      4.7557 eV  260.71 nm  f=0.0022  <S**2>=0.000
     93 ->108         0.68343

 Excited State  17:      Singlet-A      4.7725 eV  259.79 nm  f=0.0003  <S**2>=0.000
     94 ->108         0.69247
```

Excited State 18: Singlet-A 4.9484 eV 250.55 nm f=0.0024 <S**2>=0.000
82 ->108 0.15800
83 ->108 -0.12075
85 ->108 0.54939
92 ->108 -0.10988
105 ->109 -0.13857
106 ->109 -0.18311

Excited State 19: Singlet-A 5.0820 eV 243.97 nm f=0.0012 <S**2>=0.000
92 ->108 0.68721

Excited State 20: Singlet-A 5.1013 eV 243.04 nm f=0.0049 <S**2>=0.000
91 ->108 0.69462

Excited State 21: Singlet-A 5.2093 eV 238.00 nm f=0.0044 <S**2>=0.000
106 ->110 0.13148
107 ->110 0.67091

Excited State 22: Singlet-A 5.3006 eV 233.91 nm f=0.0006 <S**2>=0.000
85 ->108 0.11621
99 ->109 0.17276
104 ->109 -0.10176
105 ->109 0.44039
106 ->110 -0.43367

Excited State 23: Singlet-A 5.3140 eV 233.32 nm f=0.0009 <S**2>=0.000
85 ->108 0.11463
99 ->109 0.13739
105 ->109 0.35132
106 ->110 0.51409
107 ->110 -0.14325

Excited State 24: Singlet-A 5.3981 eV 229.68 nm f=0.0011 <S**2>=0.000
86 ->108 -0.32198
87 ->108 -0.10901
88 ->108 0.54717
89 ->108 0.22199
90 ->108 -0.14786

Excited State 25: Singlet-A 5.4311 eV 228.29 nm f=0.0029 <S**2>=0.000
86 ->108 0.10240
89 ->108 0.42222
90 ->108 0.54679

Excited State 26: Singlet-A 5.4484 eV 227.56 nm f=0.0041 <S**2>=0.000

```
     86 ->108         0.28285
     87 ->108         0.58543
     88 ->108         0.22520
     90 ->108        -0.13213

 Excited State  27:      Singlet-A      5.4564 eV  227.23 nm  f=0.0011  <S**2>=0.000
     85 ->108         0.13597
     88 ->108        -0.29275
     89 ->108         0.49153
     90 ->108        -0.36066

 Excited State  28:      Singlet-A      5.5232 eV  224.48 nm  f=0.0006  <S**2>=0.000
     86 ->108         0.53862
     87 ->108        -0.36768
     88 ->108         0.23714

 Excited State  29:      Singlet-A      6.0499 eV  204.94 nm  f=0.0003  <S**2>=0.000
     77 ->108         0.15929
     99 ->109        -0.24536
    101 ->109         0.23993
    102 ->109         0.17684
    104 ->109         0.43917
    105 ->109         0.31696

 Excited State  30:      Singlet-A      6.1211 eV  202.55 nm  f=0.0010  <S**2>=0.000
     83 ->108         0.23743
     84 ->108         0.65670
```

[Cu(II)L]$^{2+}$

Excitation energies and oscillator strengths:

```
 Excited State   1:  2.003-A      0.1803 eV 6874.70 nm  f=0.0005  <S**2>=0.753
    52B -> 62B       -0.20922
    56B -> 62B       -0.11044
    61B -> 62B        0.96758
    61B <- 62B       -0.15981
 This state for optimization and/or second-order correction.
 Total Energy, E(TD-HF/TD-DFT) =  -2159.20092213
 Copying the excited state density for this state as the 1-particle RhoCI density.

 Excited State   2:  2.005-A      1.9122 eV  648.38 nm  f=0.0011  <S**2>=0.755
    45B -> 62B       -0.10634
    51B -> 62B       -0.12445
    53B -> 62B        0.14232
    55B -> 62B        0.92407
    57B -> 62B        0.16497
```

```
      58B -> 62B         0.13027
      59B -> 62B        -0.10999
      60B -> 62B         0.11186

 Excited State   3:  2.004-A      1.9396 eV  639.23 nm  f=0.0011  <S**2>=0.754
      48B -> 62B        -0.15099
      49B -> 62B        -0.11071
      51B -> 62B         0.16563
      53B -> 62B        -0.11568
      54B -> 62B         0.91806
      56B -> 62B        -0.11247
      58B -> 62B         0.11491
      60B -> 62B         0.13200

 Excited State   4:  2.004-A      2.1276 eV  582.74 nm  f=0.0019  <S**2>=0.754
      48B -> 62B         0.91122
      52B -> 62B         0.22553
      54B -> 62B         0.17101
      56B -> 62B        -0.11801
      60B -> 62B        -0.12648

 Excited State   5:  2.007-A      2.3412 eV  529.57 nm  f=0.0263  <S**2>=0.757
      48B -> 62B         0.11244
      51B -> 62B         0.15704
      52B -> 62B         0.29275
      54B -> 62B        -0.13865
      55B -> 62B        -0.10463
      56B -> 62B         0.15656
      58B -> 62B         0.10630
      60B -> 62B         0.86429
      61B -> 62B         0.18532

 Excited State   6:  2.007-A      2.9141 eV  425.46 nm  f=0.0626  <S**2>=0.757
      48B -> 62B        -0.13377
      50B -> 62B         0.14694
      51B -> 62B         0.27085
      52B -> 62B         0.59007
      53B -> 62B         0.20396
      56B -> 62B         0.34871
      58B -> 62B         0.32324
      60B -> 62B        -0.43442
      61B -> 62B         0.22878

 Excited State   7:  3.450-A      3.2680 eV  379.39 nm  f=0.0004  <S**2>=2.725
      62A -> 63A        -0.62995
      61B -> 63B         0.75695
```

```
Excited State   8:  2.011-A      3.5718 eV  347.12 nm  f=0.0214  <S**2>=0.761
   60A -> 63A       -0.10368
   62A -> 63A       -0.25570
   57B -> 62B        0.15628
   59B -> 62B        0.90810
   61B -> 63B       -0.19032

Excited State   9:  2.008-A      3.6045 eV  343.97 nm  f=0.0030  <S**2>=0.758
   48B -> 62B        0.14168
   51B -> 62B       -0.11644
   52B -> 62B       -0.31520
   57B -> 62B       -0.26344
   58B -> 62B        0.87711

Excited State  10:  2.008-A      3.6222 eV  342.29 nm  f=0.0025  <S**2>=0.758
   62A -> 63A        0.10070
   53B -> 62B       -0.17415
   55B -> 62B       -0.16184
   56B -> 62B       -0.10181
   57B -> 62B        0.91372
   58B -> 62B        0.22609
   59B -> 62B       -0.11992

Excited State  11:  2.030-A      3.7829 eV  327.75 nm  f=0.0077  <S**2>=0.781
   62A -> 63A        0.70840
   59B -> 62B        0.31578
   61B -> 63B        0.59689

Excited State  12:  2.008-A      3.9531 eV  313.64 nm  f=0.0008  <S**2>=0.758
   52B -> 62B       -0.13834
   53B -> 62B        0.87597
   55B -> 62B       -0.14401
   56B -> 62B       -0.39957

Excited State  13:  2.008-A      4.0663 eV  304.90 nm  f=0.0108  <S**2>=0.758
   48B -> 62B        0.15251
   52B -> 62B       -0.43260
   53B -> 62B        0.26247
   54B -> 62B        0.21070
   55B -> 62B       -0.13485
   56B -> 62B        0.78771
   57B -> 62B        0.11064

Excited State  14:  2.009-A      4.2028 eV  295.01 nm  f=0.0069  <S**2>=0.760
   61A -> 63A       -0.14275
```

```
   48B -> 62B        0.10691
   51B -> 62B        0.88299
   52B -> 62B       -0.34418
   54B -> 62B       -0.13311
   55B -> 62B        0.14933

Excited State  15:  2.063-A      4.3102 eV  287.65 nm  f=0.0002  <S**2>=0.814
   61A -> 63A        0.91631
   50B -> 62B       -0.30336
   51B -> 62B        0.16402

Excited State  16:  2.013-A      4.3917 eV  282.31 nm  f=0.0147  <S**2>=0.763
   61A -> 63A        0.26886
   49B -> 62B       -0.40738
   50B -> 62B        0.83449
   52B -> 62B       -0.10737
   53B -> 62B       -0.12116

Excited State  17:  2.011-A      4.4241 eV  280.25 nm  f=0.0144  <S**2>=0.761
   61A -> 63A        0.18862
   49B -> 62B        0.88937
   50B -> 62B        0.37096

Excited State  18:  2.009-A      4.8896 eV  253.57 nm  f=0.0016  <S**2>=0.759
   45B -> 62B        0.11861
   46B -> 62B       -0.22275
   47B -> 62B        0.95065

Excited State  19:  2.009-A      5.0463 eV  245.69 nm  f=0.0039  <S**2>=0.759
   44B -> 62B       -0.20535
   46B -> 62B        0.93931
   47B -> 62B        0.21532

Excited State  20:  3.321-A      5.2638 eV  235.54 nm  f=0.0045  <S**2>=2.508
   60A -> 63A       -0.53217
   45B -> 62B        0.27257
   60B -> 63B        0.76509

Excited State  21:  2.138-A      5.2813 eV  234.76 nm  f=0.0023  <S**2>=0.893
   44B -> 62B       -0.25551
   45B -> 62B        0.89083
   47B -> 62B       -0.12632
   52B -> 62B       -0.10675
   60B -> 63B       -0.26332

Excited State  22:  2.012-A      5.3976 eV  229.70 nm  f=0.0123  <S**2>=0.762
```

```
      44B -> 62B         0.92639
      45B -> 62B         0.27375
      46B -> 62B         0.17882

 Excited State  23:  2.010-A      5.5612 eV  222.94 nm  f=0.0102  <S**2>=0.760
      43B -> 62B         0.98357

 Excited State  24:  3.132-A      5.7668 eV  214.99 nm  f=0.0024  <S**2>=2.203
      54A -> 63A        -0.27489
      55A -> 63A        -0.10126
      60A -> 63A        -0.13132
      54B -> 63B         0.17003
      55B -> 63B         0.62278
      58B -> 63B         0.17276
      59B -> 63B        -0.60900
      60B -> 63B        -0.15650

 Excited State  25:  2.252-A      5.8006 eV  213.74 nm  f=0.0364  <S**2>=1.018
      52A -> 63A        -0.11485
      60A -> 63A         0.68817
      42B -> 62B        -0.11245
      54B -> 63B        -0.26088
      59B -> 62B         0.14305
      59B -> 63B        -0.29213
      60B -> 63B         0.49065

 Excited State  26:  3.141-A      5.8352 eV  212.48 nm  f=0.0089  <S**2>=2.216
      51A -> 63A         0.10575
      53A -> 63A         0.27081
      54A -> 63A        -0.26437
      60A -> 63A         0.27931
      54B -> 63B         0.51296
      55B -> 63B         0.31848
      59B -> 63B         0.57578
      60B -> 63B         0.11686

 Excited State  27:  3.295-A      5.8714 eV  211.17 nm  f=0.0037  <S**2>=2.464
      52A -> 63A         0.20930
      53A -> 63A         0.32081
      54A -> 63A         0.11760
      55A -> 63A         0.15672
      51B -> 63B         0.16046
      53B -> 63B        -0.14992
      54B -> 63B         0.54142
      55B -> 63B        -0.42188
      56B -> 63B        -0.18049
```

```
    57B -> 63B       -0.18706
    59B -> 63B       -0.38848

 Excited State  28:  2.200-A      6.1372 eV  202.02 nm  f=0.0050  <S**2>=0.960
    52A -> 63A        0.29730
    54A -> 63A        0.67015
    55A -> 63A        0.36727
    56A -> 63A       -0.10239
    58A -> 63A        0.21355
    59A -> 63A        0.11011
    55B -> 63B        0.43138
    57B -> 63B        0.11419

 Excited State  29:  2.592-A      6.2033 eV  199.87 nm  f=0.0047  <S**2>=1.429
    50A -> 63A       -0.18627
    51A -> 63A        0.17408
    52A -> 63A        0.32907
    53A -> 63A        0.46390
    54A -> 63A       -0.20569
    55A -> 63A        0.11897
    59A -> 63A       -0.33927
    52B -> 63B        0.25643
    53B -> 63B        0.13497
    54B -> 63B       -0.42990
    56B -> 63B        0.24110
    58B -> 63B        0.15928

 Excited State  30:  2.994-A      6.2227 eV  199.25 nm  f=0.0033  <S**2>=1.992
    50A -> 63A        0.11245
    51A -> 63A        0.14775
    53A -> 63A        0.49972
    54A -> 63A       -0.10431
    57A -> 63A        0.12692
    59A -> 63A        0.11894
    60A -> 63A       -0.10374
    42B -> 62B        0.23906
    50B -> 63B       -0.13054
    51B -> 63B       -0.21915
    52B -> 63B       -0.43311
    53B -> 63B       -0.12080
    54B -> 63B       -0.18475
    56B -> 63B       -0.24455
    58B -> 63B       -0.42864
```

[Cu(II)L(H_2O)]$^{2+}$

```
 Excitation energies and oscillator strengths:
```

```
Excited State   1:  2.008-A      0.5645 eV 2196.26 nm  f=0.0042  <S**2>=0.758
    55B -> 67B        0.10170
    58B -> 67B        0.21313
    59B -> 67B        0.19350
    66B -> 67B        0.94580
    66B <- 67B       -0.10174
This state for optimization and/or second-order correction.
Total Energy, E(TD-HF/TD-DFT) =  -2235.61503936
Copying the excited state density for this state as the 1-particle RhoCI density.

Excited State   2:  2.003-A      2.0013 eV  619.53 nm  f=0.0001  <S**2>=0.753
    48B -> 67B       -0.20780
    50B -> 67B       -0.12082
    54B -> 67B       -0.14627
    58B -> 67B       -0.10474
    60B -> 67B        0.60730
    61B -> 67B       -0.60079
    62B -> 67B       -0.19084
    63B -> 67B        0.19827
    64B -> 67B        0.25189

Excited State   3:  2.003-A      2.0752 eV  597.46 nm  f=0.0013  <S**2>=0.753
    50B -> 67B       -0.11633
    58B -> 67B       -0.10783
    59B -> 67B       -0.10952
    60B -> 67B        0.63050
    61B -> 67B        0.53468
    62B -> 67B        0.48306
    63B -> 67B       -0.12004

Excited State   4:  2.003-A      2.1909 eV  565.91 nm  f=0.0001  <S**2>=0.753
    49B -> 67B       -0.11507
    54B -> 67B        0.37595
    55B -> 67B       -0.35971
    56B -> 67B        0.60556
    57B -> 67B        0.51271
    58B -> 67B        0.20745
    63B -> 67B        0.13809

Excited State   5:  2.011-A      2.8197 eV  439.70 nm  f=0.0618  <S**2>=0.761
    61B -> 67B       -0.10920
    64B -> 67B       -0.14948
    65B -> 67B        0.97175

Excited State   6:  2.027-A      3.0454 eV  407.11 nm  f=0.0278  <S**2>=0.777
```

```
    54B -> 67B       -0.17825
    55B -> 67B        0.23227
    57B -> 67B        0.10795
    58B -> 67B        0.59670
    59B -> 67B        0.55351
    60B -> 67B        0.15029
    63B -> 67B       -0.25671
    66B -> 67B       -0.33278

 Excited State   7:  2.006-A      3.6960 eV  335.46 nm  f=0.0373  <S**2>=0.756
    60B -> 67B       -0.18603
    61B -> 67B        0.13434
    64B -> 67B        0.94142
    65B -> 67B        0.16058

 Excited State   8:  2.014-A      4.0731 eV  304.40 nm  f=0.0022  <S**2>=0.765
    55B -> 67B        0.10540
    56B -> 67B       -0.13414
    58B -> 67B        0.16922
    59B -> 67B        0.18194
    61B -> 67B        0.12151
    62B -> 67B        0.24734
    63B -> 67B        0.90583

 Excited State   9:  2.013-A      4.1066 eV  301.92 nm  f=0.0031  <S**2>=0.763
    60B -> 67B       -0.20788
    61B -> 67B       -0.51579
    62B -> 67B        0.81242
    63B -> 67B       -0.14520

 Excited State  10:  2.013-A      4.4104 eV  281.12 nm  f=0.0002  <S**2>=0.763
    54B -> 67B        0.13627
    55B -> 67B       -0.10240
    58B -> 67B       -0.62766
    59B -> 67B        0.74801

 Excited State  11:  2.026-A      4.6932 eV  264.18 nm  f=0.0007  <S**2>=0.776
    54B -> 67B       -0.13960
    55B -> 67B        0.42351
    56B -> 67B       -0.26955
    57B -> 67B        0.80045
    58B -> 67B       -0.20283
    59B -> 67B       -0.16807

 Excited State  12:  2.080-A      4.7584 eV  260.56 nm  f=0.0106  <S**2>=0.831
    54B -> 67B       -0.57644
```

55B -> 67B 0.26879
56B -> 67B 0.66542
57B -> 67B -0.11248
58B -> 67B -0.21306
60B -> 67B -0.20366
66B -> 68B 0.17670

Excited State 13: 2.394-A 4.8013 eV 258.23 nm f=0.0128 <S**2>=1.183
67A -> 68A 0.16797
53B -> 67B -0.12208
54B -> 67B 0.59178
55B -> 67B 0.53529
56B -> 67B 0.12628
57B -> 67B -0.20253
66B -> 68B 0.47076

Excited State 14: 2.895-A 4.8188 eV 257.29 nm f=0.0066 <S**2>=1.846
67A -> 68A 0.25767
54B -> 67B -0.19985
55B -> 67B -0.47374
56B -> 67B -0.21884
66B -> 68B 0.76722

Excited State 15: 2.025-A 4.9364 eV 251.17 nm f=0.0014 <S**2>=0.775
53B -> 67B 0.97947

Excited State 16: 2.307-A 5.1466 eV 240.90 nm f=0.0002 <S**2>=1.081
67A -> 68A 0.93097
66B -> 68B -0.33155

Excited State 17: 2.156-A 5.2481 eV 236.25 nm f=0.0016 <S**2>=0.912
66A -> 68A 0.97808

Excited State 18: 2.015-A 5.3772 eV 230.58 nm f=0.0024 <S**2>=0.765
48B -> 67B 0.21475
50B -> 67B 0.27557
52B -> 67B 0.91541

Excited State 19: 2.015-A 5.4748 eV 226.46 nm f=0.0031 <S**2>=0.765
47B -> 67B 0.11605
48B -> 67B -0.50059
50B -> 67B -0.26741
51B -> 67B 0.72379
52B -> 67B 0.27892
60B -> 67B -0.17811

Excited State 20: 2.015-A   5.5508 eV 223.36 nm f=0.0020 <S**2>=0.765
47B -> 67B   -0.13334
48B -> 67B   0.43683
49B -> 67B   0.27701
50B -> 67B   0.45710
51B -> 67B   0.64555
52B -> 67B   -0.22886
60B -> 67B   0.12323

Excited State 21: 2.015-A   5.8505 eV 211.92 nm f=0.0062 <S**2>=0.765
47B -> 67B   0.10769
48B -> 67B   -0.34490
49B -> 67B   -0.60412
50B -> 67B   0.68488
52B -> 67B   -0.12294

Excited State 22: 2.016-A   5.8765 eV 210.98 nm f=0.0038 <S**2>=0.766
48B -> 67B   -0.54571
49B -> 67B   0.71159
50B -> 67B   0.36497
51B -> 67B   -0.19859

Excited State 23: 2.015-A   6.0651 eV 204.42 nm f=0.0105 <S**2>=0.765
47B -> 67B   0.96466
48B -> 67B   0.17354
49B -> 67B   0.15183

Excited State 24: 3.221-A   6.5163 eV 190.27 nm f=0.0034 <S**2>=2.344
65A -> 68A   -0.31791
64B -> 68B   0.10046
65B -> 68B   0.90628
66B -> 70B   0.14669

Excited State 25: 2.056-A   6.7475 eV 183.75 nm f=0.0008 <S**2>=0.806
65A -> 68A   -0.18844
46B -> 67B   0.95354

Excited State 26: 2.230-A   6.7721 eV 183.08 nm f=0.0018 <S**2>=0.994
65A -> 68A   -0.46029
45B -> 67B   0.80147
46B -> 67B   -0.18286
64B -> 68B   -0.19781
65B -> 68B   -0.18792

Excited State 27: 2.496-A   6.8809 eV 180.19 nm f=0.0047 <S**2>=1.308
65A -> 68A   0.50994

```
   44B -> 67B        0.48452
   45B -> 67B        0.49237
   64B -> 68B        0.42698
   65B -> 68B        0.13564

 Excited State  28:  2.262-A      6.9135 eV  179.34 nm  f=0.0029  <S**2>=1.029
   65A -> 68A       -0.28059
   44B -> 67B        0.82762
   45B -> 67B       -0.24834
   46B -> 67B       -0.13816
   61B -> 68B        0.12461
   64B -> 68B       -0.22145
   66B -> 69B        0.19788

 Excited State  29:  3.015-A      6.9545 eV  178.28 nm  f=0.0009  <S**2>=2.022
   67A -> 69A        0.12034
   44B -> 67B       -0.16165
   58B -> 68B       -0.11870
   66B -> 69B        0.91704

 Excited State  30:  2.826-A      7.0232 eV  176.54 nm  f=0.0193  <S**2>=1.747
   61A -> 68A        0.18243
   62A -> 68A        0.21487
   65A -> 68A       -0.43575
   45B -> 67B       -0.15346
   61B -> 68B       -0.36045
   62B -> 68B       -0.20112
   63B -> 68B        0.13141
   64B -> 68B        0.57350
   65B -> 68B       -0.25296
   66B -> 70B        0.19099
```

$[Cu(II)L(H_2O)_2]^{2+}$

```
 Excitation energies and oscillator strengths:

 Excited State   1:  2.003-A      0.6904 eV 1795.93 nm  f=0.0012  <S**2>=0.753
   61B -> 72B       -0.12038
   64B -> 72B       -0.23894
   66B -> 72B       -0.22811
   71B -> 72B        0.91872
 This state for optimization and/or second-order correction.
 Total Energy, E(TD-HF/TD-DFT) =  -2312.01230810
 Copying the excited state density for this state as the 1-particle RhoCI density.

 Excited State   2:  2.002-A      2.1200 eV  584.84 nm  f=0.0006  <S**2>=0.752
   52B -> 72B       -0.14601
```

54B -> 72B -0.13936
57B -> 72B 0.14078
59B -> 72B 0.12191
60B -> 72B -0.15642
64B -> 72B 0.24184
65B -> 72B 0.70117
66B -> 72B -0.43080
67B -> 72B -0.22575
68B -> 72B -0.19197
69B -> 72B 0.15596

Excited State 3: 2.002-A 2.1600 eV 574.00 nm f=0.0006 <S**2>=0.752
62B -> 72B -0.13769
63B -> 72B -0.10743
64B -> 72B -0.48940
65B -> 72B 0.52056
66B -> 72B 0.62764
67B -> 72B -0.11289
69B -> 72B 0.10434

Excited State 4: 2.002-A 2.2583 eV 549.01 nm f=0.0006 <S**2>=0.752
53B -> 72B -0.11504
54B -> 72B 0.22226
57B -> 72B 0.31058
58B -> 72B 0.52322
60B -> 72B 0.33957
61B -> 72B 0.49639
62B -> 72B -0.34173
63B -> 72B -0.13392
66B -> 72B -0.12724
68B -> 72B -0.12499

Excited State 5: 2.005-A 2.8681 eV 432.28 nm f=0.0096 <S**2>=0.755
61B -> 72B 0.15585
62B -> 72B 0.12889
64B -> 72B 0.42208
65B -> 72B 0.12923
66B -> 72B 0.35436
68B -> 72B -0.17675
70B -> 72B 0.67798
71B -> 72B 0.33914

Excited State 6: 2.006-A 3.3611 eV 368.88 nm f=0.0679 <S**2>=0.756
61B -> 72B -0.22244
62B -> 72B -0.11867
64B -> 72B -0.45401

66B -> 72B -0.35805
68B -> 72B 0.17357
69B -> 72B 0.10532
70B -> 72B 0.70065
71B -> 72B -0.20039

Excited State 7: 2.006-A 3.8849 eV 319.14 nm f=0.0653 <S**2>=0.756
65B -> 72B -0.17541
69B -> 72B 0.95923
70B -> 72B -0.10814

Excited State 8: 2.006-A 4.4589 eV 278.06 nm f=0.0014 <S**2>=0.756
61B -> 72B 0.10027
64B -> 72B 0.27023
65B -> 72B 0.19426
67B -> 72B 0.19880
68B -> 72B 0.90690

Excited State 9: 2.006-A 4.5106 eV 274.88 nm f=0.0015 <S**2>=0.756
63B -> 72B -0.13182
64B -> 72B -0.12751
65B -> 72B 0.18621
67B -> 72B 0.93155
68B -> 72B -0.19691

Excited State 10: 2.007-A 4.8493 eV 255.67 nm f=0.0007 <S**2>=0.757
62B -> 72B -0.25121
63B -> 72B 0.94220
65B -> 72B 0.14219

Excited State 11: 3.275-A 4.9732 eV 249.30 nm f=0.0002 <S**2>=2.431
72A -> 73A -0.36873
71B -> 73B 0.91421

Excited State 12: 2.010-A 5.0632 eV 244.88 nm f=0.0062 <S**2>=0.760
57B -> 72B -0.16935
59B -> 72B -0.20249
60B -> 72B 0.21215
61B -> 72B 0.45841
62B -> 72B 0.70102
64B -> 72B -0.25283
65B -> 72B 0.19856
66B -> 72B -0.22745

Excited State 13: 2.011-A 5.1466 eV 240.91 nm f=0.0060 <S**2>=0.761
57B -> 72B -0.26452

```
     58B -> 72B       -0.40198
     59B -> 72B       -0.16904
     60B -> 72B       -0.39272
     61B -> 72B        0.59463
     62B -> 72B       -0.41743
     63B -> 72B       -0.14406

 Excited State  14:  2.278-A      5.1506 eV  240.72 nm  f=0.0004  <S**2>=1.047
     72A -> 73A        0.90054
     60B -> 72B       -0.14320
     61B -> 72B        0.11593
     71B -> 73B        0.35871

 Excited State  15:  2.013-A      5.1925 eV  238.77 nm  f=0.0043  <S**2>=0.763
     72A -> 73A        0.16739
     57B -> 72B       -0.34378
     58B -> 72B       -0.23979
     59B -> 72B       -0.38602
     60B -> 72B        0.67767
     61B -> 72B       -0.15547
     62B -> 72B       -0.27844
     63B -> 72B       -0.10403
     64B -> 72B        0.20743

 Excited State  16:  2.007-A      5.3098 eV  233.50 nm  f=0.0100  <S**2>=0.757
     54B -> 72B       -0.11084
     57B -> 72B        0.16362
     58B -> 72B       -0.50308
     59B -> 72B        0.69983
     60B -> 72B        0.38718
     61B -> 72B        0.11488
     64B -> 72B       -0.17337

 Excited State  17:  2.007-A      5.4966 eV  225.56 nm  f=0.0003  <S**2>=0.757
     56B -> 72B        0.11091
     57B -> 72B        0.76089
     58B -> 72B       -0.37212
     59B -> 72B       -0.48879

 Excited State  18:  2.218-A      5.7127 eV  217.03 nm  f=0.0004  <S**2>=0.980
     71A -> 73A        0.98002

 Excited State  19:  2.009-A      5.8146 eV  213.23 nm  f=0.0009  <S**2>=0.759
     52B -> 72B       -0.15977
     54B -> 72B       -0.32326
     56B -> 72B        0.89023
```

```
      57B -> 72B       -0.11211
      58B -> 72B        0.13609

 Excited State  20:  2.010-A      5.8573 eV  211.67 nm  f=0.0026  <S**2>=0.760
      52B -> 72B        0.26823
      53B -> 72B       -0.16205
      54B -> 72B        0.54977
      55B -> 72B        0.61178
      56B -> 72B        0.34743
      57B -> 72B       -0.10473
      58B -> 72B       -0.18622
      59B -> 72B        0.10531

 Excited State  21:  2.008-A      5.9539 eV  208.24 nm  f=0.0011  <S**2>=0.758
      53B -> 72B        0.19725
      54B -> 72B       -0.57338
      55B -> 72B        0.74916
      56B -> 72B       -0.16612
      58B -> 72B        0.14073

 Excited State  22:  2.007-A      6.2903 eV  197.10 nm  f=0.0014  <S**2>=0.757
      53B -> 72B        0.93971
      54B -> 72B        0.24183
      56B -> 72B        0.16458

 Excited State  23:  2.009-A      6.4029 eV  193.64 nm  f=0.0073  <S**2>=0.759
      51B -> 72B       -0.21389
      52B -> 72B        0.89693
      54B -> 72B       -0.31309
      55B -> 72B       -0.16374

 Excited State  24:  3.221-A      6.4815 eV  191.29 nm  f=0.0014  <S**2>=2.343
      70A -> 73A       -0.10930
      72A -> 74A       -0.25787
      72A -> 75A       -0.13766
      70B -> 73B        0.24455
      71B -> 74B        0.86602
      71B -> 75B        0.23211

 Excited State  25:  2.008-A      6.5305 eV  189.86 nm  f=0.0061  <S**2>=0.758
      51B -> 72B        0.96604
      52B -> 72B        0.22759

 Excited State  26:  2.366-A      6.6524 eV  186.37 nm  f=0.0010  <S**2>=1.150
      70A -> 73A        0.10594
      72A -> 74A        0.85911
```

```
    72A -> 75A        0.15693
    70B -> 73B       -0.14445
    71B -> 74B        0.39827
    71B -> 75B       -0.16446

 Excited State  27:  3.267-A      6.7592 eV  183.43 nm  f=0.0022  <S**2>=2.419
    70A -> 73A       -0.18173
    72A -> 74A        0.40658
    72A -> 75A       -0.20314
    70B -> 73B        0.64153
    71B -> 74B       -0.27567
    71B -> 75B        0.48413

 Excited State  28:  3.035-A      6.8952 eV  179.81 nm  f=0.0031  <S**2>=2.053
    72A -> 75A       -0.21405
    69B -> 73B        0.11114
    70B -> 73B       -0.66606
    71B -> 75B        0.68055

 Excited State  29:  2.337-A      7.0160 eV  176.72 nm  f=0.0033  <S**2>=1.115
    70A -> 73A        0.31515
    72A -> 75A        0.73404
    69B -> 73B       -0.37086
    70B -> 73B        0.10071
    71B -> 75B        0.41548

 Excited State  30:  2.818-A      7.0330 eV  176.29 nm  f=0.0054  <S**2>=1.736
    72A -> 75A        0.37751
    69B -> 73B        0.89469
```

$[Cu(II)L(H_2O)_3]^{2+}$

```
Excitation energies and oscillator strengths:

 Excited State   1:  2.002-A      0.4848 eV 2557.55 nm  f=0.0005  <S**2>=0.752
    71B -> 77B        0.27131
    73B -> 77B       -0.27159
    75B -> 77B       -0.18163
    76B -> 77B        0.88887
 This state for optimization and/or second-order correction.
 Total Energy, E(TD-HF/TD-DFT) =  -2388.65843567
 Copying the excited state density for this state as the 1-particle RhoCI density.

 Excited State   2:  2.001-A      1.8872 eV  656.97 nm  f=0.0002  <S**2>=0.751
    60B -> 77B        0.16389
    64B -> 77B        0.10618
    65B -> 77B       -0.21165
```

```
    68B -> 77B        0.26414
    70B -> 77B        0.37971
    71B -> 77B       -0.29477
    72B -> 77B        0.75256
    75B -> 77B       -0.12869

 Excited State   3:  2.001-A      1.9231 eV  644.72 nm  f=0.0001  <S**2>=0.751
    67B -> 77B       -0.14107
    69B -> 77B        0.58953
    70B -> 77B        0.30308
    71B -> 77B        0.55440
    72B -> 77B        0.16433
    73B -> 77B        0.41616

 Excited State   4:  2.002-A      2.1001 eV  590.38 nm  f=0.0004  <S**2>=0.752
    60B -> 77B        0.39961
    61B -> 77B        0.11304
    62B -> 77B        0.38362
    63B -> 77B       -0.18840
    64B -> 77B       -0.24455
    65B -> 77B        0.19459
    66B -> 77B       -0.25487
    67B -> 77B        0.57731
    68B -> 77B       -0.14046
    70B -> 77B        0.28916
    72B -> 77B       -0.11905

 Excited State   5:  2.004-A      2.9002 eV  427.50 nm  f=0.0013  <S**2>=0.754
    71B -> 77B       -0.43893
    73B -> 77B        0.39468
    75B -> 77B        0.65236
    76B -> 77B        0.42667

 Excited State   6:  2.005-A      3.5546 eV  348.80 nm  f=0.0860  <S**2>=0.755
    67B -> 77B        0.15784
    71B -> 77B        0.34288
    72B -> 77B        0.16344
    73B -> 77B       -0.52191
    75B -> 77B        0.70859
    76B -> 77B       -0.16633

 Excited State   7:  2.004-A      3.9878 eV  310.91 nm  f=0.0818  <S**2>=0.754
    74B -> 77B        0.98187

 Excited State   8:  2.005-A      4.5862 eV  270.34 nm  f=0.0007  <S**2>=0.755
    67B -> 77B        0.16817
```

69B -> 77B -0.47639
70B -> 77B -0.47593
71B -> 77B 0.31375
72B -> 77B 0.40842
73B -> 77B 0.49608

Excited State 9: 2.006-A 4.7669 eV 260.09 nm f=0.0006 <S**2>=0.756
62B -> 77B -0.10202
63B -> 77B 0.12384
64B -> 77B 0.19463
65B -> 77B -0.30229
66B -> 77B 0.16751
68B -> 77B 0.31025
69B -> 77B -0.44793
70B -> 77B 0.51193
71B -> 77B 0.27066
72B -> 77B -0.37140
73B -> 77B 0.17618

Excited State 10: 2.006-A 4.9417 eV 250.89 nm f=0.0019 <S**2>=0.756
60B -> 77B 0.12345
62B -> 77B 0.10086
66B -> 77B -0.12436
67B -> 77B 0.13335
68B -> 77B 0.85564
69B -> 77B 0.28008
70B -> 77B -0.31207
72B -> 77B -0.14530

Excited State 11: 2.007-A 5.0592 eV 245.07 nm f=0.0020 <S**2>=0.757
64B -> 77B 0.21479
65B -> 77B -0.54324
66B -> 77B 0.25778
67B -> 77B 0.55946
68B -> 77B -0.20880
69B -> 77B 0.35290
70B -> 77B -0.19726
71B -> 77B -0.15548
73B -> 77B 0.14716

Excited State 12: 2.006-A 5.2659 eV 235.45 nm f=0.0010 <S**2>=0.756
60B -> 77B 0.14515
62B -> 77B 0.17929
63B -> 77B -0.11240
64B -> 77B -0.14080
65B -> 77B 0.30894

```
      66B -> 77B        0.89603

 Excited State  13:  2.008-A      5.3865 eV  230.18 nm  f=0.0035  <S**2>=0.758
      60B -> 77B        0.49673
      61B -> 77B        0.12145
      62B -> 77B        0.44078
      65B -> 77B       -0.48233
      67B -> 77B       -0.47903
      68B -> 77B       -0.12336
      70B -> 77B       -0.17047
      72B -> 77B       -0.12210

 Excited State  14:  3.036-A      5.5774 eV  222.30 nm  f=0.0002  <S**2>=2.054
      77A -> 78A       -0.16046
      76B -> 78B        0.97132

 Excited State  15:  2.009-A      5.6007 eV  221.37 nm  f=0.0118  <S**2>=0.759
      62B -> 77B        0.10012
      63B -> 77B       -0.64331
      64B -> 77B        0.72332
      65B -> 77B        0.17341

 Excited State  16:  2.018-A      5.6093 eV  221.03 nm  f=0.0030  <S**2>=0.768
      60B -> 77B        0.19649
      62B -> 77B        0.21730
      63B -> 77B        0.70094
      64B -> 77B        0.51687
      65B -> 77B        0.35817
      76B -> 78B       -0.10563

 Excited State  17:  2.594-A      5.7707 eV  214.85 nm  f=0.0006  <S**2>=1.432
      77A -> 78A        0.97939
      76B -> 78B        0.16516

 Excited State  18:  2.007-A      5.7859 eV  214.29 nm  f=0.0069  <S**2>=0.757
      60B -> 77B       -0.63973
      61B -> 77B       -0.14615
      62B -> 77B        0.72898

 Excited State  19:  2.006-A      5.8382 eV  212.37 nm  f=0.0014  <S**2>=0.756
      60B -> 77B       -0.21887
      61B -> 77B        0.96232

 Excited State  20:  2.006-A      6.2919 eV  197.05 nm  f=0.0003  <S**2>=0.756
      57B -> 77B       -0.12762
      58B -> 77B        0.24664
```

59B -> 77B 0.95016

Excited State 21: 2.008-A 6.3402 eV 195.55 nm f=0.0005 <S**2>=0.758
56B -> 77B 0.14402
58B -> 77B 0.94726
59B -> 77B -0.23397

Excited State 22: 2.254-A 6.4115 eV 193.38 nm f=0.0004 <S**2>=1.021
76A -> 78A 0.98878

Excited State 23: 3.023-A 6.6628 eV 186.08 nm f=0.0009 <S**2>=2.034
77A -> 79A -0.13835
76B -> 79B 0.97517

Excited State 24: 2.009-A 6.7197 eV 184.51 nm f=0.0044 <S**2>=0.759
54B -> 77B 0.13353
55B -> 77B 0.11239
57B -> 77B 0.96313
59B -> 77B 0.14364

Excited State 25: 2.016-A 6.7882 eV 182.65 nm f=0.0046 <S**2>=0.766
55B -> 77B 0.12214
56B -> 77B 0.96818
58B -> 77B -0.12217

Excited State 26: 2.928-A 6.8276 eV 181.59 nm f=0.0010 <S**2>=1.894
77A -> 79A 0.33717
76B -> 80B 0.90943
76B -> 81B -0.11152

Excited State 27: 2.718-A 6.8557 eV 180.85 nm f=0.0011 <S**2>=1.596
77A -> 79A 0.91094
77A -> 80A 0.14873
76B -> 79B 0.14664
76B -> 80B -0.32464

Excited State 28: 2.007-A 6.9105 eV 179.41 nm f=0.0048 <S**2>=0.757
54B -> 77B 0.18147
55B -> 77B 0.96082
56B -> 77B -0.10302
57B -> 77B -0.13895

Excited State 29: 2.641-A 7.0182 eV 176.66 nm f=0.0006 <S**2>=1.494
77A -> 80A 0.93645
77A -> 81A -0.15596
76B -> 80B 0.16645

76B -> 81B 0.18310

Excited State 30: 3.013-A 7.0706 eV 175.35 nm f=0.0046 <S**2>=2.020
77A -> 80A -0.21947
77A -> 81A -0.11735
76B -> 81B 0.95036
76B -> 82B -0.10832

[Cu(II)LAsc], conformer 1

TD-B3LYP method:
Excitation energies and oscillator strengths:

Excited State 1: 2.015-A 1.5764 eV 786.50 nm f=0.0002 <S**2>=0.765
102B ->108B 0.11665
105B ->108B 0.42276
106B ->108B 0.87275
107B ->108B 0.15093
This state for optimization and/or second-order correction.
Total Energy, E(TD-HF/TD-DFT) = -2842.84036663
Copying the excited state density for this state as the 1-particle RhoCI density.

Excited State 2: 2.012-A 1.6634 eV 745.35 nm f=0.0123 <S**2>=0.762
105B ->108B 0.70490
106B ->108B -0.22858
107B ->108B -0.66320

Excited State 3: 2.013-A 1.8898 eV 656.06 nm f=0.0484 <S**2>=0.763
105B ->108B 0.54676
106B ->108B -0.40440
107B ->108B 0.71793

Excited State 4: 2.030-A 2.3111 eV 536.46 nm f=0.0011 <S**2>=0.780
102B ->108B -0.60064
103B ->108B -0.29000
104B ->108B 0.70325
105B ->108B 0.11393
106B ->108B 0.10188

Excited State 5: 2.013-A 2.4365 eV 508.86 nm f=0.0009 <S**2>=0.763
102B ->108B 0.72936
103B ->108B -0.53616
104B ->108B 0.41667

Excited State 6: 2.013-A 2.4638 eV 503.22 nm f=0.0044 <S**2>=0.763
102B ->108B 0.26879

```
     103B ->108B        0.78726
     104B ->108B        0.54755

 Excited State   7:  2.146-A      3.3016 eV  375.52 nm  f=0.0002  <S**2>=0.901
      99B ->108B        0.14548
     100B ->108B        0.53178
     101B ->108B        0.76646
     102B ->112B       -0.12491
     104B ->112B        0.11625

 Excited State   8:  2.082-A      3.6428 eV  340.35 nm  f=0.0128  <S**2>=0.834
     106A ->110A       -0.10589
     108A ->109A       -0.39508
     108A ->110A        0.59243
      93B ->108B        0.10102
      94B ->108B        0.10057
      99B ->108B       -0.39166
     100B ->108B        0.42926
     101B ->108B       -0.23960
     107B ->112B        0.12035

 Excited State   9:  2.075-A      3.9063 eV  317.40 nm  f=0.0269  <S**2>=0.826
      99A ->110A       -0.10306
     108A ->109A        0.52808
     108A ->110A       -0.33368
      93B ->108B       -0.13404
      94B ->108B       -0.10890
      99B ->108B       -0.37560
     100B ->108B        0.52677
     101B ->108B       -0.30147

 Excited State  10:  2.376-A      3.9471 eV  314.12 nm  f=0.0029  <S**2>=1.162
     106A ->109A        0.13786
     108A ->109A        0.61067
     108A ->110A        0.59772
     108A ->111A        0.24411
     108A ->115A        0.15093
      99B ->108B        0.11645
     100B ->108B       -0.16244
     107B ->109B        0.19325

 Excited State  11:  2.092-A      4.0991 eV  302.46 nm  f=0.0007  <S**2>=0.844
      93B ->108B       -0.20716
      94B ->108B        0.41363
      95B ->108B       -0.41621
      96B ->108B        0.13818
```

```
     98B ->108B       -0.22054
     99B ->108B        0.52268
    100B ->108B        0.24419
    101B ->108B       -0.38212

 Excited State  12:  2.847-A      4.1543 eV  298.44 nm  f=0.0008  <S**2>=1.777
    108A ->109A       -0.19179
    108A ->110A       -0.17458
     99B ->108B       -0.16014
    107B ->109B        0.83693
    107B ->110B        0.29128
    107B ->115B        0.18112

 Excited State  13:  2.096-A      4.1906 eV  295.86 nm  f=0.0008  <S**2>=0.849
     93B ->108B        0.27039
     94B ->108B       -0.36689
     95B ->108B        0.43396
     96B ->108B       -0.12239
     98B ->108B        0.20358
     99B ->108B        0.55774
    100B ->108B        0.32136
    101B ->108B       -0.24556
    107B ->109B        0.15361

 Excited State  14:  3.426-A      4.2845 eV  289.38 nm  f=0.0001  <S**2>=2.685
    107A ->109A       -0.47810
    107A ->110A       -0.25919
    107A ->111A       -0.19845
    107A ->115A       -0.13507
    106B ->109B        0.68257
    106B ->110B        0.25246
    106B ->115B        0.16701

 Excited State  15:  2.099-A      4.4134 eV  280.92 nm  f=0.0006  <S**2>=0.851
    107A ->109A        0.64257
    107A ->110A        0.31075
    107A ->111A        0.21974
    107A ->115A        0.12886
    106B ->109B        0.56774
    106B ->110B        0.16823

 Excited State  16:  2.327-A      4.4864 eV  276.36 nm  f=0.0001  <S**2>=1.103
    106A ->109A        0.12929
    106A ->111A        0.19851
    107A ->109A        0.10521
    108A ->109A       -0.25024
```

108A ->110A -0.14983
108A ->111A 0.85957
105B ->109B -0.17249
107B ->109B -0.12073

Excited State 17: 2.963-A 4.4955 eV 275.80 nm f=0.0002 <S**2>=1.946
102A ->109A -0.22309
102A ->110A 0.39444
104A ->110A -0.16575
105A ->109A -0.26622
105A ->110A 0.32719
106A ->109A -0.28415
106A ->110A 0.30348
107A ->109A -0.22276
107A ->110A 0.47987
108A ->111A 0.19173

Excited State 18: 3.206-A 4.5204 eV 274.27 nm f=0.0005 <S**2>=2.319
102A ->109A 0.13233
105A ->109A -0.20585
105A ->110A -0.20635
105A ->111A -0.15028
105A ->115A -0.10654
106A ->109A -0.35237
106A ->110A -0.29444
106A ->111A -0.13285
106A ->115A -0.12527
107A ->110A -0.15080
108A ->111A 0.27194
105B ->109B 0.55849
105B ->110B 0.23213
105B ->115B 0.17262
107B ->109B 0.14397

Excited State 19: 2.187-A 4.6286 eV 267.86 nm f=0.0035 <S**2>=0.946
105A ->109A 0.12479
106A ->112A 0.11507
108A ->112A 0.94504

Excited State 20: 2.896-A 4.6628 eV 265.90 nm f=0.0017 <S**2>=1.847
102A ->110A -0.11738
105A ->110A -0.17506
106A ->109A 0.36324
106A ->110A -0.40779
107A ->109A -0.32935
107A ->110A 0.62173

```
   108A ->110A       -0.14002
    93B ->108B        0.15105
    98B ->108B       -0.13042
   105B ->109B        0.10974

 Excited State  21:  2.776-A      4.6763 eV  265.13 nm  f=0.0017  <S**2>=1.677
   105A ->109A       -0.12072
   106A ->109A        0.16230
   106A ->110A        0.15425
   108A ->112A        0.11892
   105B ->109B        0.23509
   107B ->109B       -0.36025
   107B ->110B        0.80041

 Excited State  22:  2.487-A      4.7231 eV  262.51 nm  f=0.0105  <S**2>=1.297
   105A ->109A       -0.19073
   106A ->109A        0.41147
   106A ->110A        0.26160
   106A ->111A        0.11418
   107A ->110A       -0.20008
   108A ->110A       -0.10292
    93B ->108B        0.21047
    94B ->108B        0.11437
    95B ->108B        0.17067
    96B ->108B        0.14774
    97B ->108B        0.28561
    98B ->108B       -0.29391
   105B ->109B        0.28859
   106B ->109B        0.11770
   107B ->110B       -0.39910
   107B ->112B       -0.16778

 Excited State  23:  2.434-A      4.7630 eV  260.31 nm  f=0.0082  <S**2>=1.231
   105A ->109A        0.38330
   105A ->110A        0.29464
   105A ->111A        0.16388
   105A ->115A        0.12063
   106A ->109A       -0.31866
   106A ->110A       -0.21501
   107A ->109A        0.13059
   108A ->109A        0.15975
   108A ->112A       -0.17102
    93B ->108B        0.30779
    94B ->108B        0.19391
    95B ->108B        0.21383
    96B ->108B        0.10178
```

97B ->108B 0.11272
98B ->108B -0.37237
107B ->110B 0.18315
107B ->112B -0.16624

Excited State 24: 2.743-A 4.8079 eV 257.88 nm f=0.0003 <S**2>=1.631
102A ->110A -0.14162
105A ->109A 0.45178
105A ->110A -0.24830
106A ->109A -0.17327
106A ->110A 0.33212
107A ->109A -0.14885
107A ->110A 0.20713
108A ->110A 0.16319
93B ->108B -0.18086
94B ->108B -0.12115
96B ->108B 0.11054
97B ->108B 0.45343
98B ->108B 0.11727
105B ->109B 0.19474
107B ->112B -0.27387

Excited State 25: 3.177-A 4.8504 eV 255.61 nm f=0.0001 <S**2>=2.273
105A ->110A -0.13305
106A ->110A 0.12615
107A ->110A 0.11650
107A ->111A -0.24609
106B ->109B -0.32499
106B ->110B 0.83661
107B ->112B -0.11739

Excited State 26: 2.708-A 4.8508 eV 255.59 nm f=0.0020 <S**2>=1.584
105A ->109A 0.15342
105A ->110A 0.42838
105A ->111A 0.10820
106A ->109A 0.21585
106A ->110A -0.23297
93B ->108B -0.18782
94B ->108B -0.13774
95B ->108B -0.17331
97B ->108B 0.44940
98B ->108B 0.17287
105B ->109B 0.22893
106B ->110B 0.20441
107B ->112B 0.40643

Excited State 27: 2.206-A 4.8631 eV 254.95 nm f=0.0008 <S**2>=0.967
105A ->109A -0.28918
105A ->110A -0.23515
105A ->111A -0.11005
106A ->109A -0.17212
96B ->108B -0.22571
97B ->108B 0.59699
98B ->108B -0.12297
105B ->109B -0.36027
107B ->111B 0.41353

Excited State 28: 2.754-A 4.8846 eV 253.83 nm f=0.0021 <S**2>=1.646
105A ->109A 0.18568
106A ->110A 0.15286
108A ->113A 0.16864
108A ->114A -0.14076
96B ->108B 0.14806
97B ->108B -0.20463
105B ->109B 0.14655
107B ->111B 0.83579
107B ->112B 0.20541

Excited State 29: 2.345-A 4.8987 eV 253.10 nm f=0.0008 <S**2>=1.125
107A ->109A -0.23447
107A ->110A -0.16999
107A ->111A 0.81421
108A ->113A 0.24010
108A ->114A -0.20435
106B ->110B 0.29601

Excited State 30: 2.201-A 4.9123 eV 252.40 nm f=0.0007 <S**2>=0.961
107A ->111A -0.33227
108A ->113A 0.66063
108A ->114A -0.54669
108A ->115A -0.11527
96B ->108B -0.14921
97B ->108B 0.11070
107B ->111B -0.14061

M06 method:
Excitation energies and oscillator strengths:

Excited State 1: 2.018-A 1.7276 eV 717.67 nm f=0.0000 <S**2>=0.768
104B ->108B -0.24819
105B ->108B 0.61040
106B ->108B 0.73147

```
This state for optimization and/or second-order correction.
Total Energy, E(TD-HF/TD-DFT) =  -2842.01564230
Copying the excited state density for this state as the 1-particle RhoCI density.

Excited State   2:  2.013-A      1.9289 eV  642.79 nm  f=0.0125  <S**2>=0.763
   105B ->108B       -0.54725
   106B ->108B        0.39419
   107B ->108B        0.72458

Excited State   3:  2.014-A      2.1342 eV  580.95 nm  f=0.0396  <S**2>=0.764
   105B ->108B        0.52330
   106B ->108B       -0.50935
   107B ->108B        0.66529

Excited State   4:  2.029-A      2.4779 eV  500.37 nm  f=0.0020  <S**2>=0.779
   100B ->108B       -0.10550
   104B ->108B        0.93723
   105B ->108B        0.18130
   106B ->108B        0.17687
   107B ->108B        0.11755

Excited State   5:  2.013-A      2.7642 eV  448.53 nm  f=0.0008  <S**2>=0.763
   103B ->108B        0.99172

Excited State   6:  2.013-A      2.8155 eV  440.37 nm  f=0.0031  <S**2>=0.763
   102B ->108B        0.99132

Excited State   7:  2.145-A      3.4477 eV  359.61 nm  f=0.0001  <S**2>=0.900
    94B ->108B       -0.10092
    96B ->108B        0.10491
    99B ->108B        0.12411
   100B ->108B        0.66927
   101B ->108B       -0.63069
   104B ->108B        0.15215
   104B ->112B        0.16659
   104B ->113B       -0.10723

Excited State   8:  2.044-A      3.7497 eV  330.65 nm  f=0.0270  <S**2>=0.794
   106A ->110A        0.13559
   108A ->109A       -0.19692
   108A ->110A        0.82099
    93B ->108B       -0.16592
    99B ->108B       -0.30011
   100B ->108B        0.21870
   101B ->108B        0.19980
```

```
Excited State   9:  2.316-A      3.8981 eV  318.06 nm  f=0.0003  <S**2>=1.091
   106A ->109A       -0.18407
   108A ->109A        0.89915
   108A ->110A        0.15937
   108A ->111A        0.13925
   108A ->115A        0.14089
   107B ->109B        0.12345

Excited State  10:  2.086-A      4.1040 eV  302.11 nm  f=0.0255  <S**2>=0.838
    99A ->110A        0.12729
   108A ->110A        0.40073
    93B ->108B       -0.14186
    99B ->108B        0.55188
   100B ->108B       -0.51443
   101B ->108B       -0.43435

Excited State  11:  2.109-A      4.2015 eV  295.10 nm  f=0.0006  <S**2>=0.862
    93B ->108B       -0.19965
    94B ->108B        0.72555
    95B ->108B        0.38136
    96B ->108B       -0.12895
    98B ->108B        0.31141
    99B ->108B       -0.14672
   101B ->108B       -0.22834
   107B ->109B        0.13513

Excited State  12:  3.135-A      4.2331 eV  292.89 nm  f=0.0002  <S**2>=2.207
   107A ->109A       -0.34409
   108A ->109A       -0.17530
   106B ->109B        0.28156
   107B ->109B        0.75228
   107B ->110B        0.14956
   107B ->115B        0.14166
   107B ->122B        0.10003

Excited State  13:  3.220-A      4.3280 eV  286.47 nm  f=0.0001  <S**2>=2.342
   106A ->109A       -0.12523
   107A ->109A        0.53989
   107A ->111A        0.10581
   107A ->115A        0.10911
   105B ->109B        0.12984
   106B ->109B       -0.53341
   106B ->110B       -0.10998
   106B ->115B       -0.11152
   107B ->109B        0.46896
```

Excited State 14: 2.047-A 4.4227 eV 280.34 nm f=0.0010 <S**2>=0.798
93B ->108B -0.15284
94B ->108B 0.15433
95B ->108B 0.13273
99B ->108B 0.69720
100B ->108B 0.38616
101B ->108B 0.52134

Excited State 15: 2.102-A 4.4663 eV 277.60 nm f=0.0008 <S**2>=0.855
107A ->109A 0.63573
107A ->111A 0.10857
107A ->115A 0.10092
106B ->109B 0.68090
106B ->110B 0.12019
106B ->115B 0.10728

Excited State 16: 3.331-A 4.5958 eV 269.78 nm f=0.0002 <S**2>=2.524
104A ->109A -0.14017
105A ->109A -0.39406
105A ->115A -0.11026
106A ->109A 0.38086
107A ->109A -0.13915
104B ->109B 0.17016
105B ->109B 0.62399
105B ->110B 0.13768
105B ->115B 0.15189
105B ->119B -0.10876
105B ->122B 0.10543

Excited State 17: 2.922-A 4.7142 eV 263.00 nm f=0.0000 <S**2>=1.885
103A ->110A 0.17126
104A ->110A -0.50159
105A ->110A 0.54457
106A ->109A 0.25718
106A ->110A -0.28915
107A ->110A 0.40172
105B ->109B -0.12722

Excited State 18: 2.606-A 4.7283 eV 262.22 nm f=0.0007 <S**2>=1.447
104A ->110A 0.19079
105A ->109A 0.35827
105A ->110A -0.17779
106A ->109A 0.68248
106A ->110A 0.12842
106A ->111A 0.14188
106A ->115A 0.11047

```
   107A ->110A      -0.15767
   108A ->109A       0.20910
   108A ->111A      -0.16879
   108A ->112A       0.13855
   105B ->109B      -0.15313
   106B ->109B      -0.11348
   107B ->109B       0.15552

Excited State  19:  2.089-A      4.8118 eV  257.67 nm  f=0.0267  <S**2>=0.841
   105A ->109A      -0.24783
   106A ->109A       0.13711
   108A ->110A       0.20668
   108A ->111A       0.23231
   108A ->112A      -0.32323
    92B ->108B       0.13196
    93B ->108B       0.49290
    94B ->108B       0.15167
    95B ->108B      -0.28091
    97B ->108B      -0.11400
    98B ->108B       0.41194
    99B ->108B       0.10743
   105B ->109B      -0.21693

Excited State  20:  2.215-A      4.8364 eV  256.35 nm  f=0.0029  <S**2>=0.977
   105A ->109A       0.11707
   106A ->109A       0.22233
   106A ->111A      -0.16146
   108A ->111A       0.90186
    93B ->108B      -0.16318
    98B ->108B      -0.12093

Excited State  21:  2.183-A      4.9046 eV  252.79 nm  f=0.0029  <S**2>=0.942
   105A ->109A      -0.16552
   106A ->112A      -0.13786
   108A ->109A      -0.10820
   108A ->112A       0.88012
    93B ->108B       0.11852
    95B ->108B      -0.10222
    98B ->108B       0.17962
   105B ->109B      -0.11890

Excited State  22:  2.094-A      4.9503 eV  250.46 nm  f=0.0060  <S**2>=0.846
   104A ->109A       0.12154
   105A ->109A       0.51022
   106A ->109A      -0.12607
   106A ->110A      -0.13735
```

```
    108A ->110A        0.16948
     93B ->108B        0.36504
     94B ->108B        0.11701
     96B ->108B        0.16342
     97B ->108B        0.38227
    105B ->109B        0.45171

 Excited State  23:  2.088-A      5.0088 eV  247.53 nm  f=0.0011  <S**2>=0.840
    103A ->109A       -0.11236
    105A ->109A       -0.27898
    106A ->109A        0.13908
    106A ->110A       -0.11265
    107A ->110A       -0.14151
     97B ->108B        0.79856
     98B ->108B       -0.25809
    105B ->109B       -0.26520

 Excited State  24:  2.900-A      5.0513 eV  245.45 nm  f=0.0029  <S**2>=1.852
    100A ->110A        0.10737
    105A ->110A       -0.23701
    106A ->110A        0.41949
    107A ->110A        0.75916
     95B ->108B       -0.17202
     96B ->108B       -0.10642
     97B ->108B        0.22176

 Excited State  25:  3.433-A      5.0781 eV  244.15 nm  f=0.0000  <S**2>=2.697
    102A ->109A        0.12103
    103A ->109A       -0.51762
    103A ->111A       -0.12113
    103A ->115A       -0.14297
    103A ->119A        0.10186
    103A ->122A       -0.10541
    104A ->109A       -0.16869
    103B ->109B        0.64942
    103B ->110B        0.15585
    103B ->115B        0.16986
    103B ->119B       -0.11849
    103B ->122B        0.11690

 Excited State  26:  3.432-A      5.0822 eV  243.96 nm  f=0.0001  <S**2>=2.695
    102A ->109A       -0.55138
    102A ->111A       -0.12970
    102A ->115A       -0.15307
    102A ->119A        0.10690
    102A ->122A       -0.11237
```

```
   103A ->109A       -0.10559
   104A ->109A       -0.11464
   102B ->109B        0.62975
   102B ->110B        0.15157
   102B ->115B        0.16670
   102B ->119B       -0.11491
   102B ->122B        0.11496

Excited State  27:  2.524-A      5.1382 eV  241.30 nm  f=0.0002  <S**2>=1.343
   105A ->109A       -0.10688
   105A ->110A       -0.17377
   106A ->109A        0.10020
   106A ->110A       -0.18165
   107A ->110A        0.20141
   108A ->113A        0.22985
   108A ->114A        0.42385
    95B ->108B        0.24414
    96B ->108B        0.41081
    98B ->108B       -0.11887
   103B ->109B       -0.11509
   107B ->109B       -0.12063
   107B ->110B        0.50278

Excited State  28:  2.660-A      5.1470 eV  240.89 nm  f=0.0006  <S**2>=1.519
   105A ->110A        0.11884
   106A ->110A        0.15213
   107A ->110A       -0.13936
    93B ->108B       -0.10249
    95B ->108B       -0.22387
    96B ->108B       -0.41508
    98B ->108B        0.10031
   107B ->109B       -0.17810
   107B ->110B        0.77324

Excited State  29:  2.244-A      5.1657 eV  240.01 nm  f=0.0003  <S**2>=1.009
   105A ->110A        0.13589
   106A ->110A        0.20875
   107A ->110A       -0.13386
   108A ->113A        0.51283
   108A ->114A        0.63049
   108A ->118A        0.10167
    95B ->108B       -0.13471
    96B ->108B       -0.27786
   107B ->110B       -0.23118

Excited State  30:  2.819-A      5.2092 eV  238.01 nm  f=0.0041  <S**2>=1.737
```

103A ->109A -0.13615
104A ->110A -0.13722
105A ->110A 0.32063
106A ->110A 0.60444
107A ->110A -0.19920
93B ->108B 0.10435
94B ->108B 0.12152
96B ->108B 0.47010
98B ->108B -0.11316
107B ->111B -0.28484
107B ->112B 0.11491
107B ->113B -0.15447

[Cu(II)LAsc], conformer 2

Excitation energies and oscillator strengths:

Excited State 1: 2.025-A 1.5942 eV 777.72 nm f=0.0000 <S**2>=0.775
101B ->108B -0.12179
104B ->108B 0.63764
105B ->108B 0.16356
106B ->108B -0.28767
107B ->108B 0.67243
This state for optimization and/or second-order correction.
Total Energy, E(TD-HF/TD-DFT) = -2842.83659604
Copying the excited state density for this state as the 1-particle RhoCI density.

Excited State 2: 2.036-A 2.0810 eV 595.80 nm f=0.0114 <S**2>=0.786
104B ->108B -0.18192
105B ->108B -0.10902
106B ->108B 0.80465
107B ->108B 0.54459

Excited State 3: 2.040-A 2.1714 eV 570.98 nm f=0.0008 <S**2>=0.791
104B ->108B 0.63262
105B ->108B 0.32532
106B ->108B 0.50626
107B ->108B -0.46608

Excited State 4: 2.034-A 2.4320 eV 509.80 nm f=0.0002 <S**2>=0.784
104B ->108B -0.36006
105B ->108B 0.91790
107B ->108B 0.11465

Excited State 5: 2.030-A 2.8462 eV 435.62 nm f=0.0001 <S**2>=0.780
102B ->108B 0.12693
103B ->108B 0.98605

```
Excited State   6:  2.030-A      2.8576 eV  433.88 nm  f=0.0016  <S**2>=0.780
   102B ->108B         0.98680
   103B ->108B        -0.12843

Excited State   7:  2.189-A      3.1693 eV  391.20 nm  f=0.0000  <S**2>=0.948
   104A ->109A        -0.13598
   100B ->108B         0.34025
   101B ->108B         0.85778
   104B ->110B        -0.22336
   107B ->108B         0.11064

Excited State   8:  2.045-A      3.3878 eV  365.97 nm  f=0.0750  <S**2>=0.796
   108A ->109A         0.94528
    96B ->108B         0.17367
    99B ->108B        -0.17641

Excited State   9:  2.723-A      3.8223 eV  324.37 nm  f=0.0001  <S**2>=1.603
   106A ->110A        -0.37355
   106A ->111A        -0.10481
   107A ->110A        -0.15798
   108A ->110A         0.80653
   108A ->111A         0.14850
   106B ->109B         0.20439
   107B ->109B         0.16812

Excited State  10:  3.403-A      3.9292 eV  315.55 nm  f=0.0000  <S**2>=2.645
   106A ->110A         0.12618
   107A ->109A         0.17178
   107A ->110A        -0.56847
   107A ->111A        -0.15391
   107A ->115A         0.11942
   108A ->110A        -0.16221
   106B ->109B        -0.17895
   107B ->109B         0.62834
   107B ->111B         0.16494
   107B ->115B        -0.12518

Excited State  11:  2.966-A      3.9572 eV  313.31 nm  f=0.0002  <S**2>=1.950
   104A ->109A         0.47233
   105A ->109A         0.15182
   107A ->109A         0.80656
   107A ->110A         0.10130
   101B ->108B         0.13738
   107B ->109B        -0.14498
```

Excited State 12: 2.855-A 4.0004 eV 309.93 nm f=0.0004 <S**2>=1.788
106A ->110A -0.18486
107A ->110A 0.17846
108A ->110A -0.37587
106B ->109B 0.73217
106B ->111B 0.18999
106B ->114B 0.10102
106B ->115B -0.14263
107B ->109B 0.32436

Excited State 13: 2.046-A 4.0764 eV 304.15 nm f=0.0004 <S**2>=0.796
107A ->110A 0.67756
107A ->111A 0.14923
108A ->110A 0.10847
106B ->109B -0.34668
107B ->109B 0.54423
107B ->111B 0.11363

Excited State 14: 3.031-A 4.1558 eV 298.34 nm f=0.0086 <S**2>=2.046
104A ->109A -0.13993
106A ->109A 0.90443
107A ->109A 0.21437
105B ->109B -0.12090
106B ->109B -0.10000
106B ->110B 0.15723

Excited State 15: 2.267-A 4.2160 eV 294.08 nm f=0.0010 <S**2>=1.035
106A ->110A 0.78183
106A ->111A 0.18815
106A ->115A -0.12345
108A ->110A 0.33671
105B ->109B 0.15912
106B ->109B 0.33707

Excited State 16: 2.919-A 4.2600 eV 291.05 nm f=0.0001 <S**2>=1.880
104A ->109A 0.65160
105A ->109A 0.36163
106A ->109A 0.25168
107A ->109A -0.49838
101B ->108B 0.16112
104B ->110B 0.17101
107B ->110B 0.15165

Excited State 17: 3.414-A 4.2825 eV 289.51 nm f=0.0002 <S**2>=2.664
105A ->110A 0.61365
105A ->111A 0.19236

```
   105A ->114A        0.10651
   105A ->115A       -0.16533
   106A ->109A       -0.12554
   106A ->110A        0.18032
   104B ->109B        0.11789
   105B ->109B       -0.57077
   105B ->111B       -0.18087
   105B ->114B       -0.10024
   105B ->115B        0.15447

Excited State  18:  2.032-A      4.3503 eV  285.00 nm  f=0.0139  <S**2>=0.783
   108A ->109A       -0.10314
    99B ->108B       -0.10545
   100B ->108B        0.90790
   101B ->108B       -0.34953

Excited State  19:  2.259-A      4.4736 eV  277.15 nm  f=0.0002  <S**2>=1.026
   106A ->110A        0.16042
   106A ->111A       -0.22944
   108A ->110A       -0.14154
   108A ->111A        0.93645

Excited State  20:  2.736-A      4.4836 eV  276.53 nm  f=0.0001  <S**2>=1.622
   105A ->109A        0.67463
   104B ->110B       -0.31347
   105B ->110B       -0.10040
   106B ->110B        0.22515
   107B ->110B       -0.56867

Excited State  21:  2.774-A      4.5028 eV  275.35 nm  f=0.0013  <S**2>=1.674
   104A ->109A       -0.47123
   105A ->109A        0.59256
   104B ->110B        0.20855
   105B ->110B        0.11814
   106B ->110B       -0.14940
   107B ->110B        0.51762

Excited State  22:  2.126-A      4.5469 eV  272.68 nm  f=0.0767  <S**2>=0.880
   106A ->109A       -0.10072
   108A ->109A       -0.19459
   108A ->112A        0.57442
    94B ->108B       -0.23948
    96B ->108B        0.40082
    99B ->108B       -0.50582
   100B ->108B       -0.13979
   107B ->110B       -0.12700
```

```
Excited State  23:  2.367-A      4.6094 eV  268.98 nm  f=0.0418  <S**2>=1.150
   105A ->110A        0.13791
   106A ->112A       -0.20833
   108A ->109A        0.10244
   108A ->112A        0.67851
    94B ->108B        0.17253
    96B ->108B       -0.23732
    99B ->108B        0.36090
   105B ->109B        0.18974
   106B ->110B        0.32304
   107B ->110B        0.16512

Excited State  24:  2.268-A      4.6180 eV  268.48 nm  f=0.0000  <S**2>=1.036
   105A ->110A        0.38615
   106A ->109A       -0.10646
   106A ->112A        0.10577
   108A ->112A       -0.31837
    94B ->108B       -0.17271
    96B ->108B        0.18722
    98B ->108B       -0.10831
    99B ->108B       -0.13468
   105B ->109B        0.41396
   106B ->110B        0.57061
   107B ->110B        0.19519

Excited State  25:  3.206-A      4.6653 eV  265.76 nm  f=0.0002  <S**2>=2.320
   107A ->110A        0.12856
   107A ->111A       -0.29710
   107B ->109B       -0.27148
   107B ->111B        0.87251

Excited State  26:  3.459-A      4.6804 eV  264.90 nm  f=0.0001  <S**2>=2.741
   103A ->110A        0.61894
   103A ->111A        0.19749
   103A ->114A        0.10744
   103A ->115A       -0.16404
   103B ->109B       -0.60261
   103B ->111B       -0.19403
   103B ->114B       -0.10582
   103B ->115B        0.16187

Excited State  27:  3.456-A      4.6911 eV  264.30 nm  f=0.0000  <S**2>=2.736
   102A ->110A       -0.59472
   102A ->111A       -0.19040
   102A ->114A       -0.10428
```

```
   102A ->115A        0.15949
   102B ->109B        0.62517
   102B ->111B        0.19967
   102B ->114B        0.10842
   102B ->115B       -0.16556

 Excited State  28:  2.747-A      4.7042 eV  263.56 nm  f=0.0018  <S**2>=1.637
   106A ->111A       -0.10918
   107A ->110A       -0.22373
   107A ->111A        0.77511
   106B ->109B       -0.14466
   106B ->111B        0.41700
   107B ->111B        0.25438

 Excited State  29:  2.564-A      4.7229 eV  262.52 nm  f=0.0016  <S**2>=1.394
   107A ->111A       -0.41505
   106B ->109B       -0.23801
   106B ->111B        0.80070
   107B ->111B       -0.21819

 Excited State  30:  2.272-A      4.7336 eV  261.93 nm  f=0.0067  <S**2>=1.041
   103A ->110A       -0.17773
   105A ->110A       -0.46759
   106A ->109A       -0.15858
   108A ->112A       -0.11888
   103B ->109B       -0.23415
   105B ->109B       -0.43740
   106B ->110B        0.47432
   106B ->111B        0.20294
   107B ->110B        0.26743
```

[Cu(II)LAsc], conformer 3

Excitation energies and oscillator strengths:

```
 Excited State   1:  2.024-A      1.6708 eV  742.07 nm  f=0.0008  <S**2>=0.774
   102B ->108B        0.59646
   105B ->108B        0.24979
   107B ->108B        0.74872
 This state for optimization and/or second-order correction.
 Total Energy, E(TD-HF/TD-DFT) =  -2842.85251361
 Copying the excited state density for this state as the 1-particle RhoCI density.

 Excited State   2:  2.025-A      1.7957 eV  690.44 nm  f=0.0017  <S**2>=0.775
   102B ->108B       -0.40235
   105B ->108B       -0.17466
   106B ->108B        0.77605
```

```
    107B ->108B        0.43756

 Excited State   3:  2.025-A      1.8319 eV  676.81 nm  f=0.0055  <S**2>=0.776
    102B ->108B        0.53955
    105B ->108B        0.23229
    106B ->108B        0.62573
    107B ->108B       -0.49338

 Excited State   4:  2.028-A      2.4726 eV  501.44 nm  f=0.0001  <S**2>=0.778
    102B ->108B       -0.38049
    105B ->108B        0.92074

 Excited State   5:  2.029-A      2.6440 eV  468.92 nm  f=0.0054  <S**2>=0.779
    104B ->108B        0.99053

 Excited State   6:  2.027-A      2.6654 eV  465.15 nm  f=0.0002  <S**2>=0.778
    103B ->108B        0.99386

 Excited State   7:  2.238-A      3.2406 eV  382.59 nm  f=0.0070  <S**2>=1.002
    102A ->109A       -0.15226
    108A ->109A        0.32003
     96B ->108B        0.17525
     98B ->108B       -0.15226
     99B ->108B        0.66401
    100B ->108B       -0.50792
    102B ->109B        0.22755

 Excited State   8:  2.098-A      3.2785 eV  378.17 nm  f=0.0679  <S**2>=0.851
    106A ->109A       -0.34936
    107A ->109A        0.23335
    108A ->109A        0.81087
     94B ->108B        0.15108
     99B ->108B       -0.23074
    100B ->108B        0.18462

 Excited State   9:  2.828-A      3.4854 eV  355.72 nm  f=0.0028  <S**2>=1.750
    106A ->109A       -0.14654
    107A ->109A        0.92892
    108A ->109A       -0.30632

 Excited State  10:  2.858-A      3.5886 eV  345.49 nm  f=0.0081  <S**2>=1.792
    106A ->109A        0.89044
    107A ->109A        0.26212
    108A ->109A        0.33715

 Excited State  11:  3.443-A      3.7116 eV  334.05 nm  f=0.0001  <S**2>=2.713
```

```
   106A ->110A        0.30777
   107A ->110A       -0.27332
   108A ->110A        0.50887
   106B ->110B       -0.32160
   107B ->109B       -0.15269
   107B ->110B        0.55502

Excited State  12:  3.444-A      3.8316 eV  323.58 nm  f=0.0001  <S**2>=2.716
   106A ->110A        0.27419
   107A ->110A        0.57704
   108A ->110A        0.14176
   106B ->109B        0.16156
   106B ->110B       -0.56179
   107B ->109B        0.12380
   107B ->110B       -0.32485

Excited State  13:  2.269-A      4.0285 eV  307.77 nm  f=0.0003  <S**2>=1.037
   102A ->109A        0.16792
   105A ->109A        0.16268
   106A ->110A        0.13679
   107A ->110A        0.61250
   106B ->109B       -0.19022
   106B ->110B        0.32526
   107B ->109B       -0.46111
   107B ->110B        0.36055

Excited State  14:  2.772-A      4.0578 eV  305.55 nm  f=0.0005  <S**2>=1.670
   102A ->109A        0.51627
   105A ->109A        0.55022
   106A ->110A       -0.10572
   107A ->110A       -0.25000
    95B ->108B       -0.22113
    99B ->108B        0.13262
   102B ->109B       -0.14737
   106B ->109B        0.18317
   106B ->110B       -0.21235
   107B ->109B       -0.28878
   107B ->110B       -0.22258

Excited State  15:  2.787-A      4.0934 eV  302.89 nm  f=0.0029  <S**2>=1.691
   102A ->109A        0.28249
   105A ->109A        0.42142
   106A ->110A        0.12343
   108A ->110A        0.17114
   106B ->109B       -0.26480
   106B ->110B        0.18156
```

```
  107B ->109B        0.74020
  107B ->110B        0.13850

 Excited State  16:  2.614-A      4.1387 eV  299.57 nm  f=0.0034  <S**2>=1.458
  106A ->110A       -0.12846
  107A ->110A        0.14361
  108A ->110A       -0.29331
  106B ->109B        0.78731
  107B ->109B        0.23904
  107B ->110B        0.40071

 Excited State  17:  2.239-A      4.2096 eV  294.52 nm  f=0.0081  <S**2>=1.003
  104A ->109A       -0.10272
  105A ->110A        0.11703
  106A ->110A        0.22750
  108A ->110A        0.50587
  106B ->109B        0.42926
  106B ->110B        0.53590
  107B ->110B       -0.34247

 Excited State  18:  2.775-A      4.2370 eV  292.62 nm  f=0.0001  <S**2>=1.676
  102A ->109A       -0.43495
  105A ->109A        0.63136
   95B ->108B        0.46663
   95B ->109B       -0.11825
   97B ->108B        0.10456
  102B ->109B        0.22975
  107B ->109B       -0.18279

 Excited State  19:  2.335-A      4.3738 eV  283.47 nm  f=0.0002  <S**2>=1.113
  102A ->109A        0.54210
  103A ->109A        0.18896
  104A ->109A        0.20905
  105A ->109A       -0.25306
   95B ->108B        0.61028
   95B ->109B       -0.13509
   96B ->108B       -0.13269
   97B ->108B        0.14427
   98B ->108B       -0.17498
  100B ->108B       -0.19366
  102B ->109B        0.16771

 Excited State  20:  2.840-A      4.3992 eV  281.83 nm  f=0.0018  <S**2>=1.766
  102A ->109A       -0.20831
  103A ->109A        0.33145
  104A ->109A        0.86967
```

```
   95B ->108B      -0.12906
  101B ->108B       0.10762

Excited State  21:  2.845-A      4.4118 eV  281.03 nm  f=0.0001  <S**2>=1.773
  102A ->109A      -0.11527
  103A ->109A       0.90232
  104A ->109A      -0.39748

Excited State  22:  3.437-A      4.4371 eV  279.43 nm  f=0.0000  <S**2>=2.703
  105A ->110A       0.63879
  105A ->115A       0.10259
  102B ->110B       0.10097
  105B ->109B       0.19250
  105B ->110B      -0.62731
  105B ->115B      -0.10784

Excited State  23:  2.047-A      4.4811 eV  276.68 nm  f=0.0018  <S**2>=0.797
  101B ->108B       0.98122

Excited State  24:  2.696-A      4.5142 eV  274.66 nm  f=0.0018  <S**2>=1.567
  103A ->110A      -0.29114
  104A ->110A      -0.33400
  106A ->110A       0.63546
  107A ->110A      -0.17313
  108A ->110A      -0.41830
  104B ->110B       0.31306

Excited State  25:  2.035-A      4.5758 eV  270.96 nm  f=0.0022  <S**2>=0.785
   94B ->108B       0.13358
   95B ->108B       0.13469
   98B ->108B      -0.16254
   99B ->108B       0.54589
  100B ->108B       0.77740

Excited State  26:  3.452-A      4.5900 eV  270.12 nm  f=0.0000  <S**2>=2.728
  103A ->110A       0.47282
  104A ->110A      -0.44387
  103B ->109B      -0.16437
  103B ->110B       0.63326
  103B ->115B       0.11293

Excited State  27:  2.104-A      4.6389 eV  267.27 nm  f=0.0410  <S**2>=0.857
  106A ->109A       0.11820
  106A ->122A       0.11905
  108A ->109A      -0.11144
  108A ->122A      -0.12388
```

```
    94B ->108B        0.75653
    96B ->108B        0.41448
    97B ->108B        0.16716
    99B ->108B       -0.14213
   100B ->108B       -0.11649

 Excited State  28:  2.909-A      4.6522 eV  266.51 nm  f=0.0009  <S**2>=1.865
   103A ->110A       -0.24034
   104A ->110A       -0.23450
   105A ->110A       -0.15961
   106A ->110A       -0.41820
   107A ->110A        0.11673
   108A ->110A        0.27660
   103B ->110B       -0.10262
   104B ->109B       -0.19070
   104B ->110B        0.64329
   104B ->115B        0.10308
   105B ->109B        0.12076
   105B ->110B       -0.15679

 Excited State  29:  2.626-A      4.6857 eV  264.60 nm  f=0.0022  <S**2>=1.474
   102A ->109A        0.12360
   105A ->110A       -0.21380
   106A ->110A        0.10151
    95B ->108B       -0.18494
    97B ->108B       -0.15041
    98B ->108B        0.16873
    99B ->108B       -0.14466
   102B ->109B        0.41623
   105B ->109B        0.77016

 Excited State  30:  2.054-A      4.7551 eV  260.74 nm  f=0.0013  <S**2>=0.804
   103A ->110A       -0.14092
   105A ->110A        0.38522
   106A ->110A       -0.14324
    95B ->108B        0.10948
    98B ->108B        0.73926
    99B ->108B        0.15202
   102B ->109B        0.15041
   105B ->110B        0.37794
```

[Cu(II)LHAsc]$^{+}$

```
Excited State   1:  2.009-A      0.8135 eV 1524.17 nm  f=0.0031  <S**2>=0.759
    85B ->108B        0.10397
    92B ->108B        0.16526
    93B ->108B        0.34975
```

95B ->108B 0.13350
96B ->108B -0.12554
97B ->108B 0.18261
106B ->108B 0.83092
107B ->108B 0.19056
This state for optimization and/or second-order correction.
Total Energy, E(TD-HF/TD-DFT) = -2843.02524431
Copying the excited state density for this state as the 1-particle RhoCI density.

Excited State 2: 2.009-A 1.4749 eV 840.63 nm f=0.0029 <S**2>=0.759
84B ->108B 0.10090
85B ->108B 0.14046
87B ->108B 0.24844
88B ->108B -0.20399
89B ->108B 0.23946
91B ->108B -0.13057
92B ->108B 0.16194
93B ->108B -0.19125
97B ->108B -0.14140
98B ->108B 0.29906
100B ->108B -0.42193
102B ->108B -0.10283
105B ->108B 0.59164
107B ->108B -0.13454

Excited State 3: 2.008-A 1.8832 eV 658.36 nm f=0.0026 <S**2>=0.758
84B ->108B -0.17206
89B ->108B 0.17081
92B ->108B -0.34941
93B ->108B 0.45283
95B ->108B 0.14866
96B ->108B 0.22604
97B ->108B -0.29847
98B ->108B 0.55824
99B ->108B 0.16632
105B ->108B -0.10380
107B ->108B -0.11931

Excited State 4: 2.029-A 1.9063 eV 650.41 nm f=0.0786 <S**2>=0.779
89B ->108B 0.16503
93B ->108B 0.11002
94B ->108B -0.20692
95B ->108B 0.15609
97B ->108B -0.47005
98B ->108B -0.40364
99B ->108B 0.11274

```
  106B ->108B       -0.11019
  107B ->108B        0.64622

Excited State   5:  2.037-A      2.0082 eV  617.40 nm  f=0.0965  <S**2>=0.788
   93B ->108B       -0.11196
   94B ->108B        0.15585
   95B ->108B       -0.15046
   97B ->108B        0.38037
   98B ->108B        0.50590
  106B ->108B       -0.13989
  107B ->108B        0.69032

Excited State   6:  2.178-A      3.3295 eV  372.38 nm  f=0.0055  <S**2>=0.936
  108A ->109A       -0.23773
   84B ->108B       -0.20985
   85B ->108B       -0.23088
   87B ->108B       -0.30837
   88B ->108B        0.24216
   89B ->108B       -0.24560
   91B ->108B        0.12606
   92B ->108B       -0.19420
   93B ->108B        0.12526
   98B ->108B       -0.16712
  102B ->108B        0.11257
  105B ->108B        0.64285
  107B ->109B       -0.19077

Excited State   7:  3.149-A      3.3952 eV  365.17 nm  f=0.0227  <S**2>=2.228
  106A ->109A       -0.13564
  108A ->109A        0.67737
   84B ->108B       -0.11323
   85B ->108B       -0.10223
  105B ->108B        0.22969
  107B ->108B        0.10398
  107B ->109B        0.58197

Excited State   8:  2.030-A      3.8184 eV  324.70 nm  f=0.0267  <S**2>=0.780
   84B ->108B        0.10607
   85B ->108B        0.14529
   87B ->108B        0.10219
   92B ->108B        0.18589
   93B ->108B        0.63706
   95B ->108B        0.21915
   96B ->108B       -0.18236
   97B ->108B        0.28318
  102B ->108B        0.14307
```

```
 105B ->108B        0.10514
 106B ->108B       -0.50312

Excited State   9:  2.024-A      4.0076 eV  309.37 nm  f=0.0344  <S**2>=0.774
  93B ->108B       -0.12354
  99B ->108B        0.19886
 101B ->108B       -0.14453
 102B ->108B        0.93158

Excited State  10:  2.038-A      4.4182 eV  280.62 nm  f=0.0225  <S**2>=0.789
  97B ->108B        0.12917
  99B ->108B        0.90116
 101B ->108B       -0.21262
 102B ->108B       -0.19125
 107B ->109B       -0.12825

Excited State  11:  2.405-A      4.9484 eV  250.55 nm  f=0.0019  <S**2>=1.196
 101A ->109A       -0.11585
 105A ->109A       -0.27960
  84B ->108B        0.10822
  85B ->108B        0.10145
  87B ->108B        0.10713
 100B ->108B        0.73031
 100B ->109B        0.16477
 101B ->108B        0.16503
 104B ->108B       -0.17690
 105B ->108B        0.29575
 105B ->109B        0.25440

Excited State  12:  3.177-A      5.0324 eV  246.37 nm  f=0.0009  <S**2>=2.273
 101A ->109A        0.18987
 104A ->109A       -0.10522
 105A ->109A        0.62048
 106A ->109A       -0.22393
 107A ->109A       -0.20487
 100B ->108B        0.37248
 100B ->109B       -0.11505
 105B ->108B        0.19072
 105B ->109B       -0.42922
 106B ->109B       -0.11826

Excited State  13:  2.080-A      5.2578 eV  235.81 nm  f=0.1888  <S**2>=0.832
 108A ->109A       -0.60151
 105B ->109B       -0.18989
 107B ->109B        0.71201
```

Excited State 14: 2.060-A   5.4278 eV 228.43 nm f=0.0013 <S**2>=0.811
105A ->109A   -0.11345
89B ->108B   0.17377
96B ->108B   0.10214
97B ->108B   0.11217
99B ->108B   -0.15030
100B ->108B   0.22808
101B ->108B   -0.56139
103B ->108B   -0.13897
104B ->108B   0.65090
105B ->109B   -0.15951

Excited State 15: 2.062-A   5.4741 eV 226.49 nm f=0.0209 <S**2>=0.813
101A ->109A   0.11759
105A ->109A   0.45929
106A ->109A   -0.15686
107A ->109A   -0.16607
108A ->109A   -0.16931
100B ->109B   0.17901
101B ->108B   -0.19286
104B ->108B   0.11573
105B ->109B   0.68323
106B ->109B   0.20274
107B ->109B   0.18763

Excited State 16: 2.177-A   5.6025 eV 221.30 nm f=0.0013 <S**2>=0.935
102A ->109A   -0.17212
84B ->108B   0.12697
89B ->108B   -0.13515
96B ->108B   -0.10195
99B ->108B   0.10779
101B ->108B   0.56847
101B ->109B   0.12568
102B ->108B   0.11176
104B ->108B   0.68204
104B ->109B   -0.12843

Excited State 17: 2.836-A   5.7532 eV 215.51 nm f=0.0088 <S**2>=1.760
99A ->109A   -0.12069
102A ->109A   0.42020
108A ->109A   -0.10786
84B ->108B   -0.14852
89B ->108B   0.30690
95B ->108B   -0.26658
96B ->109B   0.10474
97B ->108B   0.17082

```
    98B ->108B       -0.11527
   100B ->109B        0.11475
   101B ->108B        0.32058
   101B ->109B       -0.41073
   103B ->108B        0.27806
   104B ->108B        0.10419

 Excited State  18:  2.804-A      5.8098 eV  213.41 nm  f=0.0007  <S**2>=1.716
   108A ->110A        0.86619
   108A ->111A        0.22519
   107B ->110B        0.25593

 Excited State  19:  2.683-A      5.8589 eV  211.62 nm  f=0.0049  <S**2>=1.549
    99A ->109A       -0.11705
   102A ->109A        0.41148
   108A ->110A       -0.21879
    84B ->108B        0.16244
    87B ->108B       -0.10089
    89B ->108B       -0.34596
    94B ->108B        0.14137
    95B ->108B        0.39555
    96B ->108B       -0.10639
    96B ->109B        0.10549
    97B ->108B       -0.19391
    98B ->108B        0.12917
   101B ->109B       -0.35083
   103B ->108B       -0.24703

 Excited State  20:  2.603-A      5.9585 eV  208.08 nm  f=0.0042  <S**2>=1.444
   108A ->110A       -0.23905
    95B ->108B       -0.14332
   107B ->110B        0.89320
   107B ->111B        0.14205
   107B ->116B        0.10878

 Excited State  21:  2.053-A      5.9806 eV  207.31 nm  f=0.0015  <S**2>=0.804
    89B ->108B        0.17675
    92B ->108B       -0.30283
    93B ->108B       -0.25760
    95B ->108B        0.64289
    96B ->108B       -0.13058
    97B ->108B        0.23539
   103B ->108B        0.50998
   107B ->110B        0.10109

 Excited State  22:  2.031-A      6.0108 eV  206.27 nm  f=0.0011  <S**2>=0.781
```

92B ->108B 0.11021
94B ->108B 0.84634
96B ->108B 0.27510
97B ->108B -0.26364
103B ->108B 0.27609

Excited State 23: 2.053-A 6.1428 eV 201.84 nm f=0.0035 <S**2>=0.804
84B ->108B 0.15275
86B ->108B -0.12447
89B ->108B -0.38280
93B ->108B 0.11055
94B ->108B -0.30146
95B ->108B -0.19654
97B ->108B -0.13731
98B ->108B 0.14369
100B ->108B 0.15668
101B ->108B -0.25469
103B ->108B 0.67072

Excited State 24: 3.321-A 6.2671 eV 197.83 nm f=0.0002 <S**2>=2.508
101A ->109A 0.58254
104A ->109A -0.10362
105A ->109A -0.13057
107A ->109A 0.33535
90B ->108B -0.20220
92B ->108B 0.14634
100B ->109B -0.42891
106B ->109B 0.28877
106B ->110B 0.10409

Excited State 25: 2.120-A 6.3116 eV 196.44 nm f=0.0016 <S**2>=0.873
101A ->109A 0.15993
84B ->108B -0.20315
87B ->108B 0.12583
89B ->108B -0.14957
90B ->108B 0.91070

Excited State 26: 2.718-A 6.4661 eV 191.74 nm f=0.0005 <S**2>=1.597
101A ->109A -0.40535
107A ->109A -0.21506
107A ->110A 0.16282
93B ->110B 0.10707
100B ->109B -0.27624
106B ->109B 0.38022
106B ->110B 0.58913
106B ->116B 0.10563

106B ->118B -0.11880

Excited State 27: 2.536-A 6.5126 eV 190.38 nm f=0.0002 <S**2>=1.358
101A ->109A -0.24654
107A ->109A -0.20046
107A ->110A -0.23038
86B ->108B 0.11592
88B ->108B 0.45810
90B ->108B 0.11320
91B ->108B -0.11416
92B ->108B 0.26567
93B ->108B -0.12397
97B ->108B -0.12077
100B ->109B -0.32779
106B ->109B 0.12119
106B ->110B -0.43733

Excited State 28: 2.272-A 6.5843 eV 188.30 nm f=0.0022 <S**2>=1.041
101A ->109A 0.13184
107A ->109A 0.11613
107A ->110A 0.13733
84B ->108B 0.14894
85B ->108B 0.11035
88B ->108B 0.73420
89B ->108B 0.14926
92B ->108B 0.10184
94B ->108B -0.10560
100B ->109B 0.32059
106B ->109B -0.20110
106B ->110B 0.28566

Excited State 29: 2.049-A 6.6238 eV 187.18 nm f=0.0044 <S**2>=0.799
87B ->108B 0.81722
88B ->108B 0.18603
89B ->108B -0.30498
90B ->108B -0.21466
92B ->108B -0.27711
106B ->109B 0.11371

Excited State 30: 2.147-A 6.7074 eV 184.85 nm f=0.0044 <S**2>=0.902
75B ->108B 0.11577
84B ->108B -0.27136
85B ->108B -0.23418
86B ->108B 0.61131
88B ->108B -0.18586
89B ->108B -0.17383

91B ->108B -0.12183
92B ->108B 0.40670
95B ->108B 0.12554
96B ->108B -0.16017
97B ->108B -0.12184
100B ->109B 0.17723
106B ->109B -0.21458
106B ->110B 0.12368

[Cu(II)LDHA]$^{2+}$

Excitation energies and oscillator strengths:

Excited State 1: 2.002-A 0.5597 eV 2215.33 nm f=0.0015 <S**2>=0.752
92B ->108B -0.26035
98B ->108B -0.13993
99B ->108B -0.11025
106B ->108B 0.92616
This state for optimization and/or second-order correction.
Total Energy, E(TD-HF/TD-DFT) = -2842.53931966
Copying the excited state density for this state as the 1-particle RhoCI density.

Excited State 2: 3.368-A 1.1111 eV 1115.84 nm f=0.0034 <S**2>=2.586
103A ->108A -0.19299
107A ->108A -0.44243
103B ->107B 0.24310
106B ->107B 0.82809

Excited State 3: 2.287-A 1.3852 eV 895.07 nm f=0.0230 <S**2>=1.057
103A ->108A 0.33220
106A ->108A 0.12891
107A ->108A 0.75751
106B ->107B 0.52889

Excited State 4: 3.352-A 1.5672 eV 791.14 nm f=0.0008 <S**2>=2.558
103A ->108A -0.45015
106A ->108A -0.21490
107A ->108A 0.43126
102B ->107B -0.14321
103B ->107B 0.62441
105B ->107B 0.32559
106B ->107B -0.12791

Excited State 5: 2.037-A 1.8327 eV 676.51 nm f=0.0027 <S**2>=0.787
103A ->108A 0.37732
105A ->108A 0.27851
106A ->108A 0.60425

```
   107A ->108A       -0.15326
   103B ->107B        0.31578
   105B ->107B        0.51579

 Excited State   6:  2.279-A      1.8709 eV  662.69 nm  f=0.0002  <S**2>=1.048
   104A ->108A        0.15345
   105A ->108A        0.89277
   106A ->108A       -0.37833

 Excited State   7:  3.397-A      1.9342 eV  641.00 nm  f=0.0000  <S**2>=2.636
   103A ->108A        0.28852
   105A ->108A       -0.23089
   106A ->108A       -0.52227
   103B ->107B       -0.29381
   105B ->107B        0.69557

 Excited State   8:  2.007-A      2.0041 eV  618.66 nm  f=0.0019  <S**2>=0.757
   105A ->108A       -0.10051
    94B ->108B        0.75836
    96B ->108B        0.49414
    97B ->108B        0.18807
    98B ->108B        0.10786
   100B ->108B        0.12272
   103B ->108B       -0.19454

 Excited State   9:  2.018-A      2.0359 eV  608.99 nm  f=0.0029  <S**2>=0.768
   103A ->108A        0.59737
   106A ->108A       -0.36400
   107A ->108A       -0.10711
    93B ->108B        0.20261
    95B ->108B        0.10137
   103B ->107B        0.51912
   105B ->107B       -0.34596

 Excited State  10:  2.009-A      2.0572 eV  602.68 nm  f=0.0006  <S**2>=0.760
   103A ->108A       -0.17419
   105A ->108A        0.12579
    86B ->108B        0.17668
    91B ->108B        0.32020
    93B ->108B        0.70026
    94B ->108B        0.23518
    95B ->108B        0.33388
    98B ->108B       -0.14518
   101B ->108B        0.11816
   103B ->107B       -0.13727
   103B ->108B        0.20283
```

```
Excited State  11:  3.436-A      2.1330 eV  581.26 nm  f=0.0000  <S**2>=2.701
   104A ->108A        0.78852
   104B ->107B       -0.59710

Excited State  12:  2.043-A      2.1467 eV  577.54 nm  f=0.0012  <S**2>=0.794
   104A ->108A        0.59029
   104B ->107B        0.79456

Excited State  13:  2.004-A      2.1817 eV  568.29 nm  f=0.0000  <S**2>=0.754
    86B ->108B        0.76948
    87B ->108B       -0.13192
    88B ->108B        0.13500
    90B ->108B       -0.19393
    91B ->108B       -0.18173
    92B ->108B        0.32939
    93B ->108B       -0.21956
    94B ->108B        0.10933
    98B ->108B       -0.12560
    99B ->108B       -0.14086
   103B ->108B        0.18404

Excited State  14:  2.007-A      2.6183 eV  473.54 nm  f=0.0031  <S**2>=0.757
    86B ->108B       -0.13959
   103B ->108B        0.16329
   105B ->108B        0.96461

Excited State  15:  2.007-A      2.7718 eV  447.30 nm  f=0.0008  <S**2>=0.757
   103B ->108B       -0.10079
   104B ->108B        0.98999

Excited State  16:  2.008-A      2.8090 eV  441.38 nm  f=0.0326  <S**2>=0.758
    86B ->108B       -0.13602
    92B ->108B        0.18520
    96B ->108B        0.10894
    98B ->108B        0.16372
    99B ->108B        0.13467
   102B ->108B        0.57320
   103B ->108B        0.66390
   104B ->108B        0.13334
   105B ->108B       -0.20518
   106B ->108B        0.17683

Excited State  17:  3.462-A      2.8309 eV  437.96 nm  f=0.0000  <S**2>=2.746
    99A ->108A        0.17485
   100A ->108A       -0.16211
```

101A ->108A -0.58157
102A ->108A -0.29392
98B ->107B -0.17293
99B ->107B 0.13307
100B ->107B -0.17059
101B ->107B 0.61321
102B ->107B 0.13629

Excited State 18: 2.987-A 2.9635 eV 418.37 nm f=0.0019 <S**2>=1.980
101A ->108A 0.13363
99B ->107B -0.10329
102B ->107B 0.93128
103B ->107B 0.17290

Excited State 19: 2.008-A 2.9890 eV 414.81 nm f=0.0058 <S**2>=0.758
86B ->108B -0.11706
88B ->108B -0.12730
90B ->108B 0.12839
92B ->108B -0.41060
93B ->108B -0.25589
94B ->108B 0.16716
97B ->108B 0.10047
98B ->108B -0.21218
99B ->108B -0.18401
102B ->108B -0.40959
103B ->108B 0.57687
105B ->108B -0.10341
106B ->108B -0.25992

Excited State 20: 3.112-A 3.1553 eV 392.93 nm f=0.0006 <S**2>=2.170
96A ->108A 0.24893
99A ->108A 0.14164
100A ->108A 0.15214
101A ->108A 0.15721
102A ->108A -0.21934
103A ->109A -0.10144
92B ->107B 0.11799
94B ->107B 0.11729
95B ->107B 0.15243
98B ->107B -0.15593
99B ->107B -0.18869
100B ->107B 0.71527
101B ->107B 0.26521
102B ->107B -0.20884
103B ->109B 0.10297

```
 Excited State  21:  2.546-A      3.2481 eV  381.71 nm  f=0.0062  <S**2>=1.371
     96A ->108A       -0.25196
     99A ->108A       -0.15075
    100A ->108A       -0.10580
    102A ->108A        0.73316
    100B ->107B        0.29140
    101B ->107B        0.46509

 Excited State  22:  2.193-A      3.3148 eV  374.04 nm  f=0.0133  <S**2>=0.952
    101A ->108A        0.68528
     98B ->107B       -0.10411
    100B ->107B       -0.48485
    101B ->107B        0.48305

 Excited State  23:  2.067-A      3.3862 eV  366.14 nm  f=0.0425  <S**2>=0.819
    102A ->108A        0.10016
     86B ->108B        0.18206
     88B ->108B       -0.10361
     92B ->108B       -0.45214
     93B ->108B       -0.13204
     94B ->108B        0.11081
     98B ->108B       -0.23891
     99B ->108B       -0.19161
    102B ->108B        0.66541
    103B ->108B       -0.22591
    106B ->108B       -0.19750

 Excited State  24:  3.184-A      3.4087 eV  363.73 nm  f=0.0021  <S**2>=2.285
     96A ->108A        0.32802
     98A ->108A       -0.10124
     99A ->108A        0.20270
    100A ->108A        0.18372
    101A ->108A       -0.26159
    102A ->108A        0.51195
     92B ->107B        0.12170
     92B ->108B        0.10054
     95B ->107B        0.23708
     96B ->107B       -0.14649
     98B ->107B       -0.29338
     99B ->107B       -0.30097
    100B ->107B       -0.26857
    101B ->107B       -0.10931
    102B ->108B       -0.12894

 Excited State  25:  2.104-A      3.6182 eV  342.66 nm  f=0.0372  <S**2>=0.857
     96A ->108A        0.39287
```

98A ->108A -0.14669
99A ->108A 0.28014
100A ->108A 0.26564
93B ->107B 0.15465
95B ->107B -0.21881
96B ->107B 0.14679
98B ->107B 0.36288
99B ->107B 0.36051
100B ->108B 0.45978
101B ->108B 0.17389

Excited State 26: 2.188-A 3.6970 eV 335.37 nm f=0.0757 <S**2>=0.947
96A ->108A -0.24779
99A ->108A -0.13078
100A ->108A -0.13705
102A ->108A -0.11537
92B ->107B 0.12285
93B ->107B 0.16375
94B ->107B -0.21866
94B ->108B -0.10012
95B ->107B 0.20383
96B ->107B -0.27868
97B ->107B -0.18872
98B ->107B -0.18952
99B ->107B -0.11043
100B ->108B 0.65566
101B ->108B 0.26455

Excited State 27: 3.324-A 3.7769 eV 328.27 nm f=0.0027 <S**2>=2.512
80A ->108A 0.10997
81A ->108A 0.10746
93A ->108A 0.10903
94A ->108A 0.12720
97A ->108A -0.11061
99A ->108A 0.40128
100A ->108A -0.25605
101A ->108A 0.18254
102A ->108A 0.11492
79B ->107B -0.12979
93B ->107B -0.14631
94B ->107B -0.38635
95B ->107B -0.25139
96B ->107B -0.16253
97B ->107B -0.24010
98B ->107B -0.26245
99B ->107B 0.27468

```
  100B ->107B        0.12751
  100B ->108B       -0.14382
  101B ->107B       -0.13394

Excited State  28:  3.176-A      3.8655 eV  320.75 nm  f=0.0017  <S**2>=2.271
   94A ->108A       -0.14296
   96A ->108A       -0.11398
   98A ->108A        0.10095
   99A ->108A        0.26110
  100A ->108A       -0.29830
  101A ->108A        0.14016
   92B ->107B        0.15497
   94B ->107B        0.56255
   96B ->107B        0.42863
   98B ->107B       -0.30601
   99B ->107B        0.17621
  100B ->108B        0.14142
  101B ->107B       -0.20240

Excited State  29:  2.798-A      3.9954 eV  310.32 nm  f=0.0001  <S**2>=1.707
   77A ->108A       -0.18942
   87A ->108A        0.37395
   88A ->108A       -0.22838
   94A ->108A        0.60675
   95A ->108A        0.12545
   96A ->108A        0.21166
  100A ->108A       -0.12078
   82B ->107B        0.15769
   85B ->107B        0.12731
   94B ->107B        0.19486
   96B ->107B        0.19401
   97B ->107B        0.15967
  101B ->108B        0.18285

Excited State  30:  3.112-A      4.0116 eV  309.06 nm  f=0.0007  <S**2>=2.171
   90A ->108A       -0.14197
   96A ->108A        0.17821
   97A ->108A        0.13295
  100A ->108A       -0.15487
   91B ->107B        0.24084
   92B ->107B       -0.21052
   93B ->107B        0.54017
   95B ->107B        0.39105
   96B ->107B       -0.16902
   97B ->107B       -0.17671
   99B ->107B        0.26572
```

101B ->108B   -0.32513

[Cu(I)LNO$_2$], conformer 1

Excitation energies and oscillator strengths:

```
 Excited State   1:      Singlet-A      3.1993 eV  387.54 nm  f=0.0016  <S**2>=0.000
      69 -> 75         0.23265
      71 -> 75        -0.12148
      72 -> 75        -0.32726
      73 -> 75         0.56224
 This state for optimization and/or second-order correction.
 Total Energy, E(TD-HF/TD-DFT) =  -2364.69119569
 Copying the excited state density for this state as the 1-particle RhoCI density.

 Excited State   2:      Singlet-A      3.5133 eV  352.90 nm  f=0.0008  <S**2>=0.000
      69 -> 75        -0.15758
      72 -> 75         0.54258
      73 -> 75         0.39703
      74 -> 75        -0.11137

 Excited State   3:      Singlet-A      3.5512 eV  349.13 nm  f=0.0525  <S**2>=0.000
      74 -> 75         0.68299

 Excited State   4:      Singlet-A      3.7381 eV  331.68 nm  f=0.0007  <S**2>=0.000
      69 -> 75         0.49371
      70 -> 75         0.20120
      71 -> 75        -0.32235
      72 -> 75         0.29009
      73 -> 75        -0.12746

 Excited State   5:      Singlet-A      4.1250 eV  300.56 nm  f=0.0003  <S**2>=0.000
      74 -> 76         0.68731

 Excited State   6:      Singlet-A      4.1574 eV  298.23 nm  f=0.0011  <S**2>=0.000
      70 -> 75        -0.35094
      71 -> 75        -0.22446
      73 -> 76         0.55220

 Excited State   7:      Singlet-A      4.1959 eV  295.49 nm  f=0.0049  <S**2>=0.000
      70 -> 75         0.48516
      71 -> 75         0.28640
      72 -> 76         0.13022
      73 -> 76         0.39179

 Excited State   8:      Singlet-A      4.2265 eV  293.35 nm  f=0.0011  <S**2>=0.000
      69 -> 75         0.41428
```

70 -> 75 -0.29839
71 -> 75 0.48449

Excited State 9: Singlet-A 4.6550 eV 266.35 nm f=0.0010 <S**2>=0.000
72 -> 76 0.66127
73 -> 76 -0.11732

Excited State 10: Singlet-A 4.9139 eV 252.32 nm f=0.0002 <S**2>=0.000
70 -> 76 0.55629
71 -> 76 0.39848

Excited State 11: Singlet-A 4.9618 eV 249.88 nm f=0.0001 <S**2>=0.000
69 -> 76 0.19134
70 -> 76 -0.38290
71 -> 76 0.52389

Excited State 12: Singlet-A 5.1881 eV 238.98 nm f=0.0001 <S**2>=0.000
73 -> 77 0.14613
74 -> 77 0.61544
74 -> 78 0.28030
74 -> 79 -0.10639

Excited State 13: Singlet-A 5.2119 eV 237.89 nm f=0.0005 <S**2>=0.000
73 -> 77 -0.24785
73 -> 78 -0.17086
74 -> 77 -0.22152
74 -> 78 0.58400

Excited State 14: Singlet-A 5.2249 eV 237.30 nm f=0.0012 <S**2>=0.000
73 -> 77 -0.45913
73 -> 78 0.50874
74 -> 77 0.13134

Excited State 15: Singlet-A 5.2649 eV 235.49 nm f=0.0001 <S**2>=0.000
73 -> 77 0.41204
73 -> 78 0.43455
73 -> 79 -0.14042
74 -> 77 -0.19086
74 -> 78 0.24634

Excited State 16: Singlet-A 5.3532 eV 231.61 nm f=0.0051 <S**2>=0.000
74 -> 79 0.68417

Excited State 17: Singlet-A 5.3783 eV 230.53 nm f=0.0051 <S**2>=0.000
73 -> 77 0.14745
73 -> 79 0.67092

```
Excited State  18:      Singlet-A      5.4531 eV  227.37 nm  f=0.0002  <S**2>=0.000
     64 -> 75        -0.44398
     67 -> 75         0.51393
     72 -> 78         0.12293

Excited State  19:      Singlet-A      5.5571 eV  223.11 nm  f=0.0056  <S**2>=0.000
     67 -> 75         0.11909
     72 -> 77         0.47896
     72 -> 78        -0.47871

Excited State  20:      Singlet-A      5.5610 eV  222.95 nm  f=0.0079  <S**2>=0.000
     72 -> 77         0.48779
     72 -> 78         0.48374
     72 -> 79        -0.10527

Excited State  21:      Singlet-A      5.6605 eV  219.04 nm  f=0.0113  <S**2>=0.000
     69 -> 76         0.65285
     70 -> 76         0.10947
     71 -> 76        -0.17182

Excited State  22:      Singlet-A      5.7472 eV  215.73 nm  f=0.0019  <S**2>=0.000
     72 -> 79         0.68179

Excited State  23:      Singlet-A      5.7685 eV  214.93 nm  f=0.0032  <S**2>=0.000
     74 -> 80         0.67167
     74 -> 83        -0.12355

Excited State  24:      Singlet-A      5.7949 eV  213.95 nm  f=0.0035  <S**2>=0.000
     73 -> 80         0.66712
     73 -> 83        -0.11385

Excited State  25:      Singlet-A      6.0370 eV  205.37 nm  f=0.0002  <S**2>=0.000
     70 -> 77         0.49881
     71 -> 77         0.27154
     71 -> 78         0.37435

Excited State  26:      Singlet-A      6.0581 eV  204.66 nm  f=0.0046  <S**2>=0.000
     69 -> 77         0.12047
     70 -> 77        -0.18640
     70 -> 78        -0.29000
     71 -> 77         0.56644
     71 -> 78        -0.13420

Excited State  27:      Singlet-A      6.0663 eV  204.38 nm  f=0.0054  <S**2>=0.000
     70 -> 77        -0.41493
```

```
     70 -> 78         0.33738
     71 -> 77         0.11453
     71 -> 78         0.41998

 Excited State  28:      Singlet-A      6.0881 eV  203.65 nm  f=0.0131  <S**2>=0.000
     69 -> 78        -0.13622
     70 -> 77         0.10932
     70 -> 78         0.51986
     71 -> 77         0.22619
     71 -> 78        -0.36015

 Excited State  29:      Singlet-A      6.2033 eV  199.87 nm  f=0.0151  <S**2>=0.000
     68 -> 75        -0.29101
     72 -> 80         0.61017

 Excited State  30:      Singlet-A      6.2435 eV  198.58 nm  f=0.0009  <S**2>=0.000
     70 -> 79         0.47286
     71 -> 79         0.47647
```

[Cu(I)LNO$_2$], conformer 2

```
 Excitation energies and oscillator strengths:

 Excited State   1:      Singlet-A      3.1646 eV  391.78 nm  f=0.0062  <S**2>=0.000
     72 -> 75         0.27007
     74 -> 75         0.64354
 This state for optimization and/or second-order correction.
 Total Energy, E(TD-HF/TD-DFT) =  -2364.69537862
 Copying the excited state density for this state as the 1-particle RhoCI density.

 Excited State   2:      Singlet-A      3.6469 eV  339.97 nm  f=0.0020  <S**2>=0.000
     72 -> 75         0.20475
     73 -> 75         0.65662

 Excited State   3:      Singlet-A      4.1290 eV  300.27 nm  f=0.0492  <S**2>=0.000
     68 -> 75        -0.11005
     70 -> 75        -0.13610
     71 -> 75         0.29469
     72 -> 75         0.51216
     73 -> 75        -0.23466
     74 -> 75        -0.20847

 Excited State   4:      Singlet-A      4.3320 eV  286.20 nm  f=0.0241  <S**2>=0.000
     68 -> 75        -0.14654
     69 -> 75        -0.33007
     70 -> 75         0.11742
     71 -> 75         0.49375
```

```
     72 -> 75        -0.27476
     74 -> 75         0.14364

 Excited State   5:      Singlet-A      4.5279 eV  273.82 nm  f=0.0138  <S**2>=0.000
     69 -> 75         0.18203
     70 -> 75         0.65373
     72 -> 75         0.10648
     74 -> 76         0.11431

 Excited State   6:      Singlet-A      4.6104 eV  268.92 nm  f=0.0062  <S**2>=0.000
     69 -> 75        -0.32641
     71 -> 75        -0.17468
     72 -> 76        -0.10259
     74 -> 76         0.57895

 Excited State   7:      Singlet-A      4.7044 eV  263.55 nm  f=0.0113  <S**2>=0.000
     68 -> 75         0.29716
     69 -> 75         0.37402
     70 -> 75        -0.15102
     71 -> 75         0.33445
     73 -> 76        -0.16723
     74 -> 76         0.27438

 Excited State   8:      Singlet-A      4.7773 eV  259.53 nm  f=0.0033  <S**2>=0.000
     68 -> 75         0.55013
     69 -> 75        -0.23197
     72 -> 76         0.13828
     73 -> 76         0.29971

 Excited State   9:      Singlet-A      4.7870 eV  259.00 nm  f=0.0038  <S**2>=0.000
     68 -> 75        -0.19519
     69 -> 75         0.15659
     72 -> 76        -0.14395
     73 -> 76         0.59322
     74 -> 76         0.15636

 Excited State  10:      Singlet-A      5.1018 eV  243.02 nm  f=0.0025  <S**2>=0.000
     68 -> 75        -0.13319
     72 -> 76         0.64693
     74 -> 76         0.17027

 Excited State  11:      Singlet-A      5.4568 eV  227.21 nm  f=0.0084  <S**2>=0.000
     74 -> 77         0.55450
     74 -> 78        -0.41375

 Excited State  12:      Singlet-A      5.5009 eV  225.39 nm  f=0.0041  <S**2>=0.000
```

```
      74 -> 77         0.39693
      74 -> 78         0.54020
      74 -> 79        -0.18908

 Excited State  13:      Singlet-A      5.6169 eV  220.74 nm  f=0.0034  <S**2>=0.000
      70 -> 76         0.10762
      74 -> 77         0.12799
      74 -> 78         0.12952
      74 -> 79         0.66151

 Excited State  14:      Singlet-A      5.6307 eV  220.19 nm  f=0.0035  <S**2>=0.000
      71 -> 76         0.66070

 Excited State  15:      Singlet-A      5.6384 eV  219.89 nm  f=0.0008  <S**2>=0.000
      70 -> 76         0.67034
      74 -> 79        -0.10077

 Excited State  16:      Singlet-A      5.7111 eV  217.09 nm  f=0.0007  <S**2>=0.000
      73 -> 77         0.68460

 Excited State  17:      Singlet-A      5.7312 eV  216.33 nm  f=0.0007  <S**2>=0.000
      73 -> 78         0.68958

 Excited State  18:      Singlet-A      5.8474 eV  212.03 nm  f=0.0032  <S**2>=0.000
      73 -> 79         0.69480

 Excited State  19:      Singlet-A      5.9972 eV  206.74 nm  f=0.0020  <S**2>=0.000
      72 -> 77         0.51152
      72 -> 78        -0.46517

 Excited State  20:      Singlet-A      6.0351 eV  205.44 nm  f=0.0069  <S**2>=0.000
      72 -> 77         0.45360
      72 -> 78         0.49311
      72 -> 79        -0.15057

 Excited State  21:      Singlet-A      6.1020 eV  203.19 nm  f=0.0027  <S**2>=0.000
      72 -> 80        -0.12364
      74 -> 80         0.66677

 Excited State  22:      Singlet-A      6.1805 eV  200.61 nm  f=0.0032  <S**2>=0.000
      72 -> 78         0.10503
      72 -> 79         0.67475

 Excited State  23:      Singlet-A      6.2919 eV  197.05 nm  f=0.0055  <S**2>=0.000
      66 -> 75         0.16062
      73 -> 80         0.63876
```

```
     73 -> 83        -0.13932

 Excited State  24:      Singlet-A      6.3176 eV  196.25 nm  f=0.0008  <S**2>=0.000
     65 -> 75        -0.34599
     66 -> 75         0.57739
     73 -> 80        -0.16296

 Excited State  25:      Singlet-A      6.3624 eV  194.87 nm  f=0.0024  <S**2>=0.000
     64 -> 75         0.12287
     65 -> 75        -0.31653
     66 -> 75        -0.19044
     68 -> 76        -0.19064
     69 -> 76         0.51068
     74 -> 83         0.10650

 Excited State  26:      Singlet-A      6.4076 eV  193.49 nm  f=0.0051  <S**2>=0.000
     65 -> 75         0.21946
     66 -> 75         0.12700
     67 -> 75         0.10550
     68 -> 76         0.47316
     69 -> 76         0.38560

 Excited State  27:      Singlet-A      6.5486 eV  189.33 nm  f=0.0039  <S**2>=0.000
     65 -> 75         0.27688
     66 -> 75         0.14761
     68 -> 76        -0.17719
     72 -> 80         0.36338
     74 -> 81        -0.10310
     74 -> 83         0.37780
     74 -> 86        -0.13954

 Excited State  28:      Singlet-A      6.5768 eV  188.52 nm  f=0.0062  <S**2>=0.000
     64 -> 75         0.18915
     65 -> 75         0.32550
     66 -> 75         0.20703
     68 -> 76        -0.30985
     69 -> 76         0.15515
     74 -> 80        -0.13164
     74 -> 81         0.13733
     74 -> 83        -0.29676

 Excited State  29:      Singlet-A      6.6337 eV  186.90 nm  f=0.0017  <S**2>=0.000
     70 -> 77        -0.39033
     70 -> 78         0.45541
     71 -> 77        -0.18788
     72 -> 80        -0.14142
```

74 -> 81 -0.22176

Excited State 30: Singlet-A 6.6423 eV 186.66 nm f=0.0069 <S**2>=0.000
70 -> 77 -0.26363
70 -> 78 0.20074
71 -> 77 0.10256
71 -> 78 -0.19819
72 -> 80 0.31283
74 -> 81 0.44188

[Cu(II)LNO$_2$]$^+$, conformer 1

Excitation energies and oscillator strengths:

Excited State 1: 2.006-A 0.8305 eV 1492.95 nm f=0.0043 <S**2>=0.756
63B -> 74B 0.10818
65B -> 74B 0.16504
66B -> 74B 0.29718
72B -> 74B 0.92075
This state for optimization and/or second-order correction.
Total Energy, E(TD-HF/TD-DFT) = -2364.61652613
Copying the excited state density for this state as the 1-particle RhoCI density.

Excited State 2: 2.023-A 1.4810 eV 837.18 nm f=0.0201 <S**2>=0.773
63B -> 74B 0.16677
70B -> 74B 0.12939
73B -> 74B 0.95382

Excited State 3: 2.008-A 1.8697 eV 663.13 nm f=0.0004 <S**2>=0.758
61B -> 74B 0.36960
62B -> 74B 0.46409
64B -> 74B -0.12912
65B -> 74B 0.17443
70B -> 74B 0.63012
71B -> 74B -0.35827
73B -> 74B -0.19353

Excited State 4: 2.002-A 2.1153 eV 586.12 nm f=0.0010 <S**2>=0.752
63B -> 74B -0.10128
68B -> 74B 0.97638

Excited State 5: 2.121-A 2.2108 eV 560.81 nm f=0.0001 <S**2>=0.875
74A -> 75A 0.17024
61B -> 74B 0.11653
62B -> 74B -0.12560
63B -> 74B 0.70788
65B -> 74B -0.48406

```
      67B -> 74B         0.14654
      70B -> 74B        -0.10493
      71B -> 74B        -0.26504
      73B -> 74B        -0.17468
      73B -> 75B        -0.18008

 Excited State   6:  3.355-A      2.3028 eV  538.40 nm  f=0.0005  <S**2>=2.564
      73A -> 75A        -0.10914
      74A -> 75A         0.68655
      63B -> 74B        -0.17704
      65B -> 74B         0.11430
      73B -> 74B         0.11602
      73B -> 75B        -0.67660

 Excited State   7:  2.010-A      3.1980 eV  387.70 nm  f=0.1072  <S**2>=0.760
      63B -> 74B         0.20225
      65B -> 74B        -0.13695
      69B -> 74B        -0.26741
      70B -> 74B         0.39350
      71B -> 74B         0.82522

 Excited State   8:  2.018-A      3.4153 eV  363.02 nm  f=0.0306  <S**2>=0.768
      74A -> 75A        -0.10562
      63B -> 74B         0.15013
      64B -> 74B        -0.19426
      65B -> 74B         0.39017
      66B -> 74B         0.55234
      69B -> 74B        -0.51151
      70B -> 74B        -0.25821
      72B -> 74B        -0.29957
      73B -> 75B        -0.10276

 Excited State   9:  2.009-A      3.4322 eV  361.24 nm  f=0.0203  <S**2>=0.759
      74A -> 75A         0.52205
      63B -> 74B         0.17997
      65B -> 74B         0.15810
      66B -> 74B         0.34145
      69B -> 74B         0.44270
      72B -> 74B        -0.18080
      73B -> 75B         0.53012

 Excited State  10:  2.012-A      3.4440 eV  360.00 nm  f=0.0346  <S**2>=0.762
      74A -> 75A        -0.43672
      62B -> 74B        -0.14520
      63B -> 74B         0.16216
      66B -> 74B         0.29399
```

```
     69B -> 74B        0.59599
     70B -> 74B        0.20591
     71B -> 74B        0.11251
     72B -> 74B       -0.14732
     73B -> 75B       -0.45822

 Excited State  11:  2.021-A      3.7173 eV  333.54 nm  f=0.0084  <S**2>=0.771
     59B -> 74B        0.12134
     61B -> 74B       -0.42839
     62B -> 74B       -0.52755
     63B -> 74B       -0.10126
     66B -> 74B        0.15427
     69B -> 74B       -0.27176
     70B -> 74B        0.54631
     71B -> 74B       -0.26661

 Excited State  12:  3.414-A      4.0223 eV  308.24 nm  f=0.0004  <S**2>=2.663
     70A -> 75A        0.38303
     71A -> 75A       -0.50555
     72A -> 75A        0.14408
     73A -> 75A        0.24152
     62B -> 75B       -0.10550
     70B -> 75B        0.64929
     72B -> 75B       -0.23108
     70B <- 75B        0.10307

 Excited State  13:  2.017-A      4.4456 eV  278.89 nm  f=0.0025  <S**2>=0.767
     63B -> 74B       -0.11738
     67B -> 74B        0.95299
     69B -> 74B        0.13865

 Excited State  14:  2.740-A      4.5447 eV  272.81 nm  f=0.0007  <S**2>=1.627
     69A -> 75A       -0.20900
     70A -> 75A        0.29209
     71A -> 75A        0.37460
     72A -> 75A       -0.13991
     73A -> 75A        0.74606
     74A -> 75A        0.12644
     67B -> 75B       -0.12192
     71B -> 75B        0.29000

 Excited State  15:  2.800-A      4.8453 eV  255.88 nm  f=0.0052  <S**2>=1.710
     71A -> 75A       -0.12590
     70B -> 75B        0.16518
     72B -> 75B        0.96359
```

```
Excited State  16:  2.014-A      4.9060 eV  252.72 nm  f=0.0017  <S**2>=0.764
   62B -> 74B       -0.22273
   63B -> 74B        0.43754
   64B -> 74B       -0.11266
   65B -> 74B        0.62489
   66B -> 74B       -0.58468

Excited State  17:  2.014-A      4.9292 eV  251.53 nm  f=0.0022  <S**2>=0.764
   64B -> 74B        0.94374
   65B -> 74B        0.26955

Excited State  18:  2.886-A      5.1693 eV  239.85 nm  f=0.0044  <S**2>=1.833
   70A -> 75A       -0.10126
   71A -> 75A        0.12117
   72A -> 75A        0.91823
   73A -> 75A        0.22775
   70B -> 75B       -0.14522
   71B -> 75B       -0.19623

Excited State  19:  2.811-A      5.1919 eV  238.80 nm  f=0.0005  <S**2>=1.725
   72A -> 75A        0.30995
   73A -> 75A       -0.34413
   67B -> 75B       -0.29750
   70B -> 75B        0.11058
   71B -> 75B        0.80309

Excited State  20:  2.015-A      5.2282 eV  237.14 nm  f=0.0004  <S**2>=0.765
   61B -> 74B        0.77654
   62B -> 74B       -0.57176
   63B -> 74B       -0.22023

Excited State  21:  2.062-A      5.5180 eV  224.69 nm  f=0.0007  <S**2>=0.813
   74A -> 76A        0.16820
   60B -> 74B        0.96351

Excited State  22:  3.149-A      5.5528 eV  223.28 nm  f=0.0018  <S**2>=2.228
   74A -> 76A        0.85608
   60B -> 74B       -0.18275
   73B -> 76B       -0.42846

Excited State  23:  2.027-A      5.5655 eV  222.77 nm  f=0.0080  <S**2>=0.777
   58B -> 74B       -0.18950
   59B -> 74B        0.95012
   62B -> 74B        0.18946

Excited State  24:  2.019-A      5.6146 eV  220.82 nm  f=0.0120  <S**2>=0.769
```

58B -> 74B 0.96471
59B -> 74B 0.18926
60B -> 74B 0.11878

Excited State 25: 2.024-A 5.7340 eV 216.22 nm f=0.0007 <S**2>=0.774
57B -> 74B 0.98032
73B -> 76B 0.10694

Excited State 26: 2.339-A 5.7504 eV 215.61 nm f=0.0033 <S**2>=1.117
71A -> 75A -0.17074
73A -> 76A 0.25636
74A -> 76A 0.38079
57B -> 74B -0.11758
70B -> 75B -0.11829
73B -> 76B 0.82483

Excited State 27: 2.385-A 5.8073 eV 213.50 nm f=0.0026 <S**2>=1.172
69A -> 75A -0.30126
70A -> 75A 0.65782
71A -> 75A 0.39002
73A -> 75A -0.41775
73A -> 76A 0.14131
71B -> 75B -0.27359
73B -> 76B 0.13733

Excited State 28: 3.257-A 5.8897 eV 210.51 nm f=0.0010 <S**2>=2.402
72A -> 76A -0.34311
72B -> 76B 0.90286

Excited State 29: 2.212-A 6.0797 eV 203.93 nm f=0.0018 <S**2>=0.974
73A -> 76A 0.92715
74A -> 76A -0.18105
72B -> 76B 0.10317
73B -> 76B -0.21400

Excited State 30: 2.300-A 6.1670 eV 201.04 nm f=0.0006 <S**2>=1.073
72A -> 76A 0.91137
72B -> 76B 0.35251

[Cu(II)LNO$_2$]$^+$, conformer 2

Excitation energies and oscillator strengths:

Excited State 1: 2.003-A 1.0746 eV 1153.79 nm f=0.0015 <S**2>=0.753
63B -> 74B -0.10886
64B -> 74B -0.10847
65B -> 74B -0.22487

```
   66B -> 74B        0.15249
   68B -> 74B        0.13461
   70B -> 74B       -0.13571
   73B -> 74B        0.91857
This state for optimization and/or second-order correction.
Total Energy, E(TD-HF/TD-DFT) =  -2364.61051790
Copying the excited state density for this state as the 1-particle RhoCI density.

Excited State   2:  2.003-A      2.2054 eV  562.20 nm  f=0.0105  <S**2>=0.753
   62B -> 74B        0.24370
   63B -> 74B        0.21046
   64B -> 74B        0.12542
   65B -> 74B        0.17531
   66B -> 74B        0.16641
   67B -> 74B       -0.34274
   68B -> 74B        0.51570
   69B -> 74B        0.32554
   70B -> 74B       -0.21913
   71B -> 74B       -0.25044
   72B -> 74B       -0.46034

Excited State   3:  2.003-A      2.2835 eV  542.95 nm  f=0.0131  <S**2>=0.753
   64B -> 74B        0.12381
   65B -> 74B        0.22808
   66B -> 74B        0.38096
   67B -> 74B        0.58627
   69B -> 74B        0.44489
   70B -> 74B       -0.23669
   71B -> 74B       -0.24421
   72B -> 74B        0.32959

Excited State   4:  2.004-A      2.4194 eV  512.46 nm  f=0.0023  <S**2>=0.754
   62B -> 74B        0.66791
   63B -> 74B        0.19674
   66B -> 74B       -0.41409
   67B -> 74B        0.33659
   69B -> 74B       -0.36907
   71B -> 74B       -0.22964

Excited State   5:  2.826-A      2.6224 eV  472.78 nm  f=0.0003  <S**2>=1.747
   72A -> 75A       -0.39253
   73A -> 75A        0.17280
   74A -> 75A        0.80168
   72B -> 75B       -0.38474

Excited State   6:  2.006-A      2.8814 eV  430.29 nm  f=0.1054  <S**2>=0.756
```

```
      62B -> 74B        0.13142
      67B -> 74B       -0.43798
      68B -> 74B        0.32136
      72B -> 74B        0.79758

 Excited State   7:  2.013-A      3.4786 eV  356.42 nm  f=0.0106  <S**2>=0.763
      62B -> 74B       -0.12183
      63B -> 74B        0.14305
      64B -> 74B        0.14375
      65B -> 74B        0.30613
      66B -> 74B       -0.27736
      67B -> 74B       -0.17386
      68B -> 74B       -0.21262
      69B -> 74B        0.19003
      70B -> 74B        0.54054
      71B -> 74B       -0.49149
      73B -> 74B        0.31997

 Excited State   8:  2.694-A      3.5967 eV  344.72 nm  f=0.0032  <S**2>=1.565
      69A -> 75A        0.22795
      73A -> 75A        0.23991
      74A -> 75A        0.34182
      69B -> 74B        0.12072
      69B -> 75B        0.19963
      70B -> 75B        0.16836
      71B -> 74B        0.15923
      71B -> 75B        0.14339
      72B -> 75B        0.68966
      73B -> 75B       -0.36487

 Excited State   9:  2.408-A      3.6327 eV  341.30 nm  f=0.0345  <S**2>=1.199
      69A -> 75A        0.23994
      70A -> 75A       -0.22923
      73A -> 75A        0.14102
      74A -> 75A       -0.19536
      62B -> 74B        0.24553
      63B -> 74B        0.19021
      64B -> 74B        0.16279
      65B -> 74B        0.19662
      69B -> 74B        0.24606
      69B -> 75B        0.17109
      70B -> 74B        0.24747
      71B -> 74B        0.57829
      72B -> 75B       -0.32771
      73B -> 74B        0.12355
```

```
Excited State  10:  3.085-A      3.7479 eV  330.81 nm  f=0.0139  <S**2>=2.129
   69A -> 75A         0.33255
   70A -> 75A        -0.28602
   73A -> 75A         0.22587
   74A -> 75A        -0.13563
   62B -> 74B        -0.12301
   63B -> 74B        -0.10196
   65B -> 75B        -0.13958
   66B -> 74B         0.12477
   66B -> 75B        -0.15438
   68B -> 75B        -0.10478
   69B -> 74B        -0.21420
   69B -> 75B         0.46168
   71B -> 74B        -0.40173
   72B -> 75B        -0.24811
   73B -> 75B        -0.33244

Excited State  11:  2.521-A      4.0085 eV  309.31 nm  f=0.0085  <S**2>=1.339
   70A -> 75A         0.24158
   72A -> 75A         0.15705
   73A -> 75A         0.45465
   74A -> 75A        -0.11076
   65B -> 74B        -0.15255
   66B -> 74B        -0.45476
   67B -> 74B        -0.11457
   68B -> 74B        -0.21779
   69B -> 74B         0.34479
   70B -> 74B        -0.41601
   72B -> 75B        -0.20845
   73B -> 75B        -0.15854

Excited State  12:  2.144-A      4.0648 eV  305.02 nm  f=0.0218  <S**2>=0.899
   69A -> 75A         0.13707
   73A -> 75A         0.49659
   62B -> 74B         0.13025
   65B -> 74B        -0.19400
   66B -> 74B         0.16200
   70B -> 74B         0.17040
   71B -> 74B        -0.15822
   72B -> 75B         0.19773
   73B -> 75B         0.71542

Excited State  13:  2.556-A      4.0971 eV  302.61 nm  f=0.0217  <S**2>=1.384
   70A -> 75A         0.20607
   73A -> 75A         0.19172
   62B -> 74B         0.17350
```

63B -> 74B -0.12142
64B -> 74B -0.13553
65B -> 74B -0.33523
65B -> 75B 0.10648
66B -> 74B 0.22243
67B -> 74B 0.15604
68B -> 74B 0.22859
69B -> 74B 0.14195
69B -> 75B -0.23982
70B -> 74B 0.53855
72B -> 75B -0.17230
73B -> 74B -0.11063
73B -> 75B -0.37054

Excited State 14: 2.612-A 4.1421 eV 299.32 nm f=0.0035 <S**2>=1.456
69A -> 75A -0.18958
70A -> 75A 0.11971
73A -> 75A 0.53327
74A -> 75A -0.13550
62B -> 74B -0.11715
63B -> 74B 0.15321
64B -> 74B 0.17311
65B -> 74B 0.44994
66B -> 74B 0.24217
69B -> 74B -0.45772
69B -> 75B -0.20634
73B -> 75B -0.13861

Excited State 15: 2.753-A 4.4087 eV 281.23 nm f=0.0013 <S**2>=1.645
69A -> 75A 0.32297
70A -> 75A 0.27326
71A -> 75A 0.13604
72A -> 75A 0.65876
73A -> 75A -0.12709
74A -> 75A 0.26982
62B -> 74B -0.10422
65B -> 74B 0.18252
68B -> 74B 0.11333
69B -> 74B -0.18385
70B -> 75B 0.12594
71B -> 75B 0.23084
72B -> 75B -0.20209

Excited State 16: 2.522-A 4.7749 eV 259.66 nm f=0.0059 <S**2>=1.340
69A -> 75A -0.12092
72A -> 75A 0.23619

73A -> 75A 0.15599
62B -> 74B -0.30950
62B -> 75B -0.11905
66B -> 74B -0.31891
66B -> 75B -0.13168
67B -> 74B 0.26241
68B -> 74B 0.47434
68B -> 75B 0.16145
70B -> 75B -0.24231
71B -> 75B -0.46230
72B -> 75B 0.11520

Excited State 17: 2.523-A 4.8179 eV 257.34 nm f=0.0075 <S**2>=1.341
72A -> 75A -0.38092
74A -> 75A -0.19423
62B -> 74B -0.25283
66B -> 74B -0.24882
66B -> 75B 0.11867
67B -> 74B 0.22385
67B -> 75B -0.13721
68B -> 74B 0.39777
68B -> 75B -0.11593
69B -> 75B 0.16380
70B -> 75B 0.29162
71B -> 75B 0.49156
73B -> 75B 0.16936

Excited State 18: 2.009-A 5.2825 eV 234.71 nm f=0.0009 <S**2>=0.759
62B -> 74B -0.28733
63B -> 74B 0.49443
64B -> 74B 0.60860
65B -> 74B -0.53005

Excited State 19: 2.036-A 5.3019 eV 233.85 nm f=0.0009 <S**2>=0.786
69A -> 75A -0.14160
70A -> 75A -0.14256
71A -> 75A -0.12058
72A -> 75A 0.10656
62B -> 74B -0.11647
63B -> 74B 0.69832
64B -> 74B -0.62877

Excited State 20: 2.316-A 5.3149 eV 233.28 nm f=0.0005 <S**2>=1.091
69A -> 75A 0.45106
70A -> 75A 0.48436
71A -> 75A 0.41292

```
   72A -> 75A       -0.35937
   73A -> 75A       -0.10269
   74A -> 75A       -0.15713
   63B -> 74B        0.16819
   64B -> 74B       -0.23509
   70B -> 75B       -0.12603
   71B -> 75B       -0.28156

Excited State  21:  2.007-A      5.6015 eV  221.34 nm  f=0.0006  <S**2>=0.757
   61B -> 74B        0.98266
   62B -> 74B        0.10386

Excited State  22:  2.008-A      5.9191 eV  209.46 nm  f=0.0028  <S**2>=0.758
   59B -> 74B        0.33833
   60B -> 74B        0.92677

Excited State  23:  2.017-A      5.9499 eV  208.38 nm  f=0.0054  <S**2>=0.767
   58B -> 74B       -0.15021
   59B -> 74B        0.91780
   60B -> 74B       -0.32662

Excited State  24:  3.179-A      5.9854 eV  207.15 nm  f=0.0001  <S**2>=2.276
   71A -> 75A       -0.25949
   58B -> 74B        0.10364
   68B -> 75B       -0.12532
   70B -> 75B        0.77634
   71B -> 75B       -0.49080
   73B -> 76B       -0.10342

Excited State  25:  2.031-A      5.9986 eV  206.69 nm  f=0.0048  <S**2>=0.781
   74A -> 76A        0.14588
   57B -> 74B        0.11459
   58B -> 74B        0.95172
   59B -> 74B        0.16204

Excited State  26:  2.409-A      6.0422 eV  205.20 nm  f=0.0003  <S**2>=1.201
   69A -> 75A       -0.22897
   70A -> 75A       -0.39112
   71A -> 75A        0.80136
   72A -> 75A        0.10615
   68B -> 75B       -0.13405
   70B -> 75B        0.25955
   71B -> 75B       -0.14367

Excited State  27:  2.099-A      6.0791 eV  203.95 nm  f=0.0002  <S**2>=0.851
   74A -> 76A       -0.47095
```

57B -> 74B 0.85199

Excited State 28: 2.335-A 6.0863 eV 203.71 nm f=0.0047 <S**2>=1.113
71A -> 75A -0.10020
74A -> 76A 0.76958
57B -> 74B 0.48276
58B -> 74B -0.18659
69B -> 75B -0.10500
73B -> 76B 0.21538

Excited State 29: 3.059-A 6.1695 eV 200.96 nm f=0.0035 <S**2>=2.089
68A -> 75A 0.15948
65B -> 75B 0.12845
66B -> 75B 0.23439
67B -> 75B -0.24318
68B -> 75B 0.73912
69B -> 75B 0.31684
70B -> 75B 0.14800
72B -> 75B -0.12233
73B -> 76B 0.31198

Excited State 30: 3.156-A 6.2277 eV 199.09 nm f=0.0016 <S**2>=2.240
71A -> 75A -0.12427
73A -> 76A -0.42450
74A -> 76A -0.21069
68B -> 75B -0.29892
73B -> 76B 0.76003

Geometries (at ωB97X-D/def2SVP level)

$[Cu(I)L]^+$

E = -2159.556594

C	1.378462	-1.099257	1.093671
N	1.713247	-0.057661	0.102478
Cu	0.015975	0.012599	-1.181040
N	-0.790125	1.508469	0.101443
C	0.264069	1.731374	1.110625
C	1.660545	1.310171	0.641202
C	0.316199	-2.090295	0.606556
N	-0.894573	-1.443062	0.078015
C	-1.628772	-0.648078	1.082148
C	-1.956279	0.774705	0.617115
C	2.977581	-0.330449	-0.577033
C	-1.175840	2.745609	-0.573508
C	-1.763583	-2.390448	-0.616175

H	1.046010	-0.621019	2.024452
H	2.278526	-1.672391	1.369478
H	0.736732	-2.702334	-0.205902
H	0.075471	-2.780773	1.438802
H	-1.047619	-0.613377	2.013057
H	-2.574923	-1.145219	1.351047
H	-2.695387	0.728783	-0.197201
H	-2.435302	1.314953	1.457333
H	0.001731	1.197818	2.033739
H	0.307017	2.795333	1.394856
H	1.992208	1.986795	-0.161047
H	2.366501	1.440274	1.485027
H	-2.620250	-1.856454	-1.049399
H	-1.212186	-2.872309	-1.435162
H	-2.146346	-3.176536	0.062323
H	-0.284581	3.227590	-0.997885
H	-1.867765	2.521918	-1.396996
H	-1.666298	3.458950	0.115965
H	3.129712	0.394782	-1.388118
H	3.840493	-0.274135	0.113621
H	2.949793	-1.335750	-1.018954

[Cu(I)L(H_2O)]$^+$

E = -2235.936986

C	-0.013207	0.018276	0.024023
N	-0.000509	0.002027	1.483152
C	1.363249	0.002719	2.022125
C	1.414228	0.280615	3.525820
N	0.662846	1.485071	3.912443
C	1.538662	2.630657	4.135906
C	-0.858818	-1.060418	2.030893
C	-2.209003	-0.541252	2.529006
N	-2.084311	0.596889	3.444238
C	-3.362735	1.259934	3.677032
Cu	-0.746972	1.891630	2.294213
O	-1.167869	3.632182	1.382051
C	-1.384932	0.283830	4.700111
C	-0.267713	1.277261	5.026605
H	-0.331182	-1.572254	2.846642
H	-1.045544	-1.840455	1.273939
H	-2.805645	-0.202406	1.667969
H	-2.763148	-1.382324	2.992179
H	-0.976978	-0.734452	4.647463
H	-2.091383	0.267715	5.546964
H	-0.712511	2.255853	5.265076
H	0.257162	0.932753	5.940313

H	1.027997	-0.585270	4.079980
H	2.471852	0.366657	3.826360
H	1.922716	0.787800	1.490240
H	1.884115	-0.954943	1.820546
H	-0.722380	4.399233	1.762800
H	-2.104593	3.865361	1.384187
H	-3.210009	2.150674	4.302325
H	-3.793180	1.588367	2.720486
H	-4.092878	0.598209	4.183041
H	2.172756	2.790314	3.252729
H	0.936684	3.537515	4.289058
H	2.195443	2.490557	5.016779
H	0.533052	0.897366	-0.345548
H	0.448462	-0.890983	-0.408592
H	-1.048921	0.086323	-0.336748

[Cu(I)LHasc]$^+$

E = -2843.211234

C	3.442881	-0.728644	-0.652674
C	2.272996	-0.155239	-1.381780
C	1.186118	-0.957768	-1.286596
C	1.598364	-2.071027	-0.401707
O	2.912074	-1.927108	-0.081260
O	2.334801	1.011355	-2.053330
O	0.011555	-0.787986	-1.826467
Cu	-1.365892	-0.151049	-0.632563
N	-3.237505	0.977768	-0.737762
C	-4.216173	-0.040255	-0.344680
C	-3.846619	-0.744534	0.962970
N	-2.471900	-1.251997	0.954982
C	-1.674966	-0.887910	2.126163
C	-1.482771	0.623466	2.280933
N	-0.958051	1.257631	1.067469
C	-1.703144	2.426728	0.607903
C	-3.142413	2.100754	0.208349
O	0.958982	-3.004496	0.027269
C	4.037985	0.120683	0.464724
O	5.108946	-0.590770	1.027876
C	-2.405885	-2.678215	0.661265
C	4.538504	1.477639	-0.020080
O	5.241743	2.034319	1.069622
C	0.473262	1.505508	1.138884
C	-3.444297	1.437203	-2.107190
H	-3.983403	-0.058421	1.809679
H	-4.569046	-1.561164	1.133990
H	-4.256655	-0.784002	-1.155618

H	-5.235100	0.386467	-0.248121
H	-3.734375	1.873867	1.105040
H	-3.600116	3.009697	-0.217607
H	-1.168662	2.828173	-0.267598
H	-1.722035	3.234884	1.368088
H	-2.434441	1.095827	2.560849
H	-0.811408	0.799932	3.139049
H	-0.690103	-1.365986	2.002924
H	-2.112386	-1.286401	3.065115
H	4.263368	-1.016111	-1.334442
H	3.242374	0.289930	1.218032
H	5.202542	1.331757	-0.891484
H	3.695389	2.112805	-0.334912
H	5.817405	2.734337	0.748863
H	5.637163	0.079745	1.484481
H	-1.360666	-2.973078	0.494170
H	-2.835795	-3.294068	1.477343
H	-2.968344	-2.889906	-0.259657
H	0.989736	0.582744	1.437469
H	0.854479	1.789624	0.147143
H	0.733550	2.302170	1.864813
H	-2.647523	2.140752	-2.386319
H	-3.399088	0.584410	-2.798818
H	-4.420988	1.944738	-2.236173
H	1.449310	1.151063	-2.423353

[Cu(I)LDHA]$^+$

E = -2842.433679

C	-1.255477	1.487085	-0.378745
C	-2.342253	2.500541	-0.124314
O	-3.516763	1.887855	-0.322966
C	-3.418575	0.500253	-0.704159
C	-1.933192	0.201183	-0.719411
O	-2.225677	3.645818	0.194410
C	-4.233845	-0.351258	0.263244
O	-5.551505	0.112237	0.218569
O	-1.371259	-0.841242	-0.946368
Cu	1.274978	0.310872	-0.246598
N	1.351287	-1.049906	1.385653
C	1.404777	-2.318333	0.638712
C	2.455697	-2.297829	-0.469793
N	2.350145	-1.100535	-1.335932
C	3.642147	-0.421923	-1.547910
C	4.218397	0.171107	-0.260721
N	3.239244	0.993757	0.467809
C	3.114526	0.665812	1.891600

C	2.590207	-0.750340	2.134303
O	-0.049357	1.679016	-0.345710
C	-4.199409	-1.834421	-0.102728
O	-5.158216	-2.438912	0.732255
C	1.691405	-1.413881	-2.608545
C	3.433814	2.422856	0.241160
C	0.167963	-0.968859	2.241866
H	4.582990	-0.630629	0.394960
H	5.111729	0.762115	-0.520537
H	3.467322	0.384623	-2.275890
H	4.383607	-1.109011	-1.997099
H	3.463325	-2.342863	-0.037377
H	2.352700	-3.215289	-1.069925
H	0.409512	-2.475590	0.198110
H	1.606159	-3.174557	1.309761
H	3.359268	-1.486889	1.868067
H	2.424572	-0.879469	3.215639
H	2.415048	1.393051	2.331749
H	4.077047	0.781192	2.426952
H	-3.832948	0.404288	-1.720892
H	-3.803248	-0.226566	1.277852
H	-4.462234	-1.951701	-1.169443
H	-3.192229	-2.254345	0.052186
H	-5.405821	-3.292473	0.364779
H	-6.083132	-0.619828	0.565211
H	2.599349	2.987586	0.679860
H	4.379993	2.789196	0.682483
H	3.451109	2.627019	-0.838400
H	0.112532	0.025546	2.705907
H	-0.739820	-1.122873	1.643426
H	0.188482	-1.730213	3.042937
H	0.716206	-1.877198	-2.411965
H	1.523803	-0.489853	-3.178320
H	2.300712	-2.103920	-3.220271

$[Cu(II)L]^{2+}$

E = -2159.359233

C	1.401557	-1.106547	1.104777
N	1.722945	-0.061646	0.100417
Cu	-0.037957	-0.002382	-1.076019
N	-0.810437	1.427108	0.109193
C	0.259319	1.701893	1.113459
C	1.662306	1.311103	0.644031
C	0.320941	-2.071710	0.616763
N	-0.888368	-1.373558	0.107748
C	-1.649187	-0.640290	1.161268

C	-2.001820	0.760612	0.692806
C	3.001856	-0.319929	-0.577813
C	-1.169913	2.634964	-0.656242
C	-1.757829	-2.268886	-0.680191
H	1.101486	-0.632825	2.048015
H	2.298103	-1.695834	1.344579
H	0.709377	-2.684482	-0.209264
H	0.036134	-2.760071	1.428870
H	-1.046532	-0.615227	2.076152
H	-2.564597	-1.193935	1.410763
H	-2.771483	0.721128	-0.090295
H	-2.407479	1.357646	1.525380
H	0.007501	1.176795	2.041399
H	0.255135	2.772809	1.359560
H	2.001009	1.991348	-0.151088
H	2.353827	1.438404	1.494765
H	-2.624763	-1.707205	-1.048904
H	-1.202330	-2.682399	-1.531458
H	-2.108028	-3.099637	-0.047658
H	-0.265447	3.080800	-1.089241
H	-1.867069	2.374568	-1.462637
H	-1.646702	3.370417	0.010547
H	3.147831	0.413462	-1.381164
H	3.845887	-0.253012	0.128724
H	2.985173	-1.322905	-1.022705

$[Cu(II)L(H_2O)]^{2+}$

E = -2235.756523

C	1.732132	0.750197	-1.237677
N	1.494238	-0.538368	-0.541347
Cu	0.016665	-0.143377	0.898535
O	0.160269	-1.735772	2.044272
C	1.299651	1.920033	-0.365822
N	-0.065561	1.696787	0.180611
C	-1.125310	1.757820	-0.869891
C	-2.139108	0.633687	-0.718775
N	-1.466017	-0.666092	-0.523820
C	-0.669327	-1.096993	-1.699506
C	0.756443	-1.531981	-1.347563
C	2.731093	-1.105344	0.014948
C	-2.403927	-1.710015	-0.090333
C	-0.362767	2.612584	1.300915
H	1.194615	0.751699	-2.193885
H	2.795933	0.859937	-1.492113
H	1.980892	2.026121	0.490175
H	1.331447	2.865788	-0.930335

H	-0.642404	1.716654	-1.852285
H	-1.634670	2.730209	-0.821572
H	-2.776313	0.809224	0.159865
H	-2.800229	0.616692	-1.602271
H	-0.649415	-0.289045	-2.440791
H	-1.166439	-1.941431	-2.198391
H	0.728964	-2.462197	-0.761194
H	1.297038	-1.753550	-2.283634
H	-0.624548	-2.100559	2.476854
H	0.877181	-1.783110	2.692841
H	-1.373024	2.413906	1.680843
H	0.365948	2.465842	2.108822
H	-0.308400	3.657900	0.958643
H	-1.856278	-2.643154	0.096165
H	-2.904077	-1.400703	0.836519
H	-3.169834	-1.898599	-0.860908
H	2.493168	-1.990904	0.617905
H	3.423482	-1.400073	-0.790464
H	3.222917	-0.362769	0.656006

$[Cu(II)L(H_2O)_2]^{2+}$

E = -2312.136702

C	-1.137224	0.297023	-1.996693
N	-0.323754	1.229386	-1.192983
Cu	-0.071007	0.231381	0.763005
O	-1.604339	0.885753	1.986958
C	-1.929351	-0.669010	-1.118785
N	-1.086440	-1.333821	-0.094306
C	-0.086152	-2.261377	-0.692368
C	1.284634	-2.059128	-0.075550
N	1.635651	-0.621168	-0.041477
C	1.812295	-0.040133	-1.402799
C	1.090661	1.290778	-1.587899
O	0.919879	1.758345	1.677442
C	-0.913221	2.568345	-1.120684
C	2.834965	-0.399560	0.783328
C	-1.923439	-2.040206	0.893794
H	-0.492115	-0.255666	-2.691845
H	-1.847086	0.845394	-2.634185
H	-2.722074	-0.128146	-0.583307
H	-2.427141	-1.427411	-1.745929
H	-0.053670	-2.100160	-1.775750
H	-0.406775	-3.302966	-0.551775
H	1.297254	-2.426234	0.960807
H	2.042784	-2.632955	-0.634789
H	1.469087	-0.767717	-2.147296

H	2.883135	0.106254	-1.602845
H	1.567164	2.060508	-0.963007
H	1.209087	1.607409	-2.640232
H	0.289953	2.269768	2.205584
H	1.683214	1.608292	2.251129
H	-1.748539	0.496695	2.860167
H	-2.433099	1.313924	1.736365
H	-1.286707	-2.491430	1.665681
H	-2.611778	-1.334273	1.370088
H	-2.506613	-2.833141	0.399889
H	3.102395	0.664519	0.769086
H	2.649350	-0.722227	1.816629
H	3.685733	-0.973924	0.383125
H	-0.370876	3.172455	-0.380527
H	-0.873237	3.083416	-2.096813
H	-1.964233	2.499943	-0.810300

$[Cu(II)L(H_2O)_3]^{2+}$

E = -2388.50488020
Cu -0.366972 0.070410 -0.553719
O -2.692359 0.232090 -0.950191
H -3.043606 0.943007 -1.500937
H -3.463835 -0.123008 -0.492404
N 1.879963 -0.022525 -0.304048
C 2.610340 -0.118930 -1.566717
N -0.358876 -1.505741 0.794601
C -1.460520 -2.445315 0.527162
N -0.311041 1.294222 1.111879
C -1.012669 2.563583 0.868341
O -0.212728 1.591532 -1.947775
O -0.669593 -1.050183 -2.270318
C 2.119738 -1.177167 0.575727
C 0.950422 -2.159597 0.564302
C 2.081330 1.280857 0.337226
C 1.109898 1.527926 1.487055
C -0.482978 -0.931406 2.161412
C -1.041610 0.478192 2.105979
H 2.322565 -0.832760 1.598317
H 3.026331 -1.722779 0.271069
H 0.892566 -2.661113 -0.412155
H 1.115862 -2.947246 1.319052
H 0.498571 -0.944344 2.648751
H -1.134316 -1.563590 2.781212
H -2.096137 0.456714 1.796456
H -1.001208 0.946005 3.104376
H 1.360510 0.891705 2.343688

H 1.231950 2.561999 1.840116
H 1.935631 2.047752 -0.437719
H 3.111565 1.397884 0.721817
H -0.285084 1.223081 -2.839406
H -0.687856 2.432022 -1.961066
H -1.622222 -1.158846 -2.408337
H -0.226310 -1.820672 -2.646302
H -0.428366 3.179908 0.173752
H -2.002430 2.361924 0.439853
H -1.134300 3.125454 1.808451
H -2.421337 -1.960392 0.737345
H -1.441167 -2.759309 -0.522100
H -1.370749 -3.336174 1.168994
H 2.279025 0.677205 -2.246747
H 3.701559 -0.027274 -1.417912
H 2.408383 -1.087094 -2.043988

[Cu(II)LAsc], conformer 1
E = -2842.60944767
C 1.327592 1.549608 0.248253
C 2.433026 2.496354 0.011908
O 3.609347 1.875401 0.280199
C 3.409352 0.517589 0.684499
C 1.898707 0.314865 0.687844
O 2.398822 3.646504 -0.353492
C 4.163912 -0.404545 -0.263143
O 5.524327 -0.061377 -0.223110
O 1.323543 -0.732219 1.006314
O 0.095929 1.837554 0.092875
Cu -1.245051 0.458215 0.141678
N -1.352100 -1.262271 -1.218828
C -1.311090 -2.343657 -0.230986
C -2.341154 -2.163773 0.885237
N -2.289127 -0.828845 1.508535
C -3.599519 -0.179343 1.617511
C -4.226519 0.126031 0.254594
N -3.308553 0.832659 -0.645767
C -3.193990 0.246611 -1.981345
C -2.610609 -1.168454 -1.969485
C 4.009240 -1.878181 0.100302
O 4.915738 -2.569561 -0.732468
C -1.575575 -0.853161 2.783117
C -3.560902 2.266311 -0.685916
C -0.182879 -1.262670 -2.089198
H -4.560444 -0.806522 -0.219937
H -5.147007 0.711907 0.419306

H -3.450710 0.762971 2.167099
H -4.310763 -0.788549 2.210510
H -3.352769 -2.343808 0.497242
H -2.176400 -2.949981 1.640899
H -0.302988 -2.333583 0.206880
H -1.466529 -3.336556 -0.699895
H -3.342024 -1.871003 -1.547342
H -2.468754 -1.492878 -3.014535
H -2.531171 0.902734 -2.567091
H -4.169399 0.223867 -2.509087
H 3.820008 0.403054 1.701819
H 3.750830 -0.249547 -1.282122
H 4.261678 -2.016692 1.167373
H 2.969948 -2.209129 -0.050348
H 5.076470 -3.443662 -0.365888
H 5.983036 -0.840363 -0.569740
H -2.762432 2.770983 -1.248189
H -4.533358 2.511245 -1.157729
H -3.561060 2.672416 0.335523
H -0.206472 -0.379604 -2.743616
H 0.728938 -1.210267 -1.479024
H -0.134154 -2.167776 -2.726683
H -0.566516 -1.255521 2.625244
H -1.471167 0.170687 3.169327
H -2.101072 -1.465396 3.542075

[Cu(II)LAsc], conformer 2
E = -2842.60131571
C -1.455652 0.545630 -0.423827
O -2.613280 1.132222 -0.740860
C -3.683713 0.173439 -0.746408
C -3.035573 -1.165249 -0.390503
C -1.626823 -0.893381 -0.186817
C -4.767544 0.639038 0.213256
C -5.964143 -0.306306 0.245956
O -6.942237 0.351625 1.022410
O -3.639306 -2.231958 -0.309153
O -0.699659 -1.681237 0.127030
Cu 1.338112 0.372161 -0.018585
O -0.426056 1.214717 -0.367286
N 3.236542 1.533843 0.043405
C 2.976556 2.921825 -0.313303
C 4.165477 0.883791 -0.889684
C 3.456818 0.006503 -1.925619
N 2.520001 -0.941646 -1.324262
C 1.623622 -1.549264 -2.299084

C 3.589119 1.376137 1.455003
C 3.549988 -0.080504 1.923844
N 2.291265 -0.756212 1.583773
C 1.379280 -0.845566 2.718549
C 2.460336 -2.050876 0.916621
C 3.148223 -1.937657 -0.445822
O -5.197268 1.912624 -0.191439
H 4.889243 0.281023 -0.324694
H 4.771577 1.633776 -1.426483
H 2.877629 0.649607 -2.606529
H 4.225361 -0.502656 -2.542782
H 4.210107 -1.688713 -0.313502
H 3.142254 -2.934635 -0.919152
H 1.452296 -2.464013 0.773858
H 3.033822 -2.762259 1.545579
H 4.392592 -0.635366 1.489084
H 3.723717 -0.098594 3.013300
H 2.861084 1.960820 2.038947
H 4.591023 1.795528 1.680712
H -4.092100 0.144846 -1.770124
H -4.325064 0.681144 1.230424
H -6.320301 -0.473146 -0.786933
H -5.678230 -1.281018 0.670448
H -7.799978 -0.047658 0.851684
H -6.062045 2.024560 0.229074
H 2.179053 3.329671 0.323883
H 3.875212 3.561548 -0.204339
H 2.636310 2.979534 -1.356916
H 1.223975 0.153679 3.149822
H 0.408632 -1.224835 2.371288
H 1.766202 -1.511803 3.515697
H 0.872760 -2.153954 -1.772749
H 1.088852 -0.763282 -2.852028
H 2.162715 -2.187657 -3.027600

[Cu(II)LAsc], conformer 3

E = -2842.59815556
C -2.343345 0.048532 -0.721149
O -1.195504 -0.735809 -0.360978
C -1.557515 -1.981652 0.115830
C -3.017558 -2.142818 -0.025936
C -3.529698 -0.901150 -0.567126
O -0.713817 -2.725874 0.550540
Cu 0.731279 -0.076632 -0.308772
N 2.059919 1.555337 -0.777263
C 2.100935 2.236279 0.523371
C 2.416731 1.296291 1.691277

N 1.553096 0.111340 1.720959
C 0.450617 0.240729 2.661906
O -3.647597 -3.180891 0.286524
O -4.688276 -0.594063 -0.845905
C -2.405590 1.265940 0.187893
C -3.560483 2.197530 -0.155818
O -3.368315 3.344855 0.642386
N 2.513918 -1.319479 -0.659621
C 2.145734 -2.616234 -1.215632
O -1.194860 1.972597 0.070618
C 2.254541 -1.167742 1.830654
C 3.182003 -1.447296 0.645018
C 3.236284 -0.485528 -1.621866
C 3.351599 0.973753 -1.178204
C 1.495424 2.411998 -1.817326
H 3.468279 0.981481 1.648367
H 2.326004 1.872534 2.628240
H 1.108607 2.680854 0.681255
H 2.838280 3.064243 0.523067
H 4.062723 1.054572 -0.345232
H 3.802085 1.554009 -2.000669
H 2.682678 -0.530938 -2.572582
H 4.254383 -0.875583 -1.825327
H 4.047936 -0.772037 0.679320
H 3.601602 -2.460286 0.768168
H 1.482983 -1.951415 1.869721
H 2.847969 -1.238568 2.765417
H -2.214561 0.365195 -1.768587
H -2.532125 0.905076 1.228530
H -3.515925 2.445263 -1.231692
H -4.523804 1.702677 0.042291
H -3.873264 4.075092 0.273223
H -1.384215 2.860727 0.409052
H -0.227459 -0.618351 2.556680
H 0.794263 0.290739 3.714455
H -0.121657 1.150752 2.431893
H 1.507914 -3.152375 -0.501088
H 1.564640 -2.473868 -2.138106
H 3.032722 -3.236926 -1.452708
H 1.457646 1.861396 -2.767796
H 0.469536 2.689096 -1.542023
H 2.095999 3.330272 -1.970829

[Cu(II)LHAsc]$^{+}$

E = -2843.055138

C -1.156377 1.048626 -0.984238
C -1.786950 2.157942 -0.233600

O	-3.119152	1.934412	-0.155501
C	-3.453303	0.673544	-0.733758
C	-2.147648	0.176172	-1.291667
O	-1.274368	3.133746	0.254662
C	-4.094075	-0.187402	0.355443
O	-5.295495	0.429591	0.722689
O	-1.979784	-0.966722	-1.963686
O	0.113360	1.039165	-1.288371
Cu	1.497846	0.017549	-0.653205
N	0.946665	-1.234289	1.006317
C	1.770273	-2.423395	0.726574
C	3.201657	-2.047240	0.368233
N	3.256405	-0.971618	-0.657891
C	4.236159	0.089141	-0.317926
C	3.793001	0.892471	0.901660
N	2.389330	1.330865	0.766201
C	1.534807	1.030488	1.926372
C	1.385600	-0.469369	2.195249
C	-4.375682	-1.624574	-0.074684
O	-5.136271	-2.185249	0.967724
C	3.509277	-1.527191	-1.999129
C	2.272841	2.733022	0.352659
C	-0.475535	-1.573521	1.072107
H	3.908670	0.301923	1.819654
H	4.461178	1.758757	1.020372
H	4.309258	0.753448	-1.191517
H	5.236416	-0.345265	-0.150742
H	3.743780	-1.713196	1.260609
H	3.735947	-2.938546	0.008410
H	1.299391	-2.954373	-0.114161
H	1.780883	-3.119918	1.584374
H	2.331078	-0.884411	2.567008
H	0.665052	-0.600245	3.016645
H	0.548490	1.470631	1.712300
H	1.917503	1.516219	2.842190
H	-4.200231	0.848461	-1.527224
H	-3.388231	-0.215048	1.210749
H	-4.944115	-1.621063	-1.023431
H	-3.433279	-2.177590	-0.229445
H	-5.589141	-2.969997	0.645269
H	-5.802166	-0.257777	1.178841
H	1.221680	2.958103	0.130144
H	2.639391	3.411289	1.143197
H	2.867067	2.897051	-0.556429
H	-1.058750	-0.663093	1.260463
H	-0.800577	-1.998913	0.113329

H	-0.681292	-2.296687	1.880455
H	2.750648	-2.286121	-2.231475
H	3.452806	-0.729412	-2.751607
H	4.507209	-1.992953	-2.047876
H	-2.813604	-1.306726	-2.309450

[Cu(II)LDHA]$^{2+}$, conformer 3

E = -2842.239742

C	-1.244612	1.769617	-0.176433
C	-2.524352	2.591307	-0.081645
O	-3.525914	1.755236	-0.378550
C	-3.162732	0.393210	-0.707422
C	-1.676446	0.372998	-0.558219
O	-2.640317	3.739886	0.199004
C	-3.941316	-0.587692	0.171819
O	-5.291980	-0.367813	-0.087900
O	-0.852706	-0.506052	-0.671291
Cu	1.126145	0.185556	-0.296764
N	1.422871	-0.658223	1.674971
C	1.491762	-2.080381	1.293416
C	2.386429	-2.327268	0.079642
N	2.081094	-1.438119	-1.077560
C	3.294150	-0.833264	-1.677106
C	3.943901	0.131394	-0.702487
N	2.930655	1.054712	-0.115230
C	3.053495	1.201673	1.357976
C	2.721319	-0.088626	2.103386
O	-0.087933	2.039983	0.009303
C	-3.576380	-2.040664	-0.137221
O	-4.520240	-2.803167	0.570626
C	1.271005	-2.133800	-2.093541
C	2.949398	2.365542	-0.795088
C	0.362437	-0.414907	2.659956
H	4.445584	-0.405545	0.110177
H	4.724674	0.711018	-1.213609
H	2.981607	-0.306823	-2.589951
H	4.009741	-1.616107	-1.977706
H	3.440985	-2.202863	0.350958
H	2.277800	-3.376212	-0.229709
H	0.464686	-2.410787	1.077596
H	1.860366	-2.706359	2.125012
H	3.515767	-0.832997	1.964309
H	2.711299	0.129784	3.181127
H	2.362168	2.001204	1.660941
H	4.070822	1.538044	1.616334
H	-3.426553	0.222427	-1.765421

H	-3.696758	-0.375579	1.231189
H	-3.650307	-2.212345	-1.225645
H	-2.543306	-2.264513	0.179895
H	-4.562882	-3.687377	0.194023
H	-5.738067	-1.178621	0.200437
H	2.142421	3.000089	-0.411244
H	3.914412	2.864776	-0.616413
H	2.813916	2.222621	-1.874581
H	0.294155	0.658935	2.878272
H	-0.605098	-0.753967	2.264036
H	0.557439	-0.954341	3.602579
H	0.380776	-2.568457	-1.623980
H	0.956385	-1.426245	-2.870921
H	1.861803	-2.938878	-2.558530

[Cu(I)LNO$_2$], conformer 1

E = -2364.621904

N	0.033728	-0.090553	-0.083193
C	0.023260	-0.139858	1.379273
C	1.425311	-0.067490	1.987780
N	2.352771	-1.046227	1.401999
C	3.632537	-0.477557	0.971796
C	3.482977	0.544955	-0.157212
N	2.687410	0.037877	-1.282259
C	1.617963	0.936218	-1.719814
C	0.551427	1.163136	-0.645419
Cu	1.512235	-1.671027	-0.526281
O	1.242151	-3.437808	-1.284645
N	0.877614	-3.465852	-2.509234
O	0.724439	-4.580018	-2.969509
C	-1.246709	-0.473777	-0.663325
C	3.517914	-0.421936	-2.387805
C	2.527086	-2.217017	2.253772
H	1.834681	0.944047	1.864139
H	1.339584	-0.212670	3.078061
H	-0.445787	-1.093142	1.669076
H	-0.596894	0.668749	1.817094
H	0.961201	1.784590	0.162220
H	-0.263356	1.762901	-1.085927
H	1.147495	0.475743	-2.602582
H	2.011416	1.921226	-2.044027
H	3.024666	1.465207	0.229533
H	4.491572	0.843163	-0.491134
H	4.257196	-1.313465	0.620238
H	4.176357	-0.002306	1.813303
H	3.121340	-2.976952	1.727109

H	3.033950	-1.971421	3.208080
H	1.546659	-2.656810	2.484416
H	-2.062584	0.222431	-0.384228
H	-1.166500	-0.491657	-1.759344
H	-1.518470	-1.485959	-0.331383
H	4.070318	0.406511	-2.874429
H	4.247500	-1.158021	-2.021621
H	2.892032	-0.918844	-3.142461

[Cu(I)LNO$_2$], conformer 2

E = -2364.626131

C	0.040252	0.014928	-0.007617
C	0.046083	0.072608	1.520276
N	1.396037	0.023749	2.080652
C	2.125412	-1.223907	1.823809
C	3.527740	-0.980575	1.258257
N	3.528938	-0.091938	0.097234
C	2.811072	-0.603339	-1.073241
C	1.830521	0.423118	-1.646545
N	0.947534	1.002840	-0.624053
Cu	2.341039	1.618233	0.880821
N	3.184416	3.115115	1.758175
O	4.395890	3.329889	1.630878
O	2.547993	3.807242	2.562391
C	0.214024	2.158461	-1.139330
C	4.856281	0.415446	-0.225177
C	1.439685	0.425841	3.480462
H	0.318034	-0.992828	-0.343396
H	-0.993179	0.164190	-0.361334
H	-0.408350	1.020511	1.848690
H	-0.597431	-0.742257	1.909492
H	1.546338	-1.851683	1.132861
H	2.220027	-1.821629	2.746457
H	4.148136	-0.501119	2.031841
H	3.997114	-1.960030	1.033250
H	2.275965	-1.523981	-0.804250
H	3.511834	-0.899425	-1.872690
H	2.396721	1.254234	-2.094452
H	1.249140	-0.048031	-2.463562
H	0.920904	2.921394	-1.493149
H	-0.458749	1.883298	-1.974612
H	-0.389954	2.601625	-0.335780
H	0.834791	-0.236706	4.130337
H	2.479571	0.405476	3.835874
H	1.078765	1.459146	3.580466
H	5.573483	-0.395165	-0.462509

H	4.790822	1.084799	-1.094731
H	5.236502	1.014762	0.613648

[Cu(II)LNO$_2$]$^+$, conformer 1

E = -2364.460202

N	0.022808	-0.015126	-0.048235
C	0.001744	-0.033506	1.436597
C	1.414733	-0.076084	2.008905
N	2.220088	-1.129235	1.351153
C	3.528004	-0.671739	0.848112
C	3.420832	0.380886	-0.259173
N	2.530856	-0.020674	-1.373693
C	1.544811	1.012457	-1.738253
C	0.562827	1.259648	-0.600429
Cu	1.271354	-1.503421	-0.507621
O	2.063491	-3.164890	-0.929389
N	1.761287	-3.444691	-2.164170
O	2.070786	-4.544678	-2.516397
C	-1.306212	-0.318328	-0.614224
C	3.287123	-0.501639	-2.533397
C	2.345553	-2.338289	2.172382
H	1.913773	0.894266	1.890975
H	1.357811	-0.251337	3.093209
H	-0.550612	-0.934815	1.741005
H	-0.549970	0.838427	1.825034
H	1.044419	1.817827	0.210591
H	-0.263805	1.892518	-0.953327
H	1.009266	0.654388	-2.629861
H	2.034465	1.963036	-2.014133
H	3.074267	1.335020	0.156882
H	4.434073	0.577926	-0.639868
H	4.049646	-1.560458	0.463127
H	4.145078	-0.259040	1.665568
H	2.807798	-3.137071	1.578066
H	2.959734	-2.148663	3.069177
H	1.348660	-2.674138	2.486697
H	-2.045086	0.429430	-0.284675
H	-1.250558	-0.305356	-1.710621
H	-1.636592	-1.313008	-0.287016
H	3.874547	0.312433	-2.991132
H	3.969559	-1.301433	-2.218071
H	2.597931	-0.914256	-3.281738

[Cu(II)LNO$_2$]$^+$, conformer 2

E =-2364.463565

C	0.044050	0.026833	0.015260

C	0.051659	0.084734	1.541031
N	1.412480	0.049964	2.092727
C	2.140500	-1.205093	1.830326
C	3.532676	-0.954917	1.249323
N	3.511066	-0.030077	0.093117
C	2.801659	-0.583480	-1.090727
C	1.813540	0.423794	-1.666715
N	0.969741	1.012868	-0.602429
Cu	2.383502	1.522797	0.779418
N	3.348297	2.907822	1.772741
O	4.256355	3.455385	2.319804
O	2.212419	3.429471	1.631493
C	0.249578	2.206888	-1.072655
C	4.865647	0.433563	-0.251235
C	1.448858	0.435124	3.503675
H	0.315048	-0.978590	-0.329003
H	-0.980340	0.198785	-0.346500
H	-0.411956	1.024487	1.877712
H	-0.573885	-0.739737	1.930204
H	1.552051	-1.838582	1.153831
H	2.253393	-1.792926	2.754671
H	4.176622	-0.496749	2.014444
H	3.996411	-1.915153	0.965195
H	2.288670	-1.508021	-0.800328
H	3.524929	-0.871581	-1.867180
H	2.359157	1.249953	-2.145392
H	1.195389	-0.055901	-2.444829
H	0.965921	2.959573	-1.427354
H	-0.440093	1.951523	-1.894051
H	-0.326696	2.636735	-0.243178
H	0.872523	-0.263534	4.136268
H	2.489473	0.450171	3.855522
H	1.034480	1.445774	3.622971
H	5.511793	-0.416389	-0.525243
H	4.811563	1.129101	-1.098716
H	5.306922	0.959679	0.605243
